# The human amygdala disconnecting from auditory cortex preferentially discriminates musical sound of uncertain emotion by altering hemispheric weighting

**DOI:** 10.1038/s41598-019-50042-1

**Published:** 2019-10-15

**Authors:** Francis A. M. Manno, Condon Lau, Juan Fernandez-Ruiz, Sinaí Hernandez-Cortes Manno, Shuk Han Cheng, Fernando A. Barrios

**Affiliations:** 1 School of Biomedical Engineering, Faculty of Engineering, The University of Sydney, Sydney, New South Wales Australia; 2 Department of Physics, City University of Hong Kong, HKSAR, China; 3 Departamento de Fisiología, Facultad de Medicina, Universidad Nacional Autónoma de México, México City, 04510 Mexico; 4 Department of Biomedical Sciences, City University of Hong Kong, HKSAR, China; 5 Instituto de Neurobiología, Universidad Nacional Autónoma de México, Juriquilla, Querétaro Mexico

**Keywords:** Perception, Amygdala

## Abstract

How do humans discriminate emotion from non-emotion? The specific psychophysical cues and neural responses involved with resolving emotional information in sound are unknown. In this study we used a discrimination psychophysical-fMRI sparse sampling paradigm to locate threshold responses to happy and sad acoustic stimuli. The fine structure and envelope of auditory signals were covaried to vary emotional certainty. We report that emotion identification at threshold in music utilizes fine structure cues. The auditory cortex was activated but did not vary with emotional uncertainty. Amygdala activation was modulated by emotion identification and was absent when emotional stimuli were chance identifiable, especially in the left hemisphere. The right hemisphere amygdala was considerably more deactivated in response to uncertain emotion. The threshold of emotion was signified by a right amygdala deactivation and change of left amygdala greater than right amygdala activation. Functional sex differences were noted during binaural uncertain emotional stimuli presentations, where the right amygdala showed larger activation in females. Negative control (silent stimuli) experiments investigated sparse sampling of silence to ensure modulation effects were inherent to emotional resolvability. No functional modulation of Heschl’s gyrus occurred during silence; however, during rest the amygdala baseline state was asymmetrically lateralized. The evidence indicates changing hemispheric activation and deactivation patterns between the left and right amygdala is a hallmark feature of discriminating emotion from non-emotion in music.

## Introduction

Elucidating the threshold of emotional versus non-emotional sound will aide our understanding into the basic properties of emotion. What is a sound that conveys emotion and the neural substrates involved in interpreting emotional from non-emotional sound have not been previously evaluated. Emotions used in previous experiments were selected for being certain^1–9^, or altered in arousal and valence^10–12^, as opposed to emotion altered in their frequency or envelope components (physical basis of sound). For example, unpleasant sound can be distinguished from pleasant sound based on differences in primary auditory cortex activation patterns^4,5,9,13–17^. No study to-date has asked how a minimally emotional sound, such as music, differentially activates neural substrates^1–3,8,18–20^. Further, how the auditory components, such as fine structure (FIS) and envelope (ENV), confer emotion to sound are unknown^21–23^. In the present series of experiments, we sought to identify what attributes of sound confer an emotion, the underlying neural processes in distinguishing emotion from non-emotion, and demonstrate how these processes are different at threshold, where the subtlety of emotion is of interest^1,2,5–7,18,19^.

How is an emotion interpreted from sound? Frequency and amplitude modulations (i.e. FIS and ENV) encode all acoustic information^24–26^. Sound information is functionally represented in the primary auditory cortex (A1)^27^ as a tonotopic map from high-to-low-to-high frequency^28,29^. The mirror tonotopic functional map follows the anatomical variability associated with Heschl’s gyrus (HG)^30,31^. Functional mapping of A1 along the structure of HG has revealed^29,32,33^ right and left hemispheric laterality of spectral versus temporal favoring cues, respectively^34^. Dorsal-ventral processing streams^35,36^ and pitch discrimination thresholds are oriented along the posterior-to-anterior subdivisions of the primary auditory cortex^37^. In the auditory cortex, differing contributions of FIS and ENV are utilized to identify emotional content in speech^38^. These varying acoustic components within the prosodic contours of pitch^39^ are thought to be processed in a right-hemispheric lateralized manner^40,41^. Emotional sound activates the amygdala^42,43^, however, investigations as to which components (FIS or ENV) are coupled to amygdala activation have not been explored. We postulated music emotion would be a similar auditory perception^19,44^ to the emotion carried in speech (i.e. prosody)^9,38,44–48^. Here, the amygdala and/or HG activation are expected to be functionally right-lateralized for identifying emotional attributes in sound at the limit of discrimination. Further, the activations are expected to use fast varying components of fine structure to identify emotion at threshold.

How does emotion elicit neural responses distinguishable from non-emotion? Current research points to amygdala (AMG) activation patterns^23,42^, where AMG subnuclei are thought to play specific roles in regulation of emotional stimuli^7^. Damage to the AMG disrupts recognition of scary and fearful emotion^42,49–51^. Left hemispheric AMG damage results in impairment of vocal emotion recognition whereas right hemispheric AMG lesions have spared emotion processing^11,52^. Despite these findings, the specific contributions of the left or right AMG to emotional processing are contested^1,8,10,11,44,50–52^. Moreover, how the AMG works in concert with other regions such as HG^11^, when emotional signals are at the boundary of the emotion/non-emotion threshold, has not been adequately evaluated^10,44^. Several theories have outlined differences in right/left hemispheric lateralization or sex differences due to preferential hemispheric dominance^53–55^. Interestingly, previous research has revealed a right/left hemisphere bias in favoring FIS versus temporal cues, respectively^34,56^. While we currently know very little about the neural basis of sex differences in emotion perception^10^, or understand fully the contributions to hemispheric lateralization^10,34,39–45,53^, assessing uncertain emotion at threshold on a range of levels is the key to elucidating the neural components for distinguishing emotional from non-emotional sound. When an uncertain emotion becomes chance identifiable and a neural response follows this pattern, tuning in to emotional signals and off to non-emotional signals, a clue is given as to how emotion perception occurs.

How do humans determine emotional uncertainty in music? Uncertainty can be thought of as a high entropy state with low precision^57^, whereas a high precision, low entropy state would be considered certain. Kolesch *et al*. 2019 suggested a hierarchical predictive coding process in the auditory system where top-down predictions descend down cortical hierarchies to resolve prediction errors at lower levels^57^. Put another way, sensory information that does not match a prediction is corrected by top-down descending projections. Here the authors argue an active inference process occurs whereby the perception from brainstem to cortex minimizes predictions errors^57^. In music, the authors argue this is the basis for surprise or irregular events^57^. Prediction of uncertain information forms the basis for decoding aspects of threshold and minimal emotion necessary for decision making^1^. Similarly, a recent study used multivoxel pattern analysis, a type of linear support vector machine (SVM) classifier, to predict emotion-specific neural responses in voice based on musical instruments, and vice versa^58^. The authors concluded that emotion-specific patterns generalize across sounds with different acoustical properties. In the present series of experiments, uncertain emotion was used to determine the functional modulatory response of HG and AMG, and we expected this would be a unique functional state compared to certain emotion.

**Figure 1:**
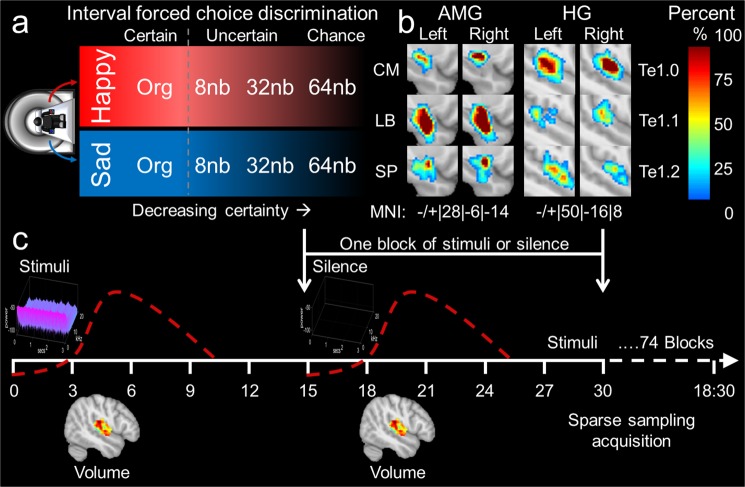
Experimental Design. (**a**) Happy/sad descending interval forced-choice discrimination task. The original, 8 nb, 32 nb, and 64 nb represent band decompositions of increasingly uncertain emotion. (**b**) Amygdala and HG subdivision 95% confidence interval probabilistic maps^55,59^ with approximate MNI coordinates below. Subdivisions for Heschl’s gyrus (HG): middle HG (Te1.0), posteromedial HG (Te1.1), and anterolateral HG (Te1.2). Subdivisions for amygdala: laterobasal (LB), centromedial (CM), and superficial (SP). Color bar represents 0–100% probability of finding the ROI. In the present experiments 95% CI were utilized for the ROI. (**c**) Single volume sparse sampling paradigm (see methods for details). Two runs of 74 blocks (40 emotional sound stimuli and 34 silent stimuli presentations) were conducted by acquiring one volume (≈1 s) per sparse sampling block. The volume acquisition was separated from emotional stimuli (3 s) or silence (control stimuli) by approximately 100 ms followed by ≈10.00 seconds of silence. Two types of blocks were presented with original or uncertain emotion stimuli or silent stimuli.

Our aim for the present series of experiments was to investigate the functional response to certain and uncertain sound interpreted as emotional^20^. A two-interval forced-choice discrimination paradigm was used with sparse sampling functional magnetic resonance imaging (fMRI) to investigate HG and AMG subdivisions^59,60^ during identification of emotional signals of varying certainty. Happy and sad classical music stimuli were combined in FIS or ENV band number (nb) domains by a band-wise decomposition process (herein, decomposition^17,22,26^; Figs 1a, S1, S4; Tables S1 and S2). One psychophysical experiment was conducted presenting stimuli binaurally with four separate in-scanner sparse sampling experiments (Fig. S1): (1) resolving uncertain from certain emotional sound (n = 16); (2) ascertaining the contribution of sex to emotional resolvability (n = 12) with two follow-up sparse sampling experiments of silence utilized as negative controls to determine baseline region of interest (ROI) modulatory state (n = 15); (3) complete silence modeling (i.e., baseline); and (4) interleaved silence modeling. First, we expected that varying FIS or ENV information essential to detecting emotion in sound would result in reduced certainty by increasing decomposition^14,18,22,26,34,38,45^, revealing which cue (i.e., FIS or ENV) was essential for resolving emotional information^22,26^. We conjectured that emotion in music would utilize the same cue to identify the emotion in prosody (i.e., pitch/fine structure)^14,22,26,38^. Second, we expected that the ROI involved in resolving emotional information would functionally modulate with the cue utilized for distinguishing emotion from non-emotion^5,8,9,13–16,23,48^. Third, if hemispheric lateralization is a feature in resolving certain from uncertain emotion^10,40,41,44,52,53^, or if sex differences in hemispheric lateralization exist^10,53^, we would expect the neural response pattern to vary with the psychophysical identification. The differences would be between sex and/or the left and right hemisphere^61–63^. Lastly, to understand baseline AMG or HG modulation inherent to hemispheric lateralization^10,41,51–53,64,65^, functional patterns during silence were analyzed. Here, the negative control experiments would bolster the results of our emotion resolvability experiments. Our overarching aim was to determine the functioning of HG and AMG during the threshold of emotional response (i.e. the minimum emotion necessary to be defined as emotional).

## Methods

### Experimental design and participants

The psychophysics study (n = 75, age 22.05 ± 3.46 years, range: 18 to 32 years; 33 females) stimuli set included sounds from 14 categories of increasing emotional uncertainty, which were original excerpts (happy or sad) with 2 nb, 4 nb, 8 nb, 16 nb, 32 nb, and 64 nb decompositions (nb – number band; Tables S1 to S6; Fig. 1b)^13,26^. The fMRI study (n = 16 right-handed volunteers, age 27.12 ± 3.08 years, range: 18 to 32 years; 6 females) stimuli set included sounds from eight categories, which were original excerpts (happy or sad) with 8 nb, 32 nb, and 64 nb decompositions (Figs S1 to S4) selected based on the psychophysical response (Fig. S5; Tables S7 to S12). The bilateral sex difference study (n = 12 right-handed volunteers, age 27.66 ± 3.47 years, range: 22 to 32 years; 6 females) included the same stimuli set as in the bilateral study (Figs S1 and S4), selected based on the psychophysical response (Fig. S5; Tables S7 to S12). The negative control experiments, baseline and interleaved silence studies (Figs S1 and S2), consisted of n = 15 self-reporting right-handed volunteers, age 27.62 ± 3.21 years (range: 22 to 33 years; 6 females). Participants had no musical training. All individuals were self-reporting right handers, filling forms with the right-hand. Right-handedness was determined by asking individuals if they ever did anything solely with their left hand. Groups were matched for age, sex, and education. Subjects were native Spanish speakers reporting normal hearing, which was confirmed during an initial verbal screening and audio level setting within the scanner. All participants underwent audiometric testing consisting of presenting and confirming the hearing of a series of pure tones from 400 Hz to 8,000 Hz, in addition to linear sweeps, log sweeps, and white noise in the same frequency range. No subject reported a history of neurological or psychiatric illness. All volunteers gave informed consent and were free of contraindications for MRI scanning. The research protocol was approved by the Ethics Committee on the Use of Humans as Experimental Subjects at the Instituto de Neurobiología of the Universidad Nacional Autónoma de México in accordance with the Declaration of Helsinki, 2013.

### Acoustic stimuli construction

#### Emotional acoustic stimuli: band-wise decompositions (BWD)

Instrumental excerpts of classical piano known to evoke categorically a sense of sadness or happiness were taken from a previous study (www.brams.umontreal.ca/peretz; Peretz *et al*.^13,49,66^). Sad and happy excerpts were decomposed by a BWD Hilbert transformation to derive altered stimuli^26^,^66^ from either emotional category^17,26,66^. Stimuli consisted of homogenous FIS and ENV information due to the decomposition, restriction to 80–4400 Hz, curtailment to 3 seconds, and reconstruction process. Silent stimuli were used as control (Fig. S2, silent stimuli used for silent sparse sampling) and presented in a sparse sampling block design (Fig. S3, finalized sparse sampling design). The stimuli for examination consisted of excerpts in the FIS domain or ENV domain for number band (nb) decompositions 2 nb, 4 nb, 8 nb, 16 nb, 32 nb, and 64 nb for psychophysics, and 8 nb, 32 nb, 64 nb for fMRI scanning (Fig. 1)^22,26^. For psychophysics, 32 original unaltered excerpts were utilized, along with 192 stimuli alterations (a total of 224 stimuli). For fMRI examination 10 original unaltered excerpts where utilized, along with 30 stimuli alterations (a total of 40 stimuli). Example stimuli spectrogram and Fourier Transforms from happy and sad excerpts can be found in Fig. S4. The emotional stimuli of varying certainty were utilized for psychometric testing to ascertain the limit of perception for certain and uncertain emotion^22,26,66^.

#### The two-interval forced choice task

For psychophysics and fMRI experiments, altered emotional excerpts and original excerpts were presented in a two-interval forced choice task (2IFC). Subjects were instructed to respond indicating the excerpt was happy or sad. The two-interval forced choice task was based on the study by Binder *et al*.^67^, where the authors were able to correlate the decision-making processes behind auditory object identification using the 2IFC task to determine sensory threshold of the stimulus. The power of signal detection theory in the 2IFC enables one to discern threshold of our auditory responses^68,69^. All excerpts for the 2IFC task were categorized in reference to the original unaltered excerpt. Figure S4 depicts example stimuli with representative stimuli spectrogram for FIS components and fast Fourier transform (fft) for ENV components. Stimuli were manipulated in MATLAB (Mathworks, Natick, MA) using custom scripts and previously published extensions^22,26^. Supplementary information Table S2 depicts acoustic stimuli and their respective decompositions. Blocks of silence were used as negative controls and devoid of envelope and fine structure information (Fig. S2). Stimuli were presented during psychophysics and fMRI scanning utilizing MATLAB (MATLAB and Statistics Toolbox Release 2012b, The MathWorks, Inc., Natick, Massachusetts) with the Psychophysics Toolbox extension^70^. Iimplemention was on a HP pc (Intel Core i5-4210U CPU at 2.40 GHz) with a RealTek High Definition Audio card using Stereo Mix (RealTek).

### Image acquisition

Images were acquired on a 3T MR750 scanner with a 32-channel coil using parallel imaging with an acceleration factor of 2 (General Electric, Waukesha, Wisconsin). Acquisition was bottom-up interleaved. A Fast Spoiled Gradient Echo BRAin VOlume imaging (FSPGR BRAVO) structural image was acquired for co-registration with functional volumes. The structural image was 3D T1-weighted with resolution of 1 × 1 × 1 mm³, field of view (FOV) = 25.6 × 25.6 cm, slice thickness = 1 mm, time repetition (TR) = 8.156 s, echo time (TE) = 3.18 ms, inversion time (TI) = 450 ms, and flip angle = 12°. Functional volumes consisted of 34 slices (3 mm thick) acquired with single shot gradient-echo echo-planar imaging (GE-EPI) with the following parameters: FOV = 256 × 256 mm^2^, matrix = 128 × 128 (yielding a voxel size = 2 × 2 × 3 mm^3^), TR = 15000 ms, TE = 30 ms, and flip angle = 90°.

### Experimental design and fMRI stimuli presentation

#### Experimental design of the functional MRI protocol

The order of the scans acquired was first localization and then an individualized sound level adjustment, followed by: (1) first fMRI run using a GE-EPI with left button presses for indicating *sad* and right button presses for indicating *happy*; (2) a sagittal T1 weighted FSPGR BRAVO structural image; and (3) the second fMRI run GE-EPI, with left button presses for indicating h*appy* and right button presses for indicating s*ad* (total scan time ≈1 hour). The first fMRI run’s responses were effectively counterbalanced by the second fMRI responses. In addition to the structural scan, a 5 min rest was given between the first fMRI run and the second fMRI run.

The effect of scanner noise on auditory processing is well documented^71–74^. Therefore, we used a sparse sampling paradigm for stimuli presentation optimized to auditory functioning^74,75^. After optimization, the protocol was 74 blocks, with 40 sound stimuli presentations and 34 silent periods (45.95% silent blocks), for a total run time of 18 minutes and 30 seconds. The finalized functional protocol consisted of collecting one volume separated from stimuli by approximately 100 ms, followed by ≈10.00 seconds of silence (Fig. S3). To optimize the sparse-sampling protocol for the most robust BOLD response within Heschl’s gyrus, 29 individuals (mean = 27.711 yr ± 3.077 yr) were assessed prior to implementing the experimental design for the fMRI emotion resolvability experiments.

#### Functional-MRI block stimuli presentation

Per subject, two runs were collected with each series of volumes divided into 8 sets of stimuli blocks. There were 5 stimuli presentations per block followed by a period of silent presentations (3 or 4 silent stimuli per silent block). A total of 40 sound stimuli presentation per individual per run were performed. For the 16 fMRI scanned individuals, a total of 1280 different stimuli presentations were performed in the fMRI experiment. Stimuli of a like kind were aggregated and analyzed as the unit of analysis (i.e. block aggregation of alike stimuli^37^, from which a whole head statistical map was derived). Each averaged whole head statistical map was a representation of those stimuli aggregations from each category (i.e., original happy, happy-8 nb, happy-32 nb, happy-64 nb, original sad, sad-8 nb, sad-32 nb, and sad-64 nb). The number of silent blocks was 34 per run and 68 per individual, and 1088 different silent periods in the fMRI experiment. Please see “AMG is asymmetrically lateralized at rest, left leaning is less deactivated than right leaning” for the negative control experiment concerning silence sparse sampling^75^. During stimuli presentation or silent periods, subjects kept their eyes open and stared into a mirror. Blocks of stimuli interspersed by blocks of silence were separated from differing blocks of stimuli by at least two minutes, thus treated equally for habituation across stimuli category^64^. The fMRI experiments used Matlab and the Psychophysics Toolbox extension^70^ to generate the stimuli during fMRI scanning at a sampling rate of 44.1 kHz. Stimuli were delivered via MRI-compatible headphones (AudioSystem, Nordic NeuroLab). Participants performed this task with accuracy (mean 89.21% CI: 1.25%; Table S1). Stimuli ratings were within percent identification ranges of former functional studies^15,23,76–78^ and psychological studies out of-scanner (i.e. emotion identification task)^49,79–82^.

### Functional image processing and ROI segmentation

#### Functional image processing

Image processing was carried out using fsl tools (fMRIB, University of Oxford, UK) and FEAT (FMRI Expert Analysis Tool) version 5.98. The statistical analyses utilized the general linear model (GLM) to assess the relationship between our categories/explanatory variables (EV., i.e. regressors) and the dependent variable (i.e., the BOLD signal)^83^. A standard GLM was utilized to obtain averages from the 8 category regressors and six motion regressors (three rotation and three translation^19^, using the double gamma function convolved with the hemodynamic response function (HRF)^83^. Higher-level analyses were performed using FLAME (FMRIB’s Local Analysis of Mixed Effects)^84,85^. In detail, single-subject first level analysis was performed using a design matrix consisting of 8 EV’s representing each of the categories presented in the experiment (i.e., original happy, happy-8 nb, happy-32 nb, happy-64 nb, original sad, sad-8 nb, sad-32 nb, and sad-64 nb). A fixed-effects analysis was utilized to average category scans between a single-subject’s first fMRI run and second fMRI run. A mixed-effects higher level analysis was performed to average response categories (original happy, etc.) across subjects. Correction for multiple comparisons was carried out using random field theory (voxel z > 2.3, cluster p < 0.05)^86^. Figure S1 details the experimental design: sparse sampling paradigm parameters utilized to optimize the experiment, run format, analysis of explanatory variables, and details of image processing. Functional volumes were preprocessed for motion correction, linear trend removal, spatial smoothing using a 5 mm FWHM Gaussian kernel, and elimination of low-frequency drifts using a temporal high-pass filter with a cutoff of 100 s. Preprocessing of the fMRI statistical maps included spatial realignment, coregistration with anatomical data using fsl FLIRT, and spatial normalization and alignment with MNI 152 T1-weighted MRI scans^87^.

#### Segmentation of ROI by probabilistic mapping

The region of interest (ROI) segmentation utilized the Jülich histological atlas^31,59,60^ to derive 95% confidence interval probability maps associated with the subnuclei. Therefore, the ROIs consisted of overlaps with a preference for inclusivity^31^. The ROI segmentation was aligned and registered to MNI 152-T1 standard space and MNI 152-T1 standard symmetrical space. The ROI probability maps for delineating auditory cortex were TE1.0 middle HG, TE1.1 posteromedial HG, and TE1.2 anterolateral HG^60^. For amygdala, the ROIs were laterobasal (LB), centromedial (CM), and superficial (SP)^59^. The activation and deactivation for statistical analyses was based on ROI + ≥100 voxels^59,60^. Modulation was calculated as normalized t-value change of ROI by stimuli (i.e. EV). When modulation was used for emotion identification, calculation was as a factor of percent change by decomposition from the original stimuli.

### Hemispheric lateralization

#### Symmetrical map

Hemispheric lateralization was derived by making the brain symmetrical and comparing common areas between the left and right hemisphere^88,89^. A symmetric MNI template was constructed for hemispheric lateralization statistics. Our data was registered to this MNI-symmetrical template using fsl’s ApplyXFM trilinear interpolation transformation with 6 degrees of freedom (1 rotation +2 translation +2 scale +1 skew). The output map was a nii functional map in MNI symmetrical space. Where ROI probability maps pertain to asymmetrical areas of left and right hemisphere^59,60^, the symmetrical map accounts for those differences by the output of the affine trilinear interpolation transformation. The asymmetrical map was created by taking the original right (Rorg) and original left (Lorg) side hemisphere for an symmetrical side (ss) and comparing by [Rorg] − [Lorg] = Rss and [Lorg] − [Rorg] = Lss ([] denoting absolute). The Rss and Lss symmetrical counterparts in the final ROI extraction of [Rss] and [Lss] by: *left* side Te1.0, Te1.1, and Te1.2; *right* side Te1.0, Te1.1, and Te1.2; *left* side LB, CM, and SP; and *right* side LB, CM, and SP^88^ were compared. For each resultant volume output, hemispheres were restricted in their t-value changes (positive or negative) and then folded along the longitudinal axis. Each hemisphere was flipped over and the shortest hemisphere was taken as reference, using the x-axis length of each hemisphere from midline. Then the left ≠ right difference was computed to determine the absolute difference between the left or right hemisphere directionally. Lastly, the matrix was written to nifti file for visualization using xjview and Matlab.

#### Hemispheric lateralization of emotion indices

The hemispheric laterality of emotion was determined using a hemispheric lateralization index to assess activation and deactivation t-value changes independently for the left and right hemispheres. The hemispheric lateralization index restricted the absolute positive (eg. 0 to positive numbers, effectively activation herein) and negative (eg. negative numbers to 0, effectively deactivation herein) t-value changes. Whole head statistical maps were made symmetrical for each of 8 EV’s, restricted in the positive or negative range, and finally co-registered to a symmetrical MNI template. This allows direct left versus right voxel comparisons. The activation hemispheric laterality calculations reflect [R]-[L]. The resultant difference for activation calculations was a positive t-value activation reflecting a right hemisphere sided laterality and a negative t-value deactivation reflecting a left hemisphere laterality. The deactivation hemispheric laterality calculations reflect [−R]-[−L], with numbers in the negative range reflect the opposite observation. The resultant difference for deactivation calculations was a negative number reflecting a right hemisphere sided laterality and a positive number reflecting a left hemisphere laterality. The hemispheric laterality calculations were separated into groups to distinguish the direction (activation and deactivation) and magnitude of lateralization separately.

### Psychophysical analysis correlated with functional modulation

Psychophysics was analyzed with an analysis of variance (ANOVA) using the stimuli categorized as happy or sad as the factor variable and the original, 2 nb, 4 nb, 8 nb, 16 nb, 32 nb, and 64 nb decompositions as response variables. Follow-up testing, where appropriate, was performed with a paired student’s t-test. The question driving the psychophysical statistical testing was to determine if category was modulated by emotion (i.e. happy or sad), and whether band decomposition revealed a trend (modulation by band, i.e. nb). To determine the ROI BOLD modulatory properties of decomposed happy or sad stimuli, activation/deactivation map ROI t-values by emotion identification were evaluated. An ANOVA was utilized to determine the interaction between the ROI functional response by t-value change and band decompositions (org, 8 nb, 32 nb, and 64 nb) as the factors. Follow-up comparisons were made with a Student’s *t* test to determine the significance of the difference in the previous correlations from zero for hemispheric lateralization calculations and from zero for gender differences. The psychophysics was to determine category of response (happy or sad) to the stimuli while the fMRI analysis was to determine modulation of the ROI with the stimuli (i.e. change in functioning of ROI with stimuli).

Weighted modulation was calculated to compare the values of HG and AMG responses (Equations S1, S2, and S3). Modulation was calculated as the absolute percent difference in decomposition category (psychophysical stimuli category change) as a factor of the absolute change in t-value of ROI. The term was *weighted* as function of the original percent identification and t-value of the ROI in question. For example, weighted modulation was the absolute change in t-value of HG and AMG by the absolute difference in decomposition categories (original – 8 nb, 8 nb – 32 nb, and 32 nb – 64 nb), averaged to create a mean for each ROI. The psychophysical emotional identification response was also calculated as an absolute change value by the absolute difference between original – 8 nb, 8 nb – 32 nb, and 32 nb – 64 nb decompositions in emotional identification (i.e. the psychophysical response in percentage). The differences were then averaged to create a mean category change in emotion identification. Note that there is only one emotional identification by category (happy or sad). Therefore to make this unique for each ROI as a factor of the psychophysical profile, the absolute emotion identification was divided by the absolute t-value change for the ROI in question. Both these absolute values were plotted to determine absolute changes by hemisphere and ROI in the same figure. Dividing all values as a function of the original t-value puts values in perspective of change from original ROI functioning. Where stated, the standard deviation (SD) was calculated in a similar way from percent change statistics (Equations S1 to S3).

The psychophysical-fMRI statistical null hypothesis driving the experiments was the BOLD signal change for emotion (i.e. happy or sad), categorized in FIS or ENV by decomposition, having no discernable affect modulating the delineated ROI. The alternative hypothesis was accepted as significant at p < 0.05. Categories were separated into certain (original excerpts), uncertain (<80%), and chance (<50%). Based on psychophysics (Table S1; Fig. S5; Tables S3 to S12), the 8 nb and 32 nb were considered uncertain and 64 nb was considered chance based on their percent identification with the original excerpts. Modulation was defined as a percent BOLD change between categories averaged over the set of common stimuli for happy or sad emotions and determined as a difference from the original stimuli. Statistics were calculated based on standardized formula^90^ using custom scripts. Activation maps were visualized in Matlab using xjView toolbox (http://www.alivelearn.net/xjview) using projections to MNI 152 T1-weighted MRI^87^ with custom scripts.

### Sparse sampling trails to capture the HRF

To optimize the sparse sampling protocol, we reduced the number of silent blocks (used for stimuli comparisons), gap periods (durations of silence occurring after and before volume acquisition/stimuli presentation), and time repetition (time of one entire block repeats) as described in Fig. 1a^74,75^. Sparse sampling is the acquisition of functional volumes interspersed with silence within a stimuli block and silent periods within a block of silent presentations^71–74^. To determine the most robust hemodynamic response function (HRF) to our 3-second emotional stimuli, we have manipulated three variables in the generic sparse sampling protocol to optimize the paradigm^74,75^. The manipulated variables were: (1) the gap delay (silent), occurring between the end of our 3-second musical stimuli and beginning of the acquisition time (TA), (2) the duration of the silent period (silent), occurring after the TA and prior to the new stimuli presentation, and (3) the repetition time (TR). Both the periods, which were silent, were manipulated by changing the onset of stimuli presentation. The gap delay was altered from 1 sec to 3.5 sec in 0.5 sec or 0.25 sec intervals. The TR = 13 sec was unaltered for the gap delay manipulations. The silent period durations were altered from 8 sec to 5 sec by 3 sec intervals, resulting in TR alterations of 13 sec and 10 sec. Periods of TR were manipulated for TR = 13, TR = 12, TR = 11, and TR = 10 sec (see subplot b in Fig. S1).

### Stimuli for sparse sampling optimization

Our design matrix for analyzing the preliminary sparse sampling data was to aggregate all stimuli events of a similar kind versus non-events which were silence/no-stimuli presentation (see Fig. S2). This was our primary approach for discerning activation/deactivation. A final protocol was analyzed as described above. A variety of stimuli were used to assess the HRF and determine activation of the auditory cortex. These were used during our stimuli assessment to determine maximum auditory cortex activation for our musical stimuli. We used our generic stimuli as discussed, and three specific stimuli generated with Matlab^91^, to activate the central auditory cortex during our sparse sampling preliminary study: (1) linear sweep with frequency range of 440 to 7040 Hz, the 16th Harmonic of A4, (2) log sweep with frequency range of 440 to 7040 Hz, and (3) white noise. Stimuli spectrogram and fast Fourier transform (fft) figures within the manuscript were restricted and normalized to a common frequency and amplitude range based on the entire set of stimuli (Fig. S4). The original Beethoven excerpt from the Piano Concerto No. 4 (3rd mvt), categorized as happy (stimuli 03), and the original Albinoni excerpt from the orchestral Adagio in G min, categorized as sad (stimuli 01), were described in detail as representations (Fig. S4). All stimuli were generated with Matlab^91,92^ and tested on an HP pc (Intel Core i5-4210U CPU @2.40 GHz) with a RealTek High Definition Audio card using Stereo Mix (RealTek) Driver. Stimuli were tested in a psychophysics protocol as described above and fMRI as described in the respective sections.

### Statistical comparisons

Detailed analyses, hypotheses, and effect sizes are found in the Supplementary Information. The study consisted of 5 experiments (Fig. S1): (1) psychophysics of emotion discrimination (n = 75), (2) fMRI evaluation of certain, uncertain and chance stimuli (n = 16), (3) hemispheric lateralization analysis (N = 12), and negative control experiments (4) complete silence and (5) interleaved sparse sampling of silence. An analysis of variance (ANOVA) and t-test were used for statistical analyses. For experiment 1, an ANOVA was used for determining happy or sad stimuli category by BWD. For experiment 2, an ANOVA was used for determining functional modulatory response by category happy and sad (certain, uncertain and chance). Additionally, an ANOVA was used for assessing subdivision by BWD differences. For experiment 3, an ANOVA was used for assessing subdivision by BWD differences by the left and right hemisphere. For experiments 4 and 5, an ANOVA was used for determining block (even and odd) by hemisphere or subdivision for silent periods. For all experiments, an ANOVA was used for assessing sexual dimorphisms (differences between males and females). Follow-up statistics were conducted with a t-test.

## Results

### Psychophysics of emotion identification reveals sad/happy differences based on fine structure

Information encoded primarily in FIS for happy stimuli and FIS for sad stimuli (to a lesser, albeit still significant, extent; Fig. S5a,b) was relevant for identifying emotion in sound (SI Results; Tables S7–S10). The certainty for happy (*F*_6,15_ = 80.64, *p* < 0.0001) and sad (*F*_6,15_ = 28.76, *p* < 0.0001) stimuli were not homogenous and were significantly different from each other. The average ± SD response between decompositions for happy and sad stimuli was 15.55% ± 1.64% and 12.00% ± 2.17%, respectively (SI Results). The original happy excerpt was significantly different from the happy-32 nb (*F*_5,10_ = 4.10, *p* = 0.028) and happy-64 nb (*F*_5,10_ = 12.97, *p* < 0.001) excerpts. The original sad excerpt was not significantly different for decompositions when correcting for multiple comparisons (SI Results). However, a post-hoc paired t-test revealed sad-32 nb (*t*_15_ = 6.25, *p* < 0.0001) and sad-64 nb (*t*_15_ = 9.74, *p* < 0.0001) were significantly different from the original sad excerpt. We conclude that distinguishing happy and sad acoustic information utilizes fine structure cues to resolve emotional information, but participants rely more heavily on fine structure for happy stimuli than for sad stimuli.

### Functional resolvability of uncertain emotion information elicits AMG deactivation

**Figure 2:**
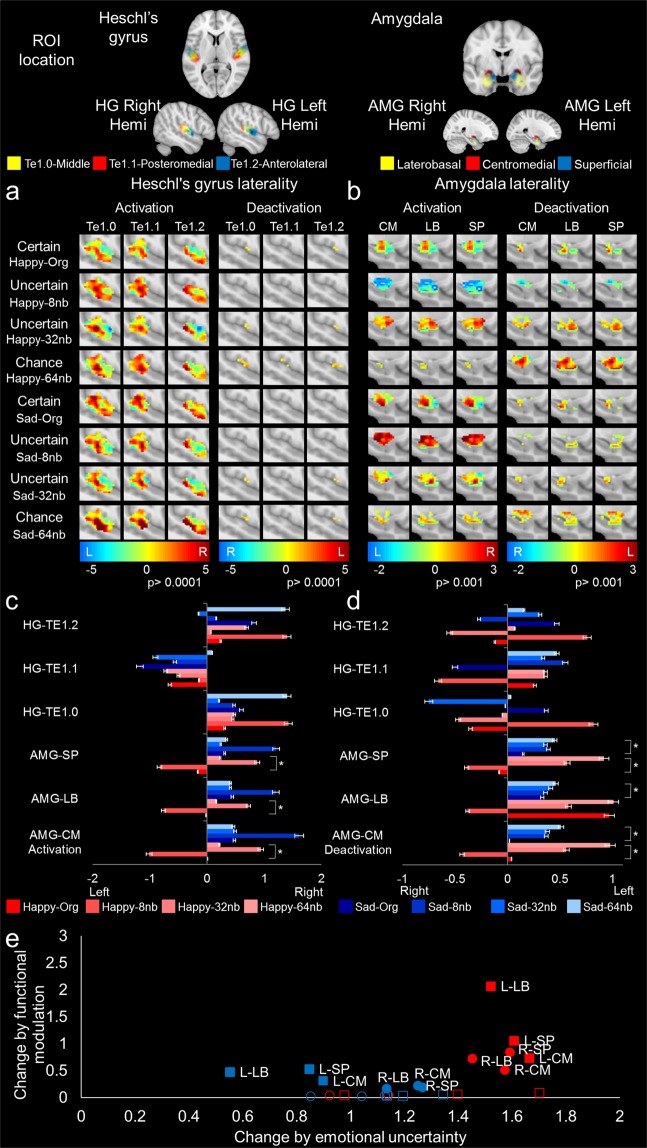
Hemispheric laterality differences during functional resolvability of uncertain emotion utilizing fine structure cues. Hemispheric laterality differences during functional modulation to fine structure cues and weighted modulation by emotional uncertainty as a function of subdivision difference mapping of hemisphere (n = 16, 27.12 ± 3.08 years, range: 18 to 32 years; 6 females). The first row demonstrates the ROI location based on the probability map for right and left HG and AMG by subdivision. (**a**) Heschl’s Gyrus and (**b**) Amygdala laterality subdivisions (Te1.0, Te1.1, Te1.2, LB, CM, SP) within each panel separated by *Activation* and *Deactivation* (SI Methods). The ROI delineations for HG and AMG are found within the column subdivisions. The activation/deactivation color bar range was standardized across decompositions for HG and AMG independently. Error bars represent standard error and asterisk represents significance (p < 0.01). (**c**,**d**) ROI subdivision activation and deactivation, respectively, for decompositions by average t-value. (**e**) Modulation was weighted as the absolute function of the ROI. This was done by dividing the function by absolute percent change in the psychophysics calculations. The average activation map t value derived from the ROI functional volume (y-axis) was correlated with percent identification by decomposition (y-axis) with representation for each ROI grouped (left hemisphere-square; right hemisphere-circle; blue-sad stimuli, red-happy stimuli; AMG-filled; HG-unfilled). The HG-ROI data were unnamed due to overlap, clustering, and little difference (Fig. S8).

Functional MRI revealed FIS-encoded happy or sad stimuli elicited significant modulation of AMG and slight (non-significant) modulation of HG, but only deactivation of AMG was coupled to uncertain emotional stimuli (Figs 2d and S6; SI Results). The slight HG modulation was most likely due to the FIS/ENV responsive properties of HG^29,32,33^. Interestingly, AMG modulation, although different in magnitude, was not a linear decrease by identification as observed for the psychophysical response, but an increase by decomposition followed by a reversal at chance emotion identification (i.e., significant deactivation at 64 nb stimuli preceded by an increase in activation when unsure; far right columns of Fig. S6o through S6p).

#### Heschl’s gyrus follows FIS cues

Emotional stimuli of increasing uncertainty (Fig. S6; ROI S6a through S6d)^60^ non-significantly increased HG response by decomposition with no differences found between subdivisions (Fig. S6g,h). Here, the change in sound information of FIS or ENV cues most likely elicited the change in HG activation^3,17,19–22,24,32,34,35^, and no difference was found resolving uncertain emotional stimuli (see SI Results).

#### AMG response functionally resolves uncertain emotional stimuli, deactivating at chance emotional identification

When emotional stimuli were increasingly uncertain, AMG functioning (Figs 2d and S6; ROI – S4i through S4l)^59^; significantly increased (from the original to 32 nb stimuli) for happy or sad emotions (Fig. S6e,f compared with S6m,n; SI Results). The observed trend, following uncertain emotional identification, ceased when stimuli were identified by chance (i.e., unable to resolve happy or sad emotion as indicated for 64 nb stimuli, for both hemispheres; Fig. S6o,p; last row for each stimulus set and last bar graph set). No significant differences were observed between AMG subdivisions (*p* < 0.05). Nevertheless, significant differences were found by decomposition and hemisphere to happy and sad emotions (Fig. S7). The functional changes for AMG were considerably greater than for HG (percent weighting Fig. S8; Tables S13–S16). An ANOVA found AMG modulation by stimuli significantly different (Fig. 2d; Tables S15 and S16; *F*_3,15_ = 22.12, *p* < 0.001) with functional change from original excerpts by hemisphere as follows: sad emotion by right hemisphere of 84.45% ± 5.59% (*F*_2,6_ = 67.69, *p* < 0.0001), sad emotion by left hemisphere of 67.10% ± 24.48% (*F*_2,6_ = 29.01, *p* < 0.0001), happy emotion by right hemisphere of 232.05% ± 13.06% (*F*_2,6_ = 79.67, *p* < 0.0001), and happy emotion by left hemisphere of 262.71% ± 3.86% (*F*_2,6_ = 140.3, *p* < 0.0001). For weighted modulation comparisons, see Fig. 2e. Right hemisphere functioning was greater than left hemisphere functioning by a factor of three in activation for sad and happy stimuli (Fig. S7). Follow-up testing to investigate the cessation in modulation due to the deactivation observed at uncertain 64 nb stimuli (Figs S4p and S6o) revealed highly significant differences from the original excerpts for: sad emotion right hemisphere response to 64 nb (*t*_3_ = 4.58, *p* < 0.001), sad emotion left hemisphere response to 64 nb (*t*_3_ = 10.74, *p* < 0.001), happy emotion right hemisphere response to 64 nb (*t*_3_ = 6.63, *p* < 0.01), and happy emotion left hemisphere response to 64 nb (*t*_3_ = 5.47, *p* < 0.001).

#### Contralateral hemispheric HG activation balances lateralization to certain emotion. Deactivation of left AMG hemispheric functioning follows uncertain stimuli

Hemispheric lateralization of HG and AMG was investigated due to the observed differences in modulation by hemisphere as a response to certain and uncertain emotion (Figs S6 and S7; SI Results). To model the hemispheric lateralization, a weighted voxel modulation was calculated as the absolute change in emotion resolvability by decomposition, factored by the absolute change in t-value for each ROI by voxel by hemisphere (Fig. 2e). In particular, we were interested in the following questions: (1) Are there side effects when balanced in voxel space by the contralateral hemisphere? (2) How does the pattern of activation and deactivation by hemisphere follow the identification of uncertain emotional stimuli? (3) Is there a difference between ROI modulation by decomposition? (4) Do sex differences exist in hemispheric lateralization to emotion resolvability?

Little variation in t-value modulation by emotional certainty occurs for HG (i.e., difference in activation along the row in Fig. 2a; the magnitude of AMG was greater, Figs 2e and S7). AMG activation in the right and left hemisphere for sad, *F*_2,6_ = 67.69, *p* < 0.0001 and *F*_2,6_ = 29.01, *p* < 0.0001, respectively, and happy, *F*_2,6_ = 79.67, *p* < 0.0001 and *F*_2,6_ = 140.3, *p* < 0.0001, respectively, by decomposition were considerably significant. The AMG subdivision activation in response to sad was entirely right leaning. Happy elicited centromedial (CM) right leaning and certain emotion elicited laterobasal (LB) and superficial (SP) left leaning. All responses were right leaning for uncertain emotion and emotion identified at chance (*p* < 0.05; Fig. S5b; Tables S15, S16 and S18). Activation patterns were significant for AMG subdivisions by magnitude (Fig. 2c), with a prominent right hemispheric activation for certain emotion but little activation for emotion identified at chance (Fig. S6). Activation was not significant by hemispheric lateralization by subdivision (i.e., no difference between subdivision laterality). Deactivation patterns for sad and happy emotions by decomposition were significantly different, *F*_2,6_ = 3.51, *p* < 0.01 and *F*_2,6_ = 37.25, *p* < 0.0001, respectively (Fig. 2d; here note the difference between the last row for happy and sad, comparing deactivation with activation; in particular, the pattern for sad is matched in magnitude but appears changed in subdivision). The deactivation pattern for AMG subdivisions was significantly different for happy, *F*_2,6_ = 3.78, *p* < 0.01, but not for sad, *F*_2,6_ = 0.37, *p* = 0.69, with the magnitude of change being the contributor to the predominant left hemispheric lateralization for certain and uncertain emotion (Fig. S7; Tables S15, S16 and S18). Left hemispheric deactivations were noted for happy and sad uncertain emotion, but compared with certain emotion, they did not significantly change sidedness by hemisphere (Table S18). Follow-up testing for sad and happy found no differences in ROI by subdivision with a prominent left-leaning hemispheric lateralization to CM and LB for certain emotion and right-leaning lateralization to SP. Moreover, all left-leaning subdivisions were deactivated during uncertain emotion (Table S18). The change in lateralization as a factor of uncertain emotion is noted with differences by ROI, emotion (happy and sad), and hemisphere. A marked pattern of left hemisphere AMG deactivation for uncertain emotional stimuli was mirrored to activation of right hemisphere AMG emotional resolvability, where happy responses elicited the greatest AMG deactivation pattern coupled to emotional certainty. Even though sad emotion displayed significant deactivation, it was matched more consistently by activation. Therefore, it lacked definitive sidedness.

### Greater female over male AMG hemispheric lateralization to uncertain emotion

**Figure 3:**
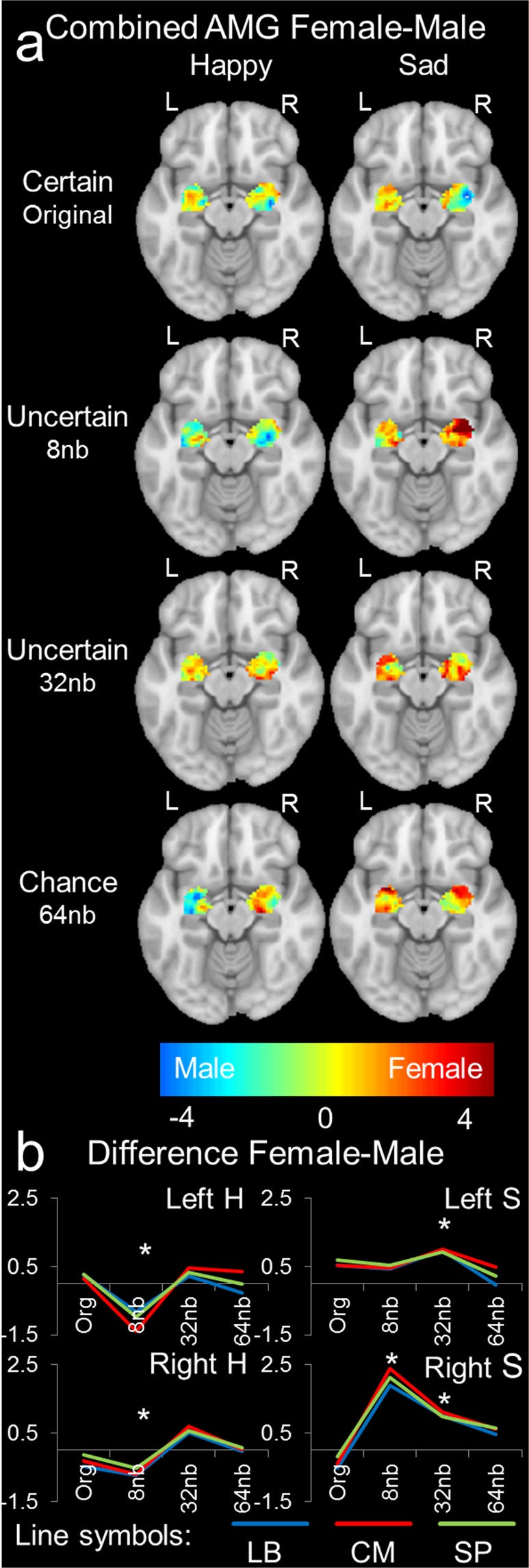
Sex difference in resolving uncertain emotion. Combined amygdala sex difference ([Female]-[Male]) for happy and sad emotion. (**a**) Average t-value volumes for [Female]-[Male] absolute difference for left and right hemisphere presentations by emotion resolvability (e.g., original, 8 nb, 32 nb, 64 nb; n = 12, 27.66 ± 3.47 years, range: 22 to 32 years; 6 females). Happy emotions found in the left column and sad emotions found in the right column with L and R indicating left and right hemisphere, respectively. (**b**) Average t-values for difference between [Female]-[Male] emotion resolvability for response by hemisphere (L – left, R – right) and emotion (H – happy, S – sad). Error bars represent standard error and asterisk represents significance (p < 0.01). Note the pattern for uncertain emotion (8 nb and 32 nb), while little difference for original emotion or emotion identified as chance is observed.

**Figure 4:**
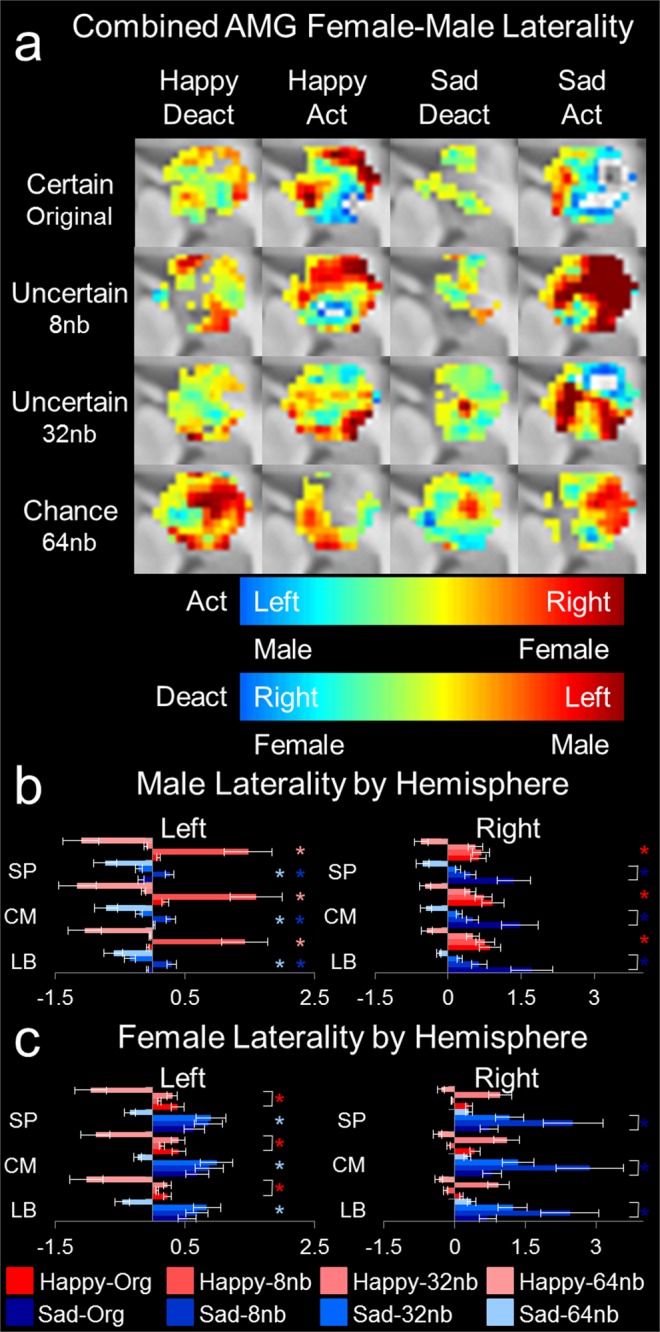
Activation and deactivation patterns for sex difference response to resolving emotion. The amygdala ROI presentation is identical to Fig. 2. (**a**) Combined amygdala absolute differences in voxel laterality by [Female]-[Male]. Activation and deactivation patterns for entire amygdala for bilateral presentations for emotional resolvability (e.g., original, 8 nb, 32 nb, 64 nb; n = 12 right-handed volunteers, age 27.66 ± 3.47 years, range: 22 to 32 years; 6 females). By column: happy deactivation, happy activation, sad deactivation, and sad activation. By row: certain stimuli in the un-altered original format, followed by uncertain 8 nb, 32 nb, and chance identifiable 64 nb. Color bars indicate activation and deactivation separately. For activation, male laterality is represented by left-side blue and female laterality is represented by right-side red. For deactivation, male laterality is represented by left-side red and female laterality is represented by right-side blue. (**b**) Bar plot of male laterality by hemisphere. (**c**) Bar plot of female laterality by hemisphere. Error bars represent standard error and asterisk represents significance (p < 0.01).

To gain insight into the lateralization process, we examined sex differences in a separate experiment (Fig. S1a) in response to emotional resolvability by AMG subdivision. We coded voxels as a function of sex by emotion, and the resultant AMG sex difference was most prevalent for differences in response to uncertain emotion. Certain emotion and chance emotion were not different between the sexes (Fig. 3). Generally, when an AMG subdivision was sexually dimorphic and lateralized by resolving uncertain emotion, adjacent subdivisions behaved in a similar manner (Fig. S9). For happy emotional resolvability, left and right AMG subdivisions were significantly different between the sexes, except the right superficial AMG (*F*_3,44_ = 2.789, *p* = 0.0516; Tables S19–S21; Fig. 3a left panel). Follow-up analysis found left (8 nb, t_12_ = 3.2323, p = 0.0080; Table S19; Fig. 3b, top left panel) and right (8 nb, t_12_ = 2.3812, *p* = 0.0364; Table S19; Fig. 3b, bottom left panel) AMG response to uncertain happy emotion significantly different between the sexes. For sad emotional resolvability, no sex difference existed for left AMG responses, while right AMG responses were significant by subdivision (Tables S22–S24; Fig. 3a right panel). Follow-up analysis found left (32 nb, t_12_ = 3.4637, p = 0.0053; Table S22; Fig. 3b, top right panel) and right (8 nb, t_12_ = 4.2249, *p* = 0.0014; 32 nb, t_12_ = 3.4637, p = 0.0053; Table S22; Fig. 3b, bottom right panel) AMG responses to uncertain sad emotion significantly different between the sexes. Sex differences in emotional resolvability were significant in binaurally presented stimuli; therefore, we explored the activation and deactivation patterns to determine the role of hemispheric lateralization. Voxels coded by hemispheric lateralization and segregated by activation and deactivation patterns were evaluated to ascertain sex differences in response to emotional resolvability (Fig. 4a). No pattern of activation or deactivation was significant for females (Table S25 and Fig. S10a), whereas activation (*F*_3,44_ = 6.359, p = 0.0011) and deactivation (*F*_3,44_ = 3.131, *p* = 0.0350) were significant for happy, but not sad, male emotional resolvability (Table S26 and Fig. S10b). There was a significant sex difference in response to the activation pattern for sad emotional resolvability (*F*_3,44_ = 3.403, *p* = 0.0257; Table S27 and Fig. S11a), with no difference in the deactivation pattern between the sexes (Fig. S11b).

Next, we evaluated hemispheric lateralization as a function of male and female AMG response differences. Male right AMG responded significantly to resolving uncertain emotion for happy, whereas the pattern for sad was only different from certain to chance identifiable emotion (Fig. 4b right panel; Tables S21 and S24). The left AMG of males responded significantly only to uncertain emotion for happy, and only the difference from certain to chance identifiable emotion for sad (Fig. 4b left panel). Female right AMG responded significantly to resolving uncertain emotion for happy, with no discernable difference for sad emotion (Fig. 4c right panel; Tables S21 and S24). The left AMG of females responded significantly to resolving sad, whereas only uncertain emotion was significantly different from certain emotion identification for happy (Fig. 4b left panel). The results indicate that uncertain emotion (8 nb and 32 nb) exhibited a significant difference between the sexes, a slight left/right difference for original emotion or emotion identified as chance, and manner of resolving emotion was different between the sexes. The present study provides evidence for differences in hemispheric lateralization between the sexes, with slight changes in activation and deactivation patterns contributing to sex differences in emotional resolvability.

### AMG is asymmetrically lateralized at rest, left leaning is less deactivated than right leaning

**Figure 5:**
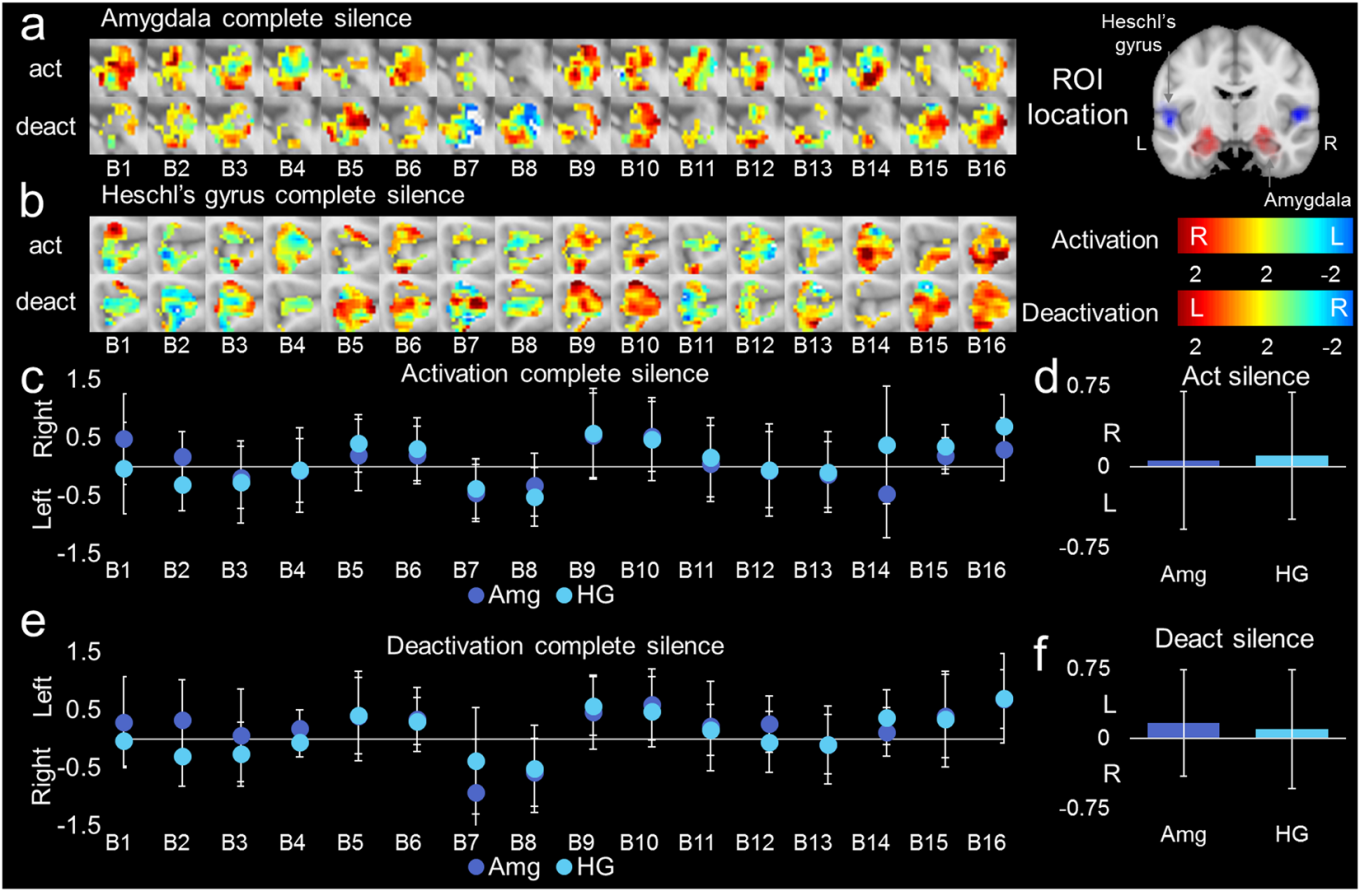
Sparse sampling of complete silence to ascertain baseline activation and deactivation patterns for entire ROI of HG and AMG (n = 15, 27.62 ± 3.21 years, range: 22 to 33 years; 6 females). (**a**) Amygdala and (**b**) Heschl’s gyrus complete silence activation (act-upper row) and deactivation (deact-bottom row) maps. (**c**) Activation complete silence averages from each map with left (i.e., negative values, meaning more left hemisphere activation) and right (i.e., positive values, meaning greater right hemisphere activation) hemispheric lateralization. (**d**) Average activation over series of blocks. (**e**) Deactivation complete silence averages from each map with left (i.e., positive values meaning more left hemisphere deactivation) and right (i.e., negative values meaning greater right hemisphere deactivation) hemispheric lateralization. (**f**) Average deactivation over series of blocks. The activation and deactivation in (**c**,**e**) were segregated into left and right hemisphere patterns to represent laterality. For (**c**,**e**), the “B” indicates the block number for cumulative averages separated by activation  or deactivation found in (**a**,**b**). No significant activation or deactivation during complete silence is observed in amygdala or Heschl’s gyrus. Error bars indicate standard error. For the activation bar, red represents right hemisphere activation and blue represents left hemisphere deactivation. For the deactivation bar, red represents left hemisphere activation and blue represents right hemisphere deactivation. Within the upper right brain insert, the ROI probability maps for the entire HG and AMG.

**Figure 6:**
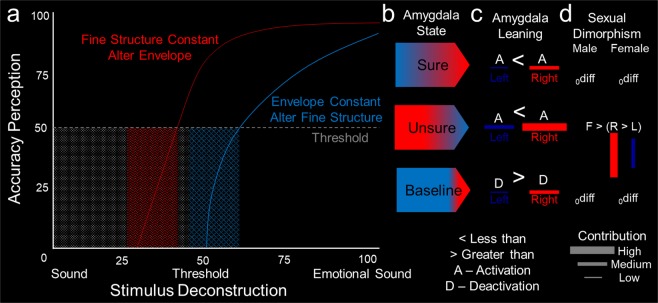
A unified mechanistic hemispheric lateralization model of AMG leaning to uncertain emotion. (**a**) Psychophysical model of FIS and ENV discrimination curves with threshold (dashed line). The curves indicate the change in accuracy of perception with emotion decomposition (i.e., stimulus deconstruction). Fine structure constant alter envelope indicates the emotion decompositions altering envelope. Envelope constant alter fine structure indicates the emotion decompositions altering fine structure. (**b**) Amygdala state color coded with right hemisphere (R) leaning colored in red and left hemisphere (L) leaning colored in blue. The three states (sure, unsure, baseline) pertain to different amygdala lateralizations. The proportion of color representing the dominant lateralization. (**c**) Amygdala leaning with left colored blue and right colored red, greater than sign “>” and less than sign “<”, and listed with the size of contribution represented by line thickness (bar thickness). Lastly, whether the amygdala is in a state of “A” (activation) or “D” (deactivation). (**d**) Amygdala sex differences with _0_diff representing negligible difference. The sure, unsure, and baseline state are consistent from left to right by row in (**b**–**d**).

Two negative control experiments were conducted (Fig. S1a) with voxels coded based on a model of stimuli that did not exist^75,86^ to assess the baseline resting state of the AMG and HG as a negative control for the previous experiments. Roving sparse sampling of silence did not modulate AMG (Fig. 5a) or HG (Fig. 5b), whether the volumes were acquired during complete silence or during interleaved trails (Figs S1 and S2 for methods; Figs S12 and S13). No modulation was observed for HG using the same experimental paradigm (Tables S28–S31). Although no modulation occurred temporally for the AMG, significant baseline hemispheric lateralization was found by time (Fig. S13d,e). The baseline asymmetry was found during complete silence (Table S32) and during interleaved silent trials (Tables S33–S35). When voxels were coded as a function of activation and deactivation patterns to ascertain left/right hemispheric lateralization, no significant difference was found (Fig. 6c,e, respectively). The average change of activation (Fig. 5d) or deactivation (Fig. 5f) for the AMG or HG was not significantly biased towards the left or right. Despite not encountering the contributing factor of left/right asymmetry, generally the AMG was significantly baseline-activated in a left lateralized pattern, with a right deactivation. The series of experiments provides evidence of baseline/rest AMG asymmetry, a negative control for the resolvability of uncertain emotional stimuli, and reveals a possible mechanism for discerning uncertain emotional perceptions. Here AMG leaning away from its normal leftward pattern and toward the right, as observed in response to uncertain emotion, was the dynamic process distinguishing the certainty of the affective state.

### A unified mechanistic hemispheric lateralization model of AMG leaning to uncertain emotion

We present a model for AMG resolvability of uncertain emotional stimuli. In the preceding experiments, we sought to identify the attributes of emotional sound at threshold^1,2,5–7,18,20^ to determine whether AMG functioning^10,41,44^, hemispheric lateralization^1,8,10,11,23,44,50–52^, subdivision differences^7,37,44^, or sex^10,11,50–53^ contributes to emotional resolvability. We propose that AMG chiefly responds to FIS cues of emotion (Fig. 6a). In Fig. 6b we couple FIS cues to AMG lateralization, where AMG laterality (L ≠ R) depends on emotional certainty, with right hemisphere laterality colored in red and left hemisphere laterality colored in blue. The model has baseline represented in blue which is predominantly right-deactivated but becomes completely right-activated when encountering unsure emotions. Certain emotions are right-hemisphere dominant, but less than uncertain emotions (Fig. 6b). Therefore, three basic emotional gradations exist: sure of the emotional information, unsure of the emotional information, and AMG operating at baseline hemispheric laterality. We propose transitional states exist, but based on the evidence in the present manuscript, these transitional states result in AMG responses resembling the sure, unsure, or baseline state (Fig. 6c). Sex differences in AMG leaning are predominant when emotional information is uncertain (Fig. 6d). Here the normal asymmetrical functional response of the AMG is exaggerated for females (greater right hemispheric laterality), and less for males (more left leaning). Previous studies may have not measured the AMG during uncertainty to elicit changes in laterality^10,41,44^, subdivision^7,37,44^, or sex^10,11,50–53^.

## Discussion

Here we examined how emotional stimuli are functionally resolved from uncertain emotional signals. Four main features of the results are of considerable interest. First, emotion in sound is primarily encoded in FIS, which results in significant AMG modulation when emotion is uncertain and no response when stimuli are chance identifiable. Chance emotion deactivates the left hemisphere AMG, while uncertain and certain emotional signals proportionally activate the right hemisphere AMG. Second, an AMG baseline cerebral dominance exists, which is left lateralized. Third, sex differences in response to emotion are greater during uncertain emotion. During resolving uncertain emotion, females respond with greater AMG right hemisphere activation, whereas males respond with more AMG left hemisphere activation. Fourth, evidence in the present study confirms that AMG is in a dynamic state. In summary, HG in both hemispheres was only responsive to the acoustic properties of sound, whereas AMG hemispheric left/right weighting was a dominant feature in determining emotional from non-emotional sound.

### Psychophysical curves reveal fine structure information essential for resolving emotion. 

Happy stimuli were identified primarily based on information contained in FIS, while sad stimuli utilized FIS, but relied more on ENV cues (Fig. S3). Psychophysically, the findings extend a number of recent reports^9,14,22,25,26,38,39,93^; for example, FIS importance for melody recognition and ENV for speech reception^26^. Although varying pitch and loudness (i.e., FIS and modulating ENV waveforms, respectively) have been thought essential for emotional detection in speech and music^18,25,38,39^, the exact contributions have remained elusive. The difference we observed between happy and sad emotion was that participants utilized less FIS information for sad music to determine the emotional attributes (i.e., approximately 3.5%), indicating that ENV cues were relevant for sad identification. Future studies should conduct emotional resolvability paradigms for speech aimed at threshold responses^9,14,22,27,34,38–41^, and differing emotional types^1,2,5,15,16,18,20^ based on differences in FIS and ENV contributions.

### Binaural emotion resolvability: The functional response to uncertain emotion is inversely coupled to psychophysics of stimuli certainty

Functional emotional identification has revealed differences between happy and sad musical recognition^15,16^, but the pattern of modulation to emotional certainty has remained unexplored. Functionally, HG was significantly active during all acoustic stimuli due to overlapping FIS and ENV information^29,32,33^, with prominent right hemispheric responses (Figs S6 and S8b). In contrast to psychophysical identification thresholds, sad music elicited slightly greater (albeit non-significant) auditory cortex activation compared with happy music (Figs S6 and S8a). The anterior aspect of the auditory cortex has been observed to functionally track the resolvability of pitch discrimination thresholds (i.e., Te1.2)^3,37^. Tracking pitch resolvability by using FIS is of high relevance for the auditory cortex^28–36^. The non-significant increase with decreasing emotional certainty (Fig. S6g,h) could possibly be attributed to stimuli relevance^1–3,5,18,19^. The magnitude of change in HG response to emotional stimuli was not as great as for AMG functioning, most likely due to the AMG role in emotional processing^1,4,7–9,23,42–45,48–52,93^.

The AMG response closely followed the certainty of both happy and sad emotion by decomposition. The response was significantly more modulated for happy stimuli (Fig. 2e), where lower initial activations (Fig. S6o,p) were coupled to greater balancing in activation and deactivation by emotional uncertainty (Figs 2b, S7 and S8). Although sad responses elicited greater AMG activation, this was not true psychophysically, as sad emotion utilized more ENV cues and elicited greater left hemisphere HG activation than happy emotion. The AMG role for sad music was consistent with strong AMG activation for negative valence^15,16,50,51^. Depending on the interpretation, the observation was either inconsistent from a discrete emotion perspective^1,2,5^, as emotionality was detected when stimuli varied in certainty, or consistent from the perspective of emotions as differentially activating specific regions, here based on salience^3–6,9,10,15,16,41,45,48^^,^. This is due to responses at the boundary of uncertainty/chance, where AMG modulation ceased (i.e., 64 nb; Fig. S6i,j). Initially right hemispheric functioning was greater for happy and sad emotions by a factor of three, but this analysis failed to balance for the contralateral hemisphere. The contralateral AMG hemisphere functioning (i.e., left ≠ right) contributed to suppression of sad stimuli, but not happy stimuli. This could be the principle reason for the psychophysical patterns observed for sad uncertain emotional stimuli being different than the functional patterns (Figs 2, S5 and S6).

### Hemispheric lateralized deactivation in response to uncertain emotion

Hemispheric lateralization in response to emotion is currently debated^1,8,11,23,40,41,44,51,52^. A previous meta-analysis failed to find right lateralization of emotion and limited valence-specific lateralization^8,40^. However, the contrary was found in a meta-analysis concerning prosody^40,41,44^, which may be attributed to the emotional stimuli under investigation, the lack of emotional range (as was explored here with band-wise decompositions), or to an improper separation of activation and deactivation patterns in the analysis. Separating activation and deactivation patterns by ROI freed the response to the contralateral hemisphere, thereby allowing the observation that right lateralized emotional modulation of HG and highly significant right lateralized modulation of AMG was based on the emotional certainty of the stimuli^11,15^. Here, left hemispheric deactivation to emotionally uncertain stimuli contributed mostly to prominent right hemispheric responses (Figs 2, S6 and S7)^41,44^. Previous reports have found left lateralized dominance of speech driven by segment length of linguistic features^17^, and left lateralization of the AMG associated with emotion^43^. Subsequent evidence indicates, differing contributions by different subdivisions (i.e., Te1.0 ≠ Te1.1 and Te1.1 ≠ Te1.2), possibly reflect a higher order right-lateralized FIS responding region in A1 (Fig. 2)^2,27,28,32–37^ based on frequency components to emotional salient stimuli^14,21,37^. Further, right-lateralized positive activation for the superficial AMG was previously linked to socio-affective musical reception^4,6,93^. The deactivation for AMG was significantly different, suggesting a contemporaneous activation and deactivation modulatory role for AMG subdivisions, but band-wise decompositions for sad stimuli were not significantly different (Figs 6b and S5b). The latter supports the observation of a minor difference in the psychophysical identification curve, possibly attributed to the flat negative (i.e., deactivation) response in the right hemisphere (Figs S5 and S6). Interestingly, happy stimuli decomposition responses in the AMG-ROI were significantly different from one another, possibly linked to the functional differences observed (Fig. S6)^15,23^. The results of AMG lateralization, by activation/deactivation, are bolstered by evidence provided by neuropsychological studies observing that AMG lesions lead to impaired perceptual encoding of affective stimuli^42^ (i.e., because here the contralateral hemispheric balancing is obliterated). Furthermore, impaired recognition and judgement of fear and sad emotions in music (sparring happy)^50,51^ contribute to the differences found between happy and sad emotions (Figs 2, 3 and 4). Overall, AMG functioning increased in activation when emotional stimuli increased in complexity, and ceased to activate when stimuli lost relevance in their fine structure cues, which encode emotion.

### Sex differences in resolving uncertain emotion exaggerate a slight baseline sex difference in perception

Females responding to uncertain emotion exploited greater right AMG functioning compared with males (Fig. 3). The present results indicate that uncertain emotion (8 nb and 32 nb) exhibits a significant difference between the sexes. A meta-analysis found that AMG activations were lateralized to the left with activations from both males and females represented equally in the AMG. However, a baseline moderate left-leaning bias was observed in both sexes^10^. The group concluded that sex differences in lateralization of AMG were not a robust finding in emotion, but they indicated that female-left and male-right lateralization in regions adjacent to the AMG were prominently lateralized^10^. In the present study, AMG subdivisions were sexually dimorphic and predominantly lateralized for uncertain emotion (Figs 3, 4 and S9). The present study found differences in left and right AMG responses to happy, but not to sad, emotions (Fig. 3b, top and bottom left panel, respectively). Here it was uncertain emotion that differed most between males and females. Previous studies analyzing sex differences between emotion were possibly not coding voxels appropriately for sexual dimorphism analyses and hemispheric lateralization effects (see SI methods). For example, activation and deactivation in the present study were significant for male happy emotional resolvability, but not for sad (Fig. S10b). Here a significant sex difference in response to the pattern of activation for sad emotion resolvability occurs (Fig. S11a), with no difference in the pattern of deactivation between the sexes (Fig. S11b). Similar to the meta-analysis^10^, it is possible that adjoining regions elicit a hemispheric spill-over effect to AMG subdivisions, thus canceling out when not emotionally relevant^10^. For the present analysis, separating activation and deactivation patterns by hemisphere, sex, and emotion type (original happy and sad or by certainty) has allowed contributions of voxel patterns to account for these previously unobserved differences. Future studies should analyze adjacent AMG and cortical structures for similar patterns^10,53^.

### AMG baseline modulatory state favors a significant right leaning deactivation

We report negative control experiments of sparse sampling silent stimuli to model the effects of the ROI at rest^86^. The AMG exhibited baseline hemispheric lateralization, but not HG. No modulation of the BOLD response as a function of the temporal effects of the block design occurred for HG or AMG. Utilizing the same model as the experiments resolving emotion serves as a control where any significant result attributed to the model for resolving emotion would nullify our results for a positive finding and bolster the results for a negative finding. No modulation was observed for emotional resolvability across blocks in our experimental design. However, a significant right asymmetric deactivation was noted for the AMG across silent blocks in complete silence or during interleaved silence. The AMG has been observed to maintain a long-term state to emotional stimuli, which is up-activated compared to baseline^64^. Furthermore, the right AMG has displayed more nonstationary behavior to neural responses, indicating that the right AMG is more variable than the left AMG^65^. While the present findings did not measure long-term habituation over silence to our stimuli, we observed a cyclical state of activation and deactivation similar to previous findings^64^ (Fig. 5), which was baseline lateralized (left less deactivated/right more deactivated) in complete silence and during interleaved silence. The authors of the report^64^ noted that valence decreased over 20 min and arousal ratings decreased over the 43 min of functional scanning. In the present experiment, no change was observed in valence to stimuli in psychophysical ratings or in fMRI responses (see SI Results). Whether emotional resolvability varies as a function of time with equally valent but different stimuli should be investigated.

### A unified mechanistic hemispheric lateralization model of AMG leaning to uncertain emotion

For clarity in communicating our results, we describe a model of AMG hemispheric lateralization (Fig. 6) with theoretical discrimination curves for FIS and ENV coupled to perception with sure, unsure, and baseline states. Combining the emotion psychophysical-fMRI model with decision making^1^ and electrophysiological findings^7^ to determine if emotion driven hemispheric lateralization is limited to humans or primates would be of high interest (i.e. and the contributions of language specialization), as previous authors have argued it is a human feature^53^. Extending the range^23^ and subtype of emotional gradations^20^ would determine if there are any restrictions across emotional states.

### Distinguishing uncertain emotions will contribute to the neurobiological basis of ‘emotion’

We report that uncertain emotion leads to patterned activations/deactivations of hemispheric lateralization depending on certainty. The importance of this finding is that AMG deactivates to cues when no longer relevant, but persists to increase in activation as cues become emotionally complex. The AMG leans away from its leftward baseline hemispheric lateralized state (and more towards the right) the more complex the cue becomes. Current investigations into emotion detection exploring what constitutes an ‘emotion’ reveal the difficulty^20^ of expressing the richness behind the intricate processes involved in decision making^1^, psychophysical cues^26,37–39^, and neurobiological underpinnings^4–9,13–15,17,43–45,48^ in emotional resolvability. Future studies should explore the dynamic state of the AMG during emotion selection^1,2,6,20^, differentiating hemispheric specializations for emotion subtypes^7–10,27,44^, or sub-define the fine structure cues which differentiate noise from emotional sound. The present results reveal the threshold of an emotional sound and provide an understanding of how humans determine with different neural responses, emotional from non-emotional sound stimuli.

### Conclusions and future directions

Our results indicate emotion identification in music at threshold utilizes fine structure cues. Fine structure is a prominent cue used to help identify prosodic emotion in speech (i.e., pitch/fine structure)^14,22,26,38^. Our results indicate AMG activation was modulated by emotion identification and was absent when emotional stimuli were chance identifiable, especially in the left hemisphere. The HG was activated, but did not vary to emotionally uncertain stimuli. Furthermore, the right hemisphere AMG was considerably more deactivated in response to uncertain emotion in music. The right hemisphere AMG has been linked to socio-affective musical reception^4,6,93^, emotional certainty of the stimuli^11,15^, and limited valence-specific lateralization^8,40^^,^. Future experiments could attempt to isolate left versus right hemispheric AMG functioning. The AMG role in emotional processing is well known^1,4,7–9,23,42–45,48–52,93^, therefore, future studies should decipher different emotionally relevant states such as threshold emotion (i.e. in the present study) or confusing emotional states. Here we present evidence the AMG functionally followed uncertain auditory signals in music with a pronounced left hemisphere deactivation. Future studies should investigate AMG functioning during different emotional states.

## Supplementary information

Supplementary Information

## Data Availability

All data is uploaded to the Open Science Framework: Manno, Francis A. M. 2018. “Music Psychophysics.” OSF. November 20. https://osf.io/8ws7a.
