# Supplementary material for: The human amygdala disconnecting from auditory cortex preferentially discriminates musical sound of uncertain emotion by altering hemispheric weighting
Source: Sci Rep. 2019 Oct 15;9:14787. doi: 10.1038/s41598-019-50042-1 (PMC6794305; doi:10.1038/s41598-019-50042-1)
Supplement: Supplementary file 1 — Supplementary Information [file 41598_2019_50042_MOESM1_ESM.docx]

# Supplementary Information: The human amygdala disconnecting from auditory cortex preferentially discriminates musical sound of uncertain emotion by altering hemispheric weighting

Francis AM Manno III^1,2^**^†^**, Condon Lau^2^**^†^**, Juan Fernandez-Ruiz^3^, Sinaí Hernandez-Cortes Manno^4^, Shuk Han Cheng^4^, Fernando A. Barrios^5^**^†^**

**Author Affiliations**:

^1^School of Biomedical Engineering, Faculty of Engineering, The University of Sydney, Sydney, New South Wales, Australia

^2^Department of Physics, City University of Hong Kong, HKSAR, China

^3^Departamento de Fisiología, Facultad de Medicina, Universidad Nacional Autónoma de México, México City, 04510 México.

^4^Department of Biomedical Sciences, City University of Hong Kong, HKSAR, China

^5^Instituto de Neurobiología, Universidad Nacional Autónoma de México, Juriquilla, Querétaro, México

Correspondence and requests for materials should be addressed to F.A.M.M. (email: Francis.Manno@Sydney.edu.au) or C.L. (email: condon.lau@cityu.edu.hk) or F.A.B. (email: fbarrios@unam.mx)

Contents

[Supplementary Information: The human amygdala disconnecting from auditory cortex preferentially discriminates musical sound of uncertain emotion by altering hemispheric weighting 1](#_Toc13385617)

[Contents 1](#_Toc13385618)

[1. Introduction 5](#_Toc13385619)

[1.1. Stimuli utilized for emotional resolvability (Finished) 10](#_Toc13385620)

[2. Results detailed and elaborated (For peer review only) 12](#_Toc13385621)

[2.1. Psychophysics of emotion identification 12](#_Toc13385622)

[2.1.1. Contrasting happy and sad fine structure stimuli 18](#_Toc13385623)

[2.1.2. Happy fine structure 18](#_Toc13385624)

[2.1.3. Sad fine structure 19](#_Toc13385625)

[2.2. The functional response of emotion resolvability 19](#_Toc13385626)

[2.2.1. Heschl’s gyrus does not cease modulation to emotionally uncertain stimuli, but follows FIS cues 23](#_Toc13385627)

[2.2.2. Amygdala functional response follows identification of uncertain emotional stimuli, deactivating at chance emotional identification. 27](#_Toc13385628)

[2.2.3. Deactivation of right hemispheric AMG lateralization of emotional response by emotional uncertainty. 30](#_Toc13385629)

[2.2.4. Contralateral hemispheric HG activation balances lateralization to uncertain emotion; deactivation of left AMG hemispheric functioning follows uncertain stimuli 38](#_Toc13385630)

[2.3. Amygdala sexual dimorphism in emotional resolvability by hemispheric lateralization 40](#_Toc13385631)

[2.3.1. Happy absolute [Female] – [Male] sexual dimorphism 41](#_Toc13385632)

[2.3.2. Sad absolute [Female] – [Male] sexual dimorphism 42](#_Toc13385633)

[2.3.3. Absolute [Female] – [Male] sexual dimorphism for activation and deactivation profiles 48](#_Toc13385634)

[3. Silent analysis negative control experiments of baseline and phantom sparse sampling 52](#_Toc13385635)

[3.1. Heschl’s gyrus silent analysis during complete silence and interleaved phantom sparse sampling 54](#_Toc13385636)

[3.2. Amygdala gyrus silent analysis during complete silence and interleaved phantom sparse sampling 58](#_Toc13385637)

[4. The effect size of emotion 64](#_Toc13385638)

[4.1. Effect sizes for emotion and power analysis 64](#_Toc13385639)

[5. References 71](#_Toc13385640)

**Figures**

Fig. S1 | Experimental design, sparse sampling paradigm parameters utilized to optimize, run format, analysis of explanatory variables 6

Fig. S2 | Silent stimuli used for sparse sampling 7

Fig. S3 | Finalized sparse sampling design 8

Fig. S4 | Example stimuli 9

Fig. S5 | Psychophysics of emotion discrimination 12

Fig. S6 | Functional resolvability of uncertain emotional sound 21

Fig. S7 | Difference mapping of hemispheric lateralization resolvability of uncertain emotion 23

Fig. S8 | Zoom of ROI subdivisions 37

Fig. S9 | Absolute difference [Female] – [Male] by amygdala subdivisions (bilateral presentation) 50

Fig. S10 | Activation and deactivation profiles for entire amygdala in female and male separately (bilateral presentation) 51

Fig. S11 | [Female] – [Male] average response happy and sad by amygdala activation and deactivation (bilateral presentation) 52

Fig. S12 | Heschl’s gyrus complete silence and interleaved phantom sparse sampling 54

Fig. S13 | Amygdala complete silence and interleaved phantom sparse sampling 59

Fig. S14 | Power analysis based on effect size from literature 65

**Tables**

Table S1 | Summary statistics of original emotion identification 5

Table S2 | Acoustic stimuli and their respective decompositions 11

Table S3 | Happy envelope (ENV) decompositions 13

Table S4 | Happy fine structure (FIS) decompositions 13

Table S5 | Sad envelope (ENV) decompositions 14

Table S6 | Sad fine structure (FIS) decompositions 14

Table S7 | Sad fine structure averages by stimuli (sad-FIS) 14

Table S8 | Happy fine structure averages by stimuli (Hap-FIS) 15

Table S9 | Average happy percent identification for all stimuli categories 16

Table S10 | Average sad percent identification for all stimuli categories 16

Table S11 | Representative stimuli 01 sad percent identification 17

Table S12 | Representative stimuli 03 happy percent identification 17

Table S13 | Average HG right and left hemisphere activation for sad stimuli 25

Table S14 | Average HG right and left hemisphere activation for happy stimuli 26

Table S15 | Average amygdala right and left hemisphere activation for sad stimuli 28

Table S16 | Average amygdala right and left hemisphere activation for happy stimuli 29

Table S17 | Hemispheric lateralization of functional emotional modulation 34

Table S18 | Approximate hemispheric lateralization of functional emotional modulation 35

Table S19 | [Female] – [Male] Averages Happy 42

Table S20 | [Female] – [Male] predominance sided average for Happy 43

Table S21 | Average Female and Male amygdala happy responses 44

Table S22 | [Female] – [Male] Averages Sad 45

Table S23 | [Female] – [Male] predominance sided average for Sad 46

Table S24 | Average Female and Male amygdala sad responses 46

Table S25 | [Female] average happy and sad by activation and deactivation (bilateral presentation) 48

Table S26 | [Male] average happy and sad by activation and deactivation (bilateral presentation) 49

Table S27 | [Female] – [Male] average happy and sad by activation and deactivation (bilateral presentation) with sided predominance 49

Table S28 | Heschl’s gyrus negative control experiments: Complete silence 55

Table S29 | Heschl’s gyrus negative control experiments: Interleaved phantom sparse sampling average by sequence in time 56

Table S30 | Heschl’s gyrus negative control experiments: Interleaved phantom sparse sampling by block of stimuli (Block 1 sequences) 57

Table S31 | Heschl’s gyrus negative control experiments: Interleaved phantom sparse sampling by block of stimuli (Block 2 sequences) 58

Table S32 | Amygdala negative control experiments: Complete silence 60

Table S33 | Amygdala negative control experiments: Interleaved phantom sparse sampling average by sequence in time 61

Table S34 | Amygdala negative control experiments: Interleaved phantom sparse by block of stimuli (Block 1 sequences) 62

Table S35 | Amygdala negative control experiments: Interleaved phantom sparse by block of stimuli (Block 2 sequences) 63

Table S36 | Studies for determining effect size statistics of emotion 67

**Equations**

Equation S1 | ROI Modulation sum of absolute t-value differences 8

Equation S2 | Percent identification sum of absolute change 8

Equation S3 | Weighted modulation for figure 5 calculations 8

Equation S4 | Cohen’s *d* calculation for effect size 65

Equation S5 | The σ for effect size determination 65

# Introduction

Little is known concerning the neural responses involved with resolving emotional information at the threshold of experience. Where does emotion *bind* to its basic components of sound to derive meaning (Treisman, 1996; Treisman, 1999)? Where does sound become emotional sound? The ability to distinguish emotions based on specific neural activations in humans will empower research into determining what constitutes an ‘emotion’ from basic non-emotional sound counterparts (Pfeifer, 1998). The present study investigated the functional modulation to certain and uncertain emotional stimuli. No previous study has addressed the functional neural components underlying threshold emotional experience and the ability to resolve uncertain emotion. In the present experiments the subtlety of emotion is of interest. A psychophysics experiment with uncertain emotional stimuli was conducted to determine the threshold of emotional identification. The psychophysics experiment was utilized to identify stimuli corresponding to certain, uncertain, and chance identifiable emotional stimuli. Stimuli were then utilized during an optimized auditory sparse sampling fMRI experiment in order to assess the specific modulatory properties of the auditory cortex and amygdala to resolving uncertain emotion. Lastly hemispheric lateralization calculations and sexual dimorphism voxel analyses were factored by certain and uncertain emotional stimuli to determine the significance of sidedness and change of functional response to uncertain emotion. Lastly, the verification aspect of the experiment utilized silent periods during sparse sampling in order to ascertain the functional baseline modulation of the amygdala and HG. Additionally, effect size statistics and power analysis were calculated from the literature and compared to effects sizes from the study to ascertain if the emotional effect was in line with previous literature. See Fig. S1 | Experimental design, sparse sampling paradigm parameters utilized to optimize, run format, analysis of explanatory variables.

Table S1 | Summary statistics of original emotion identification

|  | Stimuli Correct | Variance | SD | 95%CI |
| --- | --- | --- | --- | --- |
| Total out of 32 stimuli | 28.54 | 3.63 | 1.91 | 0.74 |
| Percentage out of 32 stimuli (%) | 89.21 | 0.35 | 5.95 | 1.25 |

Table. Average percent stimuli identification for the entire set of Peretz Emotional Identification Task stimuli (Dalla Bella et al., 2001a; Dalla Bella et al., 2001b; Peretz et al., 2013; Peretz et al., 1998a; Peretz et al., 1998b). Percentage and number correct in stimuli correct column, followed by variance SD- standard deviation, and 95% confidence intervals.

Fig. S1 | Experimental design, sparse sampling paradigm parameters utilized to optimize, run format, analysis of explanatory variables


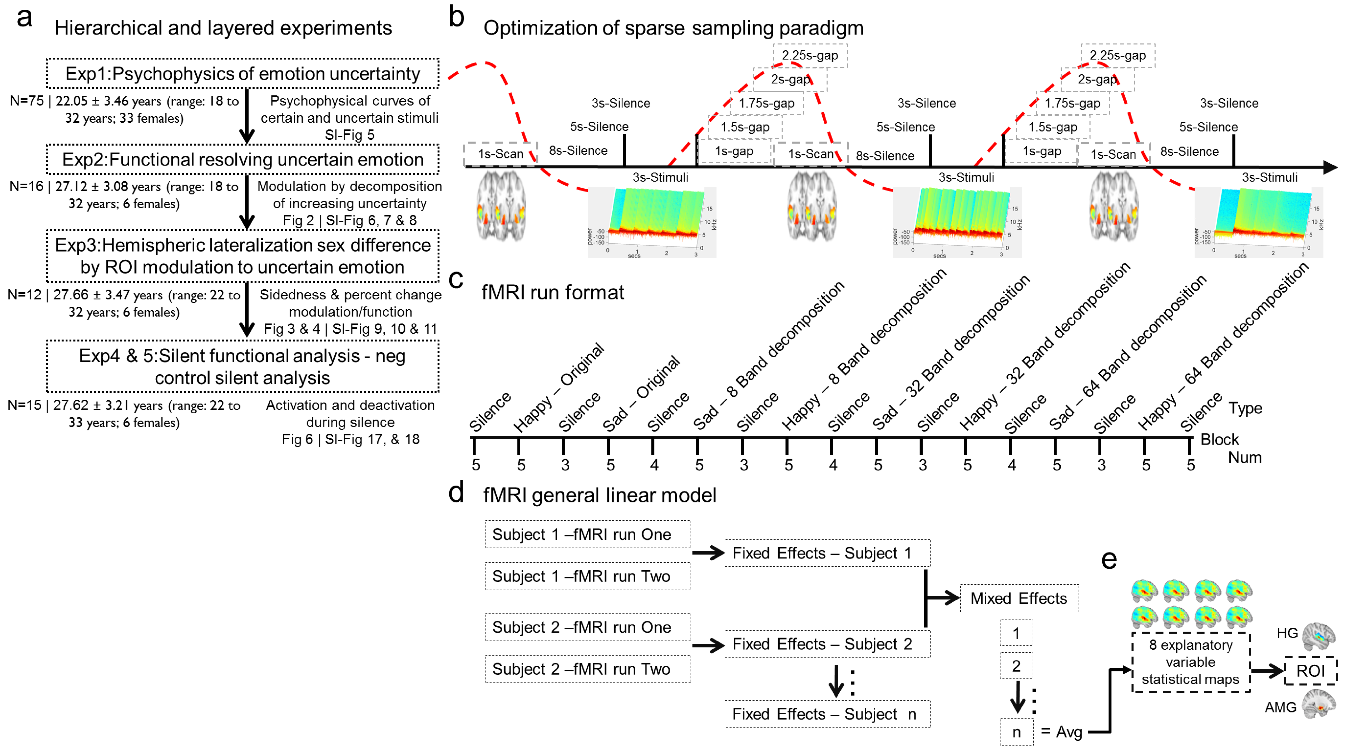


**Figure.** (a) Flow of the experiments: 1) psychophysics of emotion discrimination (n=75) to determine psychophysical profiles of certain, uncertain and chance stimuli, 2) fMRI evaluation of certain, uncertain and chance stimuli (n=16) to assess the modulation of emotion by decomposition and increasing emotional certainty, 3) hemispheric lateralization analysis (N=12) to determine the sidedness to uncertain emotion, and 4) and 5) negative control experiments - sparse sampling silence. Within (a) the experiments in the present manuscript are hierarchical – we propose research hypothesis and test a theory before proceeding to a subsequent experiment, and are layered, we ask several questions within an experiment. (b) Representative sparse sampling paradigm parameters evaluated. Across the arrow from left-to-right, time represented along the presentation of three stimuli blocks. Stimuli are represented by fast Fourier transform waveforms in blue, with TA represented by a representative MNI 152-T1 standard below the time-arrow, and the canonical HRF as a red dashed line. The figure is a representation of parameters as described for optimizing auditory functioning. For example, we originally choose an 8s-silent period to proceed our stimuli, but this was changed to 6s and 4s during different protocols. The gap preceding the TA was changed from 1s to 1.5s, 1.75s, 2s and 2.25s to capture the most robust response of the HRF. These silent periods during the stimuli-presentation block were altered to: 1) capture the most robust HRF signal and 2) to decrease the entire block time length (i.e. TR), to the minimal value necessary in order to increase the number of stimuli presentations. Final sparse sampling format was depicted in manuscript Fig. 1. (d) Run format and analysis of explanatory variables. The fMRI-run format describes the run format for stimuli presentations. (e) The fixed effects to average run one and run two from one individual. These averages are then combined in a mixed effects analysis for n = x individuals depending on which fMRI experiment. The 8 explanatory variables (i.e., original happy, happy-8nb, happy-32nb, happy-64nb, original sad, sad-8nb, sad-32nb, and sad-64nb), averaged from the mixed effects analysis of all the individuals were utilized to derive whole head statistical maps, from which specific ROI were derived.

Fig. S2 | Silent stimuli used for sparse sampling

**Figure.** Silent stimuli delivered during sparse sampling devoid of fine structure and envelope information. The spectrogram has time on the y-axis and normalized frequency on the x-axis. Note no frequency by amplitude variations exist.

Equation S1 | ROI Modulation sum of absolute t-value differences

$$ROIModultion\left( t-value \right)=\sum\frac{\left| Org-8nb \right|\text{ + }\left| 8nb-32nb \right|\text{ + }\left| 32nb-64nb \right|}{\left| Org \right|}$$

Equation S2 | Percent identification sum of absolute change

$$Percentidentification\left( \text{\%} \right)=\sum\frac{\left| Org-8nb \right|\text{ + }\left| 8nb-32nb \right|\text{ + }\left| 32nb-64nb \right|}{\left| Org \right|}$$

Equation S3 | Weighted modulation for figure 5 calculations

$$Change=\frac{ROIModultion\left( t-value \right)}{Percentidentification\left( \text{\%} \right)}$$

Fig. S3 | Finalized sparse sampling design


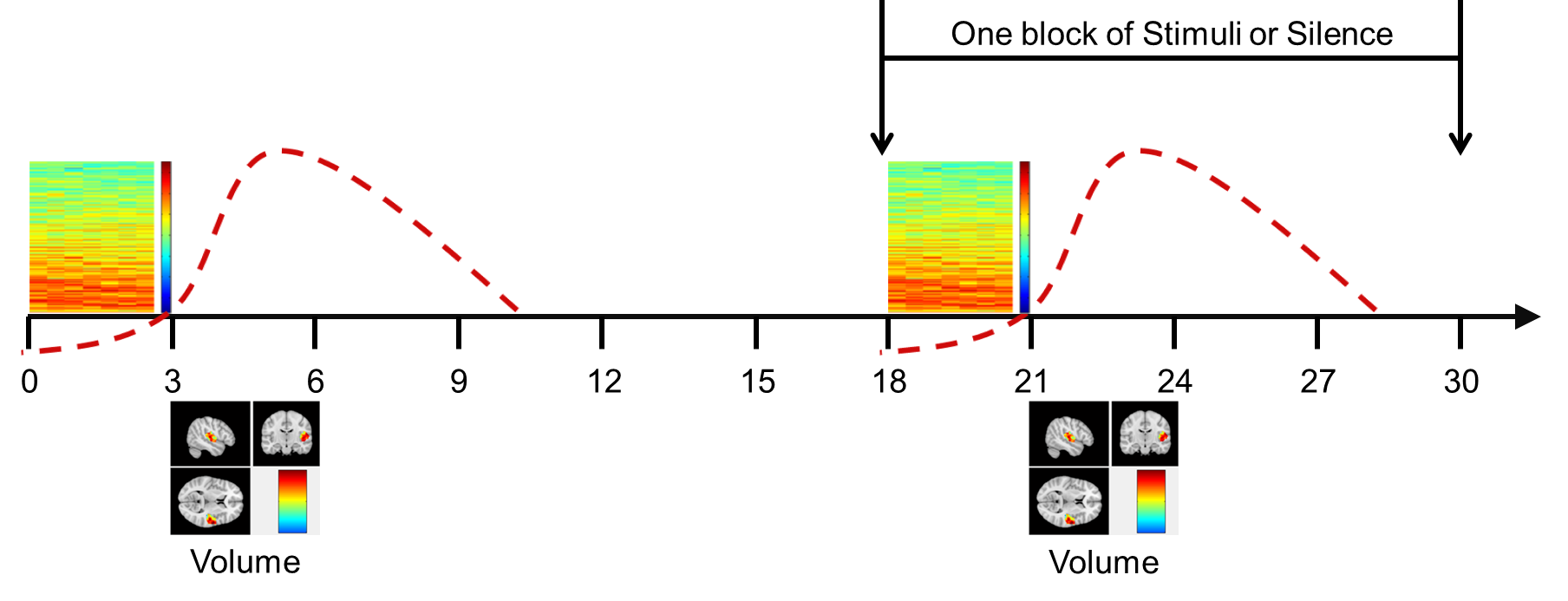


**Figure.** Sparse sampling protocol and representative stimuli. Sparse sampling paradigm with seconds on the x-axis and representative stimuli spectrogram. A symbolic HRF above and below the axis representative volume acquisitions.

Fig. S4 | Example stimuli


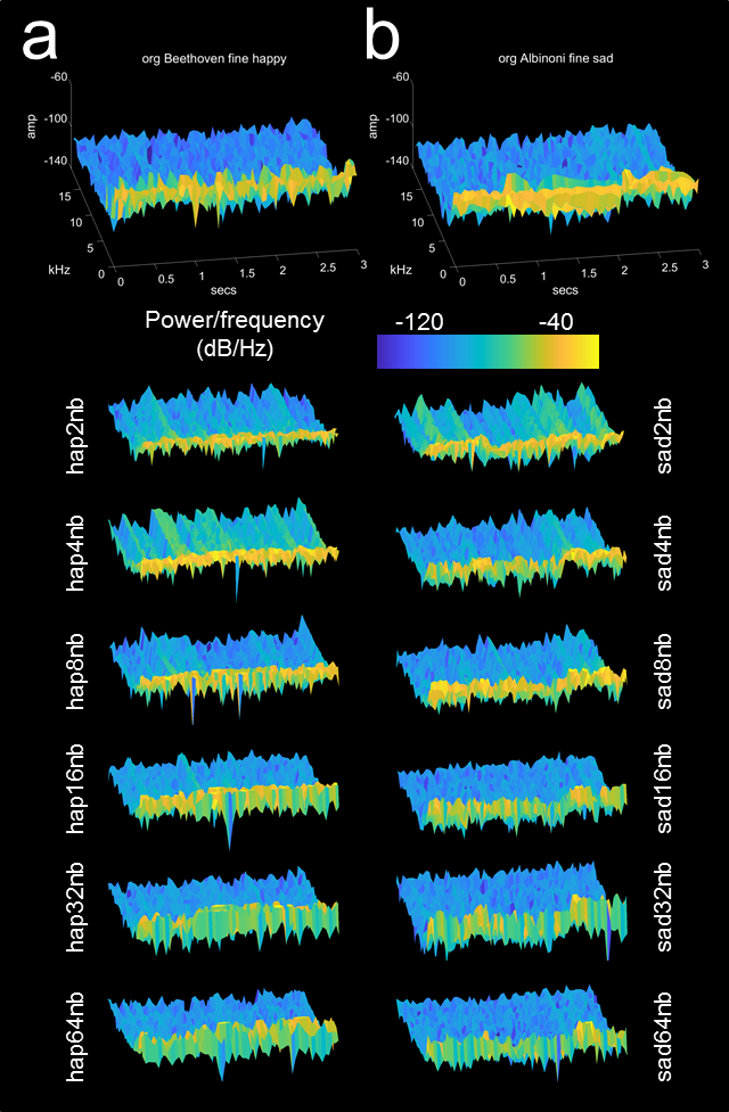


**Figure**. Representative emotional stimuli (Table S2). Spectrogram for Beethoven (a) and Albinoni excerpts (b), classified as Fine-Happy and Fine-Sad, respectively. The spectrogram was normalized by all stimuli representations from 0 to 20kHz normalized frequency units (colormap from -40 red color to -120; SI Methods).

## Stimuli utilized for emotional resolvability (Finished)

Instrumental excerpts of classical piano known to evoke categorically a sense of sadness or happiness (Dalla Bella, et al, 2001; Fritz, et al., 2009) were taken from a previous study (Peretz et al., 1998, 2001; [www.brams.umontreal.ca/peretz](http://www.brams.umontreal.ca/peretz)). The complete repertoire of 32 musical excerpts was utilized in addition to 224 chimerized excerpts.

Table S2 | Acoustic stimuli and their respective decompositions

| Composer | Work | Measure(a) | M.M.(b) | Key | Instrumentation | Emotion | num. stim | Decomposition |
| --- | --- | --- | --- | --- | --- | --- | --- | --- |
| Beethoven | Piano Concerto no. 4 (3rd mvt) | 191–200(2) | 150 | G Maj | Piano & orchestra | happy | 3 | 03.wav_01.wav |
| Beethoven | Piano Concerto no. 4 (3rd mvt) | 439–452(2) | 150 | G Maj | Piano & orchestra | happy | 11 | 11.wav_02.wav |
| Beethoven | Symphony no. 3 (3rd mvt) | 38–56 | 180b | F Maj | Orchestra | happy | 26 | 26.wav_04.wav |
| Beethoven | Symphony no. 6 (3rd mvt) | 9(3)–16(1) | 240 | D Maj | Orchestra | happy | 17 | 17.wav_05.wav |
| Haendel | Utrecht's Te Deum | 5–14(1) | 112 | D Maj | Orchestra | happy | 6 | 06.wav_23.wav |
| Mozart | Die Zauberflöte (Act 1 no. 2 Papageno's Aria) | 18(2)–24(2) | 80 | G Maj | Orchestra | happy | 25 | 25.wav_27.wav |
| Mozart | Eine kleine nachtmusik (1st mvt) | 5(3)–10(3) | 154 | G Maj | String orchestra | happy | 28 | 28.wav_19.wav |
| Mozart | Piano Concerto no. 23 (3rd mvt) | 1–8 | 255 | A Maj | Piano | happy | 31 | 31.wav_10.wav |
| Mozart | Piano Concerto no. 27 (3rd mvt) | 1–8 | 167 | B flat Maj | Piano & orchestra | happy | 18 | 18.wav_16.wav |
| Ravel | Tombeau de Couperin (Rigaudon) | Bar1–9(2) | 100 | C Maj | Piano | happy | 24 | 24.wav_22.wav |
| Saint-Saëns | Carnaval des Animaux (Finale) | 10–26(4) | 220 | C Maj | Piano & orchestra | happy | 7 | 07.wav_30.wav |
| Saint-Saëns | Carnaval des Animaux (La volière) | 1–9(2) | 88 | F Maj | Piano & orchestra | happy | 15 | 15.wav_08.wav |
| Schumann | Kinderszenen (Op 15 no. 9) | 1–9 | 240 | C Maj | Piano | happy | 14 | 14.wav_20.wav |
| Verdi | La Traviatta (Brindisi) | 1–15(1) | 100 | B flat Maj | Orchestra | happy | 29 | 29.wav_12.wav |
| Verdi | Rigoletto (Act 1 no. 4) | 69–73 | 150 | C Maj | Orchestra | happy | 21 | 21.wav_09.wav |
| Vivaldi | L'Autunno (1st mvt) | 1(2)–4(3) | 126 | F Maj | Orchestra | happy | 32 | 32.wav_13.wav |
| Albinoni | Adagio | 7–14(1) | 48 | G min | Orchestra | sad | 1 | 03.wav_01.wav |
| Bach | Passionsmusik nach dem evangelisten Matthäus | 1–5(2) | 67 | E min | Orchestra | sad | 2 | 11.wav_02.wav |
| Brahms | Piano Concerto no. 1 (2nd mvt) | 21(3)–24(1) | 48 | D Maj | Piano & orchestra | sad | 4 | 26.wav_04.wav |
| Bruch | Kol Nidrei | 9–11(1) | 20 | D min | Double bass & organ | sad | 5 | 17.wav_05.wav |
| Chopin | Nocturne Op 27 no. 1 | 2(2)–6(3) | 72 | C sharp min | Piano | sad | 23 | 06.wav_23.wav |
| Chopin | Nocture Op 48 no. 1 | 1–4(1) | 52 | C min | Piano | sad | 27 | 25.wav_27.wav |
| Chopin | Nocturne Op 9 no. 1 | 0–4(1) | 100 | B flat min | Piano | sad | 19 | 28.wav_19.wav |
| Debussy | Prélude: Des pas sur la Neige | 4–8(1) | 35 | D min | Piano | sad | 10 | 31.wav_10.wav |
| Grieg | Peer Gynt's Suite no. 2 (Solveigs lied) | 13(4)–17(3) | 69 | A min | Orchestra | sad | 16 | 18.wav_16.wav |
| Mahler | Symphony no. 5 (3rd mvt) | 12(4)–16(3) | 54 | A min | Orchestra | sad | 22 | 24.wav_22.wav |
| Mozart | Piano Concerto no. 23 (2nd mvt) | 1–3 | 35 | F sharp min | Piano | sad | 30 | 07.wav_30.wav |
| Rachmaninov | Piano Concerto no. 2 (2nd mvt) | 13(2)–17 | 48 | E Maj | Piano & orchestra | sad | 8 | 15.wav_08.wav |
| Ravel | Concerto in G (2nd mvt) | 1–4(2) | 38 | E Maj | Piano & orchestra | sad | 20 | 14.wav_20.wav |
| Rodrigo | Concerto de Aranjuez (Adagio) | 1–4(4) | 40 | B min | Guitar & orchestra | sad | 12 | 29.wav_12.wav |
| Saint-Saëns | Carnaval des Animaux (Le cygne) | 1–5 | 55 | G Maj | Piano & cello | sad | 9 | 21.wav_09.wav |
| Schubert | String Quartet no. 14 (2nd mvt) | 1–4 | 72 | G min | String quartet | sad | 13 | 32.wav_13.wav |

(a)=The number in parentheses indicates beat number in the measure. (b)=This value corresponds to a half note. Table Adopted from Peretz et al., 1998.

# Results detailed and elaborated

## Psychophysics of emotion identification

Psychophysical testing revealed a significant trend for FIS encoded happy or sad stimuli by decomposition as a factor of stimuli uncertainty. Fig. S5 | Psychophysics of emotion discrimination (a) left and (b) right side panel demonstrate happy and sad responses, respectively, based on the percent identification with the original excerpt. Happy stimuli were modulated by correct identification for the majority of decompositions in FIS. Sad stimuli followed a similar FIS identification pattern, but certainty for all sad decompositions was more sloping (Fig. S5 | Psychophysics of emotion discrimination (a)). Therefore, although sad emotion identification utilizes FIS, it was ENV which aids in the identification for sad stimuli. Overall, we observed decreasing emotional certainty by decomposition, associated with FIS cues for both happy and sad stimuli. We wondered how the psychophysical profile of emotional certainty translates into a functional response as measured by fMRI?

Fig. S5 | Psychophysics of emotion discrimination


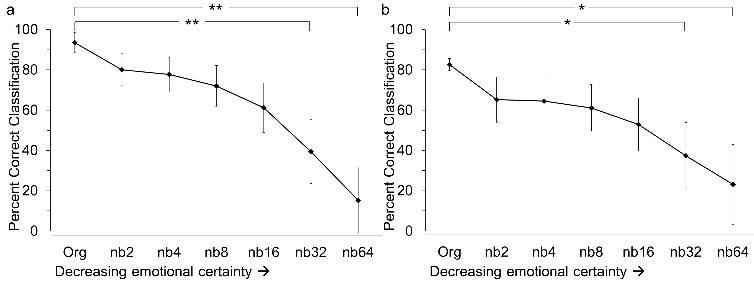


**Figure**. Percent correct identification of happy and sad emotional stimuli by fine structure with decreasing emotional certainty. (A) Happy stimuli in black/fine structure and sad stimuli in grey/envelope. (B) Sad stimuli in black/fine structure and happy stimuli in grey/envelope. Asterisk * adjusted t-test *p* < 0.001, and double asterisk ** indicates ANOVA *p* < 0.001. Error bars represent confidence interval (n=75; Table S3 | Happy envelope (ENV) decompositions, Table S4 | Happy fine structure (FIS) decompositions, Table S5 | Sad envelope (ENV) decompositions, Table S6 | Sad fine structure (FIS) decompositions).

Table S3 | Happy envelope (ENV) decompositions

| Stim (BWD) | Happy-ENV avg | Variance | SD | CI |
| --- | --- | --- | --- | --- |
| Org Sad | 0.8400 | 0.1181 | 0.3281 | 0.0000 |
| nb2 | 0.3325 | 0.1888 | 0.4296 | 0.1710 |
| nb4 | 0.3400 | 0.1898 | 0.4311 | 0.1705 |
| nb8 | 0.3733 | 0.1763 | 0.4042 | 0.1617 |
| nb16 | 0.4558 | 0.1826 | 0.4174 | 0.1578 |
| nb32 | 0.6092 | 0.1602 | 0.3899 | 0.1241 |
| nb64 | 0.7508 | 0.1338 | 0.3551 | 0.0939 |

Table S4 | Happy fine structure (FIS) decompositions

| Stim (BWD) | Happy-FIS avg | Variance | SD | CI |
| --- | --- | --- | --- | --- |
| Org Happy | 0.9442 | 0.0376 | 0.1334 | 0.0000 |
| nb2 | 0.8108 | 0.1243 | 0.3289 | 0.0782 |
| nb4 | 0.7875 | 0.1360 | 0.3538 | 0.0855 |
| nb8 | 0.7317 | 0.1714 | 0.4055 | 0.0995 |
| nb16 | 0.6250 | 0.2078 | 0.4529 | 0.1206 |
| nb32 | 0.4100 | 0.2205 | 0.4666 | 0.1577 |
| nb64 | 0.1700 | 0.1348 | 0.3585 | 0.2101 |

Table S5 | Sad envelope (ENV) decompositions

| Stim (BWD) | Sad-ENV avg | Variance | SD | CI |
| --- | --- | --- | --- | --- |
| Org Happy | 0.9442 | 0.0376 | 0.1334 | 0.0000 |
| nb2 | 0.1892 | 0.1243 | 0.3289 | 0.2001 |
| nb4 | 0.2125 | 0.1360 | 0.3538 | 0.2807 |
| nb8 | 0.2683 | 0.1714 | 0.4055 | 0.1849 |
| nb16 | 0.3750 | 0.2078 | 0.4529 | 0.1638 |
| nb32 | 0.5900 | 0.2205 | 0.4666 | 0.1271 |
| nb64 | 0.8292 | 0.1352 | 0.3590 | 0.0800 |

Table S6 | Sad fine structure (FIS) decompositions

| Stim (BWD) | Sad-FIS avg | Variance | SD | CI |
| --- | --- | --- | --- | --- |
| Org Sad | 0.8400 | 0.1181 | 0.3281 | 0.0000 |
| nb2 | 0.6675 | 0.1888 | 0.4296 | 0.1122 |
| nb4 | 0.6600 | 0.1898 | 0.4311 | 0.1136 |
| nb8 | 0.6267 | 0.1763 | 0.4042 | 0.1152 |
| nb16 | 0.5442 | 0.1826 | 0.4174 | 0.1306 |
| nb32 | 0.3908 | 0.1602 | 0.3899 | 0.1640 |
| nb64 | 0.2492 | 0.1338 | 0.3551 | 0.1968 |

Table S7 | Sad fine structure averages by stimuli (sad-FIS)

|  | SadOrg | Sad2nb | Sad4nb | Sad8nb | Sad16nb | Sad32nb | Sad64nb |
| --- | --- | --- | --- | --- | --- | --- | --- |
| Stim01 | 0.8000 | 0.7600 | 0.8400 | 0.7600 | 0.8000 | 0.6933 | 0.1600 |
| Stim02 | 0.8400 | 0.7200 | 0.6933 | 0.7067 | 0.5333 | 0.2400 | 0.1600 |
| Stim04 | 0.5733 | 0.8267 | 0.9067 | 0.9067 | 0.8400 | 0.5200 | 0.1333 |
| Stim05 | 0.9200 | 0.8400 | 0.8133 | 0.7867 | 0.6533 | 0.4000 | 0.1867 |
| Stim08 | 0.8933 | 0.3333 | 0.2667 | 0.4267 | 0.3600 | 0.3600 | 0.2400 |
| Stim09 | 0.4933 | 0.9600 | 0.9600 | 0.9067 | 0.6933 | 0.5600 | 0.4133 |
| Stim10 | 0.7467 | 0.9200 | 0.9467 | 0.9200 | 0.8933 | 0.4933 | 0.0800 |
| Stim12 | 0.8133 | 0.4667 | 0.5467 | 0.4400 | 0.4133 | 0.2800 | 0.1600 |
| Stim13 | 0.9600 | 0.9600 | 0.8933 | 0.8400 | 0.7467 | 0.6133 | 0.1333 |
| Stim16 | 0.9067 | 0.8933 | 0.8933 | 0.8933 | 0.7733 | 0.5733 | 0.1467 |
| Stim19 | 0.8000 | 0.9600 | 0.9200 | 0.6400 | 0.4267 | 0.3733 | 0.2533 |
| Stim20 | 0.9600 | 0.8000 | 0.6667 | 0.6533 | 0.6133 | 0.3733 | 0.2667 |
| Stim22 | 0.9333 | 0.7733 | 0.6400 | 0.5067 | 0.4800 | 0.3467 | 0.1200 |
| Stim23 | 0.8800 | 0.8933 | 0.7600 | 0.6000 | 0.3467 | 0.2000 | 0.0400 |
| Stim27 | 0.9600 | 0.8667 | 0.8800 | 0.8267 | 0.7333 | 0.4400 | 0.1867 |
| Stim30 | 0.9600 | 1.0000 | 0.9733 | 0.8933 | 0.6933 | 0.0933 | 0.0400 |

Table S8 | Happy fine structure averages by stimuli (Hap-FIS)

|  | Hap-Org | Hap2nb | Hap4nb | Hap8nb | Hap16nb | Hap32nb | Hap64nb |
| --- | --- | --- | --- | --- | --- | --- | --- |
| Stim31 | 0.9867 | 0.8667 | 0.8667 | 0.7867 | 0.2933 | 0.2133 | 0.0667 |
| Stim32 | 1.0000 | 0.7333 | 0.5600 | 0.4533 | 0.5200 | 0.4667 | 0.1333 |
| Stim03 | 0.9333 | 0.6667 | 0.3867 | 0.4933 | 0.2267 | 0.1200 | 0.0400 |
| Stim14 | 1.0000 | 0.3867 | 0.5333 | 0.3467 | 0.4133 | 0.2133 | 0.1467 |
| Stim15 | 0.4800 | 0.9200 | 0.8933 | 0.9867 | 0.9600 | 0.9467 | 0.9067 |
| Stim17 | 1.0000 | 0.6133 | 0.6800 | 0.4400 | 0.3867 | 0.1467 | 0.0800 |
| Stim18 | 1.0000 | 0.6933 | 0.7067 | 0.5600 | 0.4000 | 0.2133 | 0.2400 |
| Stim06 | 0.9733 | 0.7867 | 0.6800 | 0.7867 | 0.6400 | 0.5333 | 0.3600 |
| Stim07 | 1.0000 | 0.8267 | 0.8000 | 0.6533 | 0.4000 | 0.0667 | 0.1067 |
| Stim11 | 0.9333 | 0.8800 | 0.8933 | 0.9333 | 0.8667 | 0.7067 | 0.4000 |
| Stim28 | 0.9867 | 0.7200 | 0.8133 | 0.8933 | 0.9067 | 0.6000 | 0.2800 |
| Stim 29 | 0.8667 | 0.7867 | 0.8133 | 0.9467 | 0.9200 | 0.8667 | 0.6933 |
| Stim21 | 0.9867 | 0.2667 | 0.2933 | 0.2800 | 0.2400 | 0.1067 | 0.0933 |
| Stim24 | 0.9733 | 0.6800 | 0.7467 | 0.8000 | 0.7867 | 0.6400 | 0.2267 |
| Stim25 | 0.9867 | 0.4533 | 0.5867 | 0.4400 | 0.5600 | 0.3333 | 0.1067 |
| Stim26 | 1.0000 | 0.4000 | 0.3067 | 0.2267 | 0.1867 | 0.0800 | 0.1067 |

Table S9 | Average happy percent identification for all stimuli categories

|  | HapOrg | Hap2nb | Hap4nb | Hap8nb | Hap16nb | Hap32nb | Hap64nb |
| --- | --- | --- | --- | --- | --- | --- | --- |
| Avg | 0.9442 | 0.8108 | 0.7875 | 0.7317 | 0.6250 | 0.4100 | 0.1700 |
| SD | 0.1289 | 0.1809 | 0.1882 | 0.1703 | 0.1768 | 0.1613 | 0.0927 |
| AvgChange | 0.1333 | 0.0233 | 0.0558 | 0.1067 | 0.2150 | 0.2400 | 0.1700 |

**Table.** Avg - the mean for all stimuli in question; SD - for the category standard deviation; AvgChange - the average change between categories.

Table S10 | Average sad percent identification for all stimuli categories

|  | SadOrg | Sad2nb | Sad4nb | Sad8nb | Sad16nb | Sad32nb | Sad64nb |
| --- | --- | --- | --- | --- | --- | --- | --- |
| Avg | 0.8400 | 0.6675 | 0.6600 | 0.6267 | 0.5442 | 0.3908 | 0.2492 |
| SD | 0.1380 | 0.1950 | 0.1990 | 0.2529 | 0.2691 | 0.2921 | 0.2423 |
| AvgChange | 0.1725 | 0.0075 | 0.0333 | 0.0825 | 0.1533 | 0.1417 | 0.2492 |

**Table.** Avg - the mean for all stimuli in question; SD - for the category standard deviation; AvgChange - the average change between categories.

Table S11 | Representative stimuli 01 sad percent identification

|  | FineAVG | Variance | SD | CI |
| --- | --- | --- | --- | --- |
| Stim01 | 0.8000 | 0.1622 | 0.4027 | 0.0896 |
| Stim01-ENV+Stim03-FIS-2nb | 0.7600 | 0.1849 | 0.4300 | 0.0982 |
| Stim01-ENV+Stim03-FIS-4nb | 0.8400 | 0.1362 | 0.3691 | 0.0801 |
| Stim01-ENV+Stim03-FIS-8nb | 0.7600 | 0.1849 | 0.4300 | 0.0982 |
| Stim01-ENV+Stim03-FIS-16nb | 0.8000 | 0.1622 | 0.4027 | 0.0896 |
| Stim01-ENV+Stim03-FIS-32nb | 0.6933 | 0.2155 | 0.4642 | 0.1115 |
| Stim01-ENV+Stim03-FIS-64nb | 0.1600 | 0.1362 | 0.3691 | 0.1967 |

**Table.** Albinoni excerpt from the orchestral Adagio in G min categorized as sad (stimuli 01).

Table S12 | Representative stimuli 03 happy percent identification

|  | FineAVG | Variance | SD | CI |
| --- | --- | --- | --- | --- |
| Stim03 | 0.9333 | 0.0631 | 0.2511 | 0.0517 |
| Stim03-ENV+Stim01-FIS-2nb | 0.6667 | 0.2252 | 0.4746 | 0.1167 |
| Stim03-ENV+Stim01-FIS-4nb | 0.3867 | 0.2404 | 0.4903 | 0.1611 |
| Stim03-ENV+Stim01-FIS-8nb | 0.4933 | 0.2533 | 0.5033 | 0.1446 |
| Stim03-ENV+Stim01-FIS-16nb | 0.2267 | 0.1777 | 0.4215 | 0.1869 |
| Stim03-ENV+Stim01-FIS-32nb | 0.1200 | 0.1070 | 0.3271 | 0.2079 |
| Stim03-ENV+Stim01-FIS-64nb | 0.0400 | 0.0389 | 0.1973 | 0.3139 |

**Table.** Beethoven excerpt from the Piano Concerto no. 4 (3rd mvt) categorized as happy (stimuli 03).

### Contrasting happy and sad fine structure stimuli

The mean responses for the original happy and sad stimuli were significantly different (F1,6 = 5.66, p < 0.001). The mean difference between happy/sad categories was 7.15% ± 6.99% percent in identification. Contrasting happy and sad stimuli revealed several stimuli were significantly different as a factor of changing emotional certainty, but most significant for happy versus sad by happy-64nb (F2,15 = 18.34, p < 0.001). The 64nb was considered chance, whereas 32nb and 8nb were considered uncertain based on their identification with the original excerpts.

Contrasting happy and sad stimuli reveled the original categories were significantly different from the band decompositions happy/sad-sad2nb *F*_(2,15)_ = 6.626, *p* = 0.01, happy/sad-sad4nb *F*_(2,15)_ = 10.47, *p* = 0.02, happy/sad-happy32nb *F*_(2,15)_ = 5.25, *p* = 0.021, and happy/sad-happy64nb *F*_(2,15)_ = 18.34, *p* < 0.001 with nonsignificant observations for happy/sad-sad8nb *F*_(2,15)_ = 2.77, *p* = 0.1, happy/sad-sad16nb *F*_(2,15)_ = 1.47, *p* = 0.27, happy/sad-sad32nb *F*_(2,15)_ = 0.91, *p* = 0.43, happy/sad-sad64nb *F*_(2,15)_ = 2.34, *p* = 0.14, happy/sad-happy2nb *F*_(2,15)_ = 1.77, *p* = 0.21, happy/sad-happy4nb *F*_(2,15)_ = 1.01, *p* = 0.39, happy/sad-happy8nb *F*_(2,15)_ = 2.28, *p* = 0.14, and happy/sad-happy16nb *F*_(2,15)_ = 3.01, *p* = 0.08. Differences within a category of happy or sad stimuli where then investigated to determine the trend of decomposition compared with the original excerpts. Due to the difference in psychophysical emotional identification, it was of interest to determine whether a difference occurred between original excerpts and decompositions based on emotion.

### Happy fine structure

Identifications determined psychophysically reveled the original happy excerpt was significantly different from happy-32nb *F*_(5,10)_ = 4.10, *p* = 0.028, and happy-64nb *F*_(5,10)_ = 12.97, *p* < 0.001, however the identifications for the decompositions for happy-2nb *F*_(5,10)_ = 0.80, *p* = 0.58, happy-4nb *F*_(5,10)_ = 0.45, *p* = 0.80, happy-8nb *F*_(5,10)_ = 2.17, *p* = 0.14, happy-16nb *F*_(5,10)_ = 1.92, *p* = 0.18 were not significantly different. Follow-up testing found the original happy excerpt was significantly different from decompositions for happy-32nb *t*_(15)_ = 10.67, *p* <0.0001, and happy-64nb *t*_(15)_ = 17.64, *p* < 0.0001. The change from the original happy excerpt was most considerable for the nb16 and nb32.

### Sad fine structure

Identifications determined psychophysically reveled the original sad excerpt was not significantly different from the other decompositions when correcting for multiple comparisons in the ANOVA, sad-2nb *F*_(5,10)_ = 3.65, *p* = 0.11, sad-4nb *F*_(5,10)_ = 3.16, *p* = 0.14, sad-8nb *F*_(5,10)_ = 3.62, *p* = 0.11, sad-16nb *F*_(5,10)_ = 1.75, *p* = 0.31, sad-32nb *F*_(5,10)_ = 0.38, *p* = 0.91, and sad-64nb *F*_(5,10)_ = 1.12, *p* = 0.50. Nevertheless, utilizing a post-hoc paired t-test and comparing the 32nb and 64nb decompositions to the original sad excerpt, as was observed for happy stimuli, revealed decomposition for sad stimuli were significantly different for sad-32nb *t*_(15)_ = 6.25, *p* < 0.0001, and sad-64nb *t*_(15)_ = 9.74, *p* < 0.0001. The change of classification from the original sad excerpt was most considerable for the original excerpt to the nb2 stimuli (i.e., the first decomposition).

## The functional response of emotion resolvability

An ANOVA found HG modulation by stimuli decomposition significantly different *F*_(3,15)_ = 20.29, *p* < 0.001, but follow-up testing failed to confirm differences as a function of uncertain or chance emotion eliciting different responses from certain emotion. Further, little difference persisted in percent change from original, for left and right hemispheres responding to happy or sad uncertain emotion for HG (Fig. S6 | Functional resolvability of uncertain emotional sound, (g) and (h); Fig. S7 | Difference mapping of hemispheric lateralization resolvability of uncertain emotion, (a) for HG; Fig. S8 | Zoom of ROI for percent changes; Table S13 | Average HG right and left hemisphere activation for sad stimuli and Table S14 | Average HG right and left hemisphere activation for happy stimuli). Here the change in sound information of FIS or ENV cues most likely elicited the change in HG activation. The pattern of HG modulation was a slight increase in activation with decreasing certainty (albeit, no deactivation as observed for AMG), followed by uncertain stimuli eliciting a slight decrease in activation (Fig. S7 | Difference mapping of hemispheric lateralization resolvability of uncertain emotion, (a)), with right hemispheric prominent responses to happy and sad emotion.

Amygdala functional response closely followed identification of uncertain emotional stimuli, deactivating at chance emotional identification. When emotional stimuli where increasingly uncertain, amygdala functioning (Fig. S6 | Functional resolvability of uncertain emotional sound; ROI – (i) through (l)) significantly increased (from the original to 32nb stimuli), for happy or sad emotions (Fig. S6 | Functional resolvability of uncertain emotional sound, (e) and (f) compared with (m) and (n)). The observed trend, following uncertain emotional identification, ceased when stimuli were identified by chance (i.e. unable to discriminate happy or sad emotion as indicated for 64nb stimuli, for both hemispheres; Fig. S6 | Functional resolvability of uncertain emotional sound (o) and (p); last row for each stimuli set and last bar graph set). No significant differences were observed between amygdala subdivisions based on emotion resolvability (*p* < 0.05); nevertheless, significant differences were found by decomposition and hemisphere to happy and sad emotion. The functional changes for AMG were considerably greater than for HG (Table S15 | Average amygdala right and left hemisphere activation for sad stimuli and Table S16 | Average amygdala right and left hemisphere activation for happy stimuli).

For the amygdala, right hemisphere functioning was greater by a factor of three in activation over left hemisphere functioning for sad and happy stimuli. Follow-up testing to investigate the cessation in modulation due to the deactivation observed at chance identifiable 64nb stimuli (Fig. S6 | Functional resolvability of uncertain emotional sound, (o) and (p)), revealed highly significant differences from the original excerpts for: sad emotion by right hemisphere response to 64nb (*t*_3_ = 4.58, *p* < 0.001), sad emotion left hemisphere response to 64nb (*t*_3_ = 10.74, *p* < 0.001), happy emotion right hemisphere response to 64nb (*t*_3_ = 6.63, *p* < 0.01), and happy emotion left hemisphere response to 64nb (*t*_3_ = 5.47, *p* < 0.001; Fig. 4). The AMG modulation was a significant increase with decreasing emotional certainty by decomposition; interestingly, at the level of chance (i.e. 64nb), stimuli lead to significant deactivation of AMG in both hemispheres for happy and sad emotions (Fig. S7 | Difference mapping of hemispheric lateralization resolvability of uncertain emotion). The greatest deactivation was found in the left hemisphere across the stimuli decompositions, an average right -0.4267 versus left -1.4089 deactivation for chance emotion (Fig. S7 | Difference mapping of hemispheric lateralization resolvability of uncertain emotion).

Fig. S6 | Functional resolvability of uncertain emotional sound


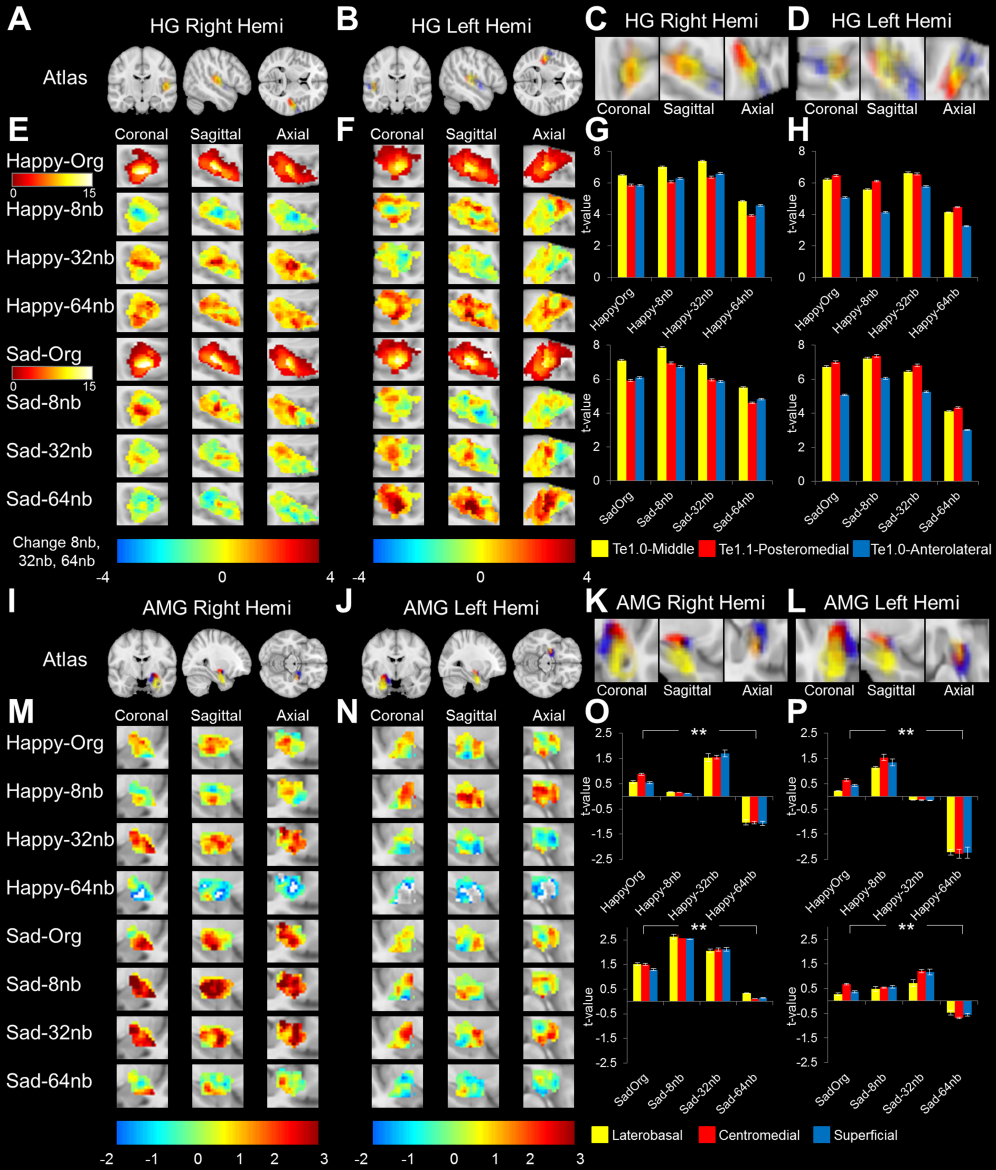


**Figure**. Emotional certainty functionally modulates AMG utilizing fine structure cues while HG responds to sound content (n=16). The panel was segmented by ROI delineation, divided by hemisphere (Hemi) with an activation map, and a bar graph representing mean volumetric t-value of ROI. (A) HG Right Hemi, (B) HG Left Hemi, (I) AMG Right Hemi and (J) AMG Left Hemi are hemispheric ROI depictions by coronal, sagittal, and axial section. (C) HG Right Hemi, (D) HG Left Hemi, (K) AMG Right Hemi and (L) AMG Left Hemi are detailed depictions of the probability map delineations seen in panel (A), (B), (I), and (J), respectively. (E), (F), (M) and (N) are right and left hemispheric depictions of the coronal, sagittal and axial section activation map t-values for HG and AMG ROI. The entire HG activation range was depicted for original Happy and Sad (E) and (F) from 0 to 15 with mean 6.16 ± 3.69 t-value range. A difference activation map was constructed for 8nb, 32nb, and 64nb to better represent the original stimuli change by decomposition, due to the small difference. The difference figures were normalized across conditions with stimuli directly comparable, blue colors (i.e., -4) representing a deactivation from original and red colors (ie., 4) representing activation. For AMG, (M) and (N) activation maps for the stimuli with the colorbar from -2 to 3, the former representing a decrease t-value and the latter representing an increase t-value. Activation maps for AMG were normalized across all stimuli conditions and therefore all figures were directly comparable representations. For HG, (G) and (H), and for AMG, (O) and (P), a bar plot of the average volumetric activation t values from each ROI with decompositions on the x-axis and t-value activation on the y-axis (** *p* < 0.001; ANOVA). Colors for HG: yellow-TE1.0, red-TE1.1 and blue-TE1.2. Colors for AMG: yellow-laterobasal, red-centromedial and blue-superficial.

Fig. S7 | Difference mapping of hemispheric lateralization resolvability of uncertain emotion


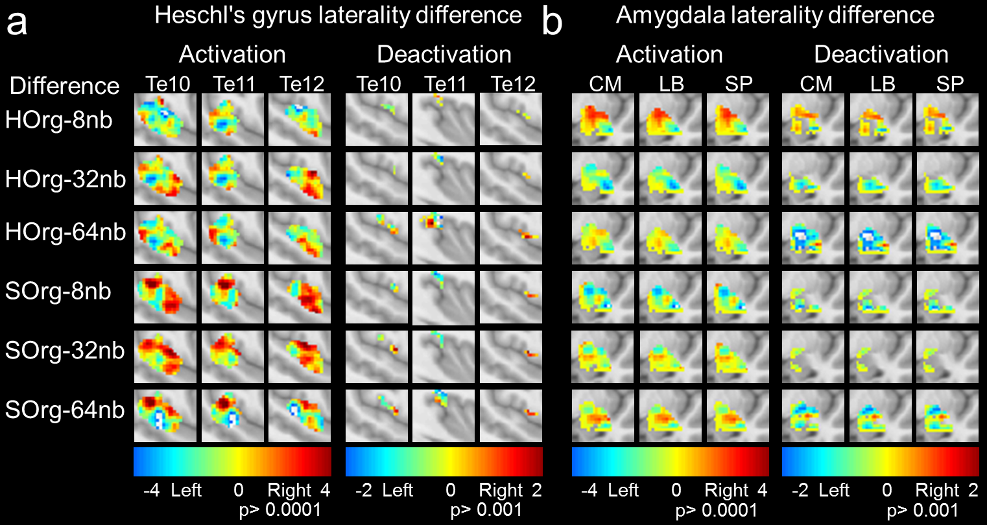


**Figure.** Difference mapping of hemispheric lateralization resolving uncertain emotion. (a) and (b)) Difference maps of original stimuli were created as a factor of descending decomposition for Org-8nb, Org-32nb, and Org-64nb for H-happy and S-sad located on the y-axis, separated in the range of activation and deactivation for each subdivision for HG and AMG.

### Heschl’s gyrus does not cease modulation to emotionally uncertain stimuli, but follows FIS cues

The difference between HG ROI subdivisions was analyzed to determine whether the response by decomposition to emotion identification was significant by subdivision, hemisphere, emotion, or a combination.

For the right and left hemisphere response to sad emotion, no significant difference was found by subdivision HG-TE1.0/HG-Te1.1/HG-Te1.2, *F*_(2,6)_ = 1.348, *p* = 0.2625 and *F*_(2,6)_ = 3.149, *p* = 0.0453, respectively. However, the left hemisphere HG response to sad emotion by decomposition was significant *F*_(2,6)_ = 6.439, *p* = 0.0004, while the right hemisphere HG response was not significant *F*_(2,6)_ = 2.014, *p* = 0.1137. For the right and left hemisphere HG response to happy emotion, no significant difference was found by subdivision HG-TE1.0/HG-Te1.1/HG-Te1.2, *F*_(2,6)_ = 0.6797, *p* = 0.5081 and *F*_(2,6)_ = 2.752, *p* = 0.0665, respectively. However, the right and left hemisphere HG response to happy emotion by decomposition was significant *F*_(2,6)_ = 3.743, *p* = 0.0121 and *F*_(2,6)_ = 4.438, *p* = 0.0049, respectively.

For HG-ROI sad right and sad left hemisphere, the average t-value activation difference by decomposition was 1.01 ± 0.48 and 1.52 ± 0.55 which translated into a percent difference change from the response to the original excerpt of 15.82% ± 12.14% and 24.1% ± 14.10%, respectively. For HG-ROI happy right and left hemisphere, the average t-value activation difference by band was 1.01 ± 0.19 and 1.53 ± 0.27 which translated into a percent change response from original excerpt of 16.70% ± 0.52% and 25.90% ± 8.75%, respectively.

An ANOVA found the HG modulation by stimuli significantly different *F*_(3,15)_ = 20.29, *p* < 0.001. Follow-up testing to investigate the decrease in modulation observed due to the response to uncertain emotional stimuli at 64nb revealed right hemisphere sad ≠ 64nb *t*_(3)_ = 4.786, *p* < 0.01 and right hemisphere happy ≠ 64nb, *t*_(3)_ = 3.758, *p* = 0.016 were highly statistically significant were as left hemisphere sad ≠ 64nb *t*_(3)_ = 2.6492, *p* = 0.039, left hemisphere happy ≠ 64nb *t*_(3)_ = 2.6355, *p* = 0.038, were significant at p < 0.05.

The correlation over the band decomposition was an asymptotic increase at first with a modest monotonic reversal caused the response to uncertain emotional stimuli at 64nb with happy-Right Hemisphere was r^2^=0.55, happy-Left Hemisphere was r^2^= 0.51, sad-Right Hemisphere was r^2^= 0.59, and sad-Left Hemisphere was r^2^=0.63. The change of response along the functional modulation was greatest for left and right hemisphere for happy or sad stimuli, from the original to 64nb stimuli.

Table S13 | Average HG right and left hemisphere activation for sad stimuli

| Subnuclei | Right  TE1.0 ± SD | Right  TE1.1 ± SD | Right  TE1.2 ± SD | Left  TE1.0 ± SD | Left  TE1.1 ± SD | Left  TE1.2 ± SD |
| --- | --- | --- | --- | --- | --- | --- |
| SadOrg | 7.0891±3.97 | 5.9196±4.36 | 6.0794±3.62 | 6.7268±4.08 | 7.0039±4.08 | 5.0658±3.51 |
| Sad-8nb | 7.8489±4.72 | 6.9489±4.87 | 6.7267±4.45 | 7.2111±4.40 | 7.3665±4.43 | 6.0606±3.88 |
| Sad-32nb | 6.8546±4.09 | 5.9645±4.23 | 5.8654±3.87 | 6.4479±3.69 | 6.8143±3.76 | 5.2648±2.99 |
| Sad-64nb | 5.4869±4.01 | 4.5953±3.97 | 4.8186±3.82 | 4.1104±3.07 | 4.3215±3.05 | 3.0246±2.71 |
| Average | 6.8199±4.20 | 5.8571±4.36 | 5.8725±3.94 | 6.1241±3.81 | 6.3766±3.83 | 4.8540±3.27 |
| Hemisphere | - | - | - | - | - | - |
| ANOVA Row | *F*=2.721, *p*=0.0475 | *F*=2.525, *p*=0.0608 | *F*=2.593, *p*=0.0559 | *F*=2.721, *p*=0.0475 | *F*=2.525, *p*=0.0608 | *F*=2.593, *p*=0.0559 |
| ANOVA Col | *F*=0.9542, *p*=0.3306 | *F*=0.5076, *p*=0.4776 | *F*=2.502, *p*=0.1163 | *F*=0.9542, *p*=0.3306 | *F*=0.5076, *p*=0.4776 | *F*=2.502, *p*=0.1163 |

*No follow-up performed because all subdivisions were non-significant.

#### Sad differences for left versus right hemisphere

Sad right Te1.0 was not significantly different than left Te1.0 *F*_1,3_ = 0.9542, *p* = 0.3306, with stimuli significantly different by decomposition *F*_1,3_ = 2.721, *p* = 0.0475. Sad right Te1.1 was not significantly different than left Te1.1 *F*_1,3_ = 0.5076, *p* = 0.4776, with stimuli not significantly different by decomposition *F*_1,3_ = 2.525, *p* < 0.0608. Sad right Te1.2 was not significantly different than left Te1.2 *F*_1,3_ = 2.502, *p* = 0.1163, with stimuli not significantly different by decomposition *F*_1,3_ = 2.593, *p* = 0.0559. When accounting for comparisons by ROI (i.e. column) by decomposition (i.e. row), there were no differences between left versus right hemisphere for sad stimuli. Nevertheless, this comparison was not accounting for the differences between hemisphere, so follow-up statistics are performed for hemispheric lateralization.

Table S14 | Average HG right and left hemisphere activation for happy stimuli

| Subnuclei | Right  TE1.0 ± SD | Right  TE1.1 ± SD | Right  TE1.2 ± SD | Left  TE1.0 ± SD | Left  TE1.1 ± SD | Left  TE1.2 ± SD |
| --- | --- | --- | --- | --- | --- | --- |
| HapOrg | 6.4851±3.86 | 5.8626±3.76 | 5.8437±3.65 | 6.2266±3.19 | 6.4828±3.34 | 5.0804±2.84 |
| Hap-8nb | 7.0187±3.73 | 6.6061±4.08 | 6.2822±3.47 | 5.5773±3.84 | 6.1134±3.71 | 4.1405±3.43 |
| Hap-32nb | 7.3740±4.00 | 6.3630±4.36 | 6.5959±3.68 | 6.6248±3.49 | 6.5679±3.85 | 5.7819±2.86 |
| Hap-64nb | 4.8415±3.76 | 3.9364±3.88 | 4.5842±3.43 | 4.1322±3.58 | 4.4603±3.83 | 3.2616±2.85 |
| Average | 6.4298±3.84 | 5.6920±4.02 | 5.8265±3.56 | 5.6402±3.53 | 5.9060±3.68 | 4.5661±3.00 |
| Hemisphere | - | - | <Right | - | - | - |
| ANOVA Row | *F*=2.743, *p*=0.0462 | *F*=2.476, *p*=0.0647 | *F*=2.632 *p*=0.0531 | *F*=2.743, *p*=0.0462 | *F*=2.476, *p*=0.0647 | *F*=2.632 *p*=0.0531 |
| ANOVA Col | *F*=1.466, *p*=0.2283 | *F*=0.0983, *p*=0.7544 | *F*=4.685, *p*=0.0324 | *F*=1.466, *p*=0.2283 | *F*=0.0983, *p*=0.7544 | *F*=4.685, *p*=0.0324 |

*No follow-up performed because all subdivisions were non-significant.

#### Happy differences for left versus right hemisphere

Happy right Te1.0 was not significantly different than left Te1.0 *F*_1,3_ = 1.466, *p* = 0.2283, with stimuli significantly different by decomposition *F*_1,3_ = 2.743, *p* = 0.0462. Happy right Te1.1 was not significantly different than left Te1.1 *F*_1,3_ = 0.09831, *p* = 0.7544, with stimuli not significantly different by decomposition *F*_1,3_ = 2.476, *p* < 0.0647. Happy right Te1.2 was significantly different than left Te1.2 *F*_1,3_ = 4.685, *p* = 0.0324, with stimuli significantly different by decomposition *F*_1,3_ = 2.632, *p* = 0.0531. When accounting for comparisons by ROI (i.e. column) by decomposition (i.e. row), only Te1.2 was significant, and barely meet the statistical criterion. Nevertheless, this comparison was not accounting for the differences between hemisphere, so follow-up statistics are performed for hemispheric lateralization.

### Amygdala functional response follows identification of uncertain emotional stimuli, deactivating at chance emotional identification.

The difference between AMG ROI was analyzed to determine whether the response by decomposition to emotion identification was significant by subdivision, hemisphere, emotion, or a combination.

For the right and left hemisphere AMG response to sad emotion, no significant difference was found by subdivision LB/CM/SP, *F*_(2,6)_ = 0.2330, *p* = 0.7924 and *F*_(2,6)_ = 0.8953, *p* = 0.4103, respectively. However, the right and left hemisphere AMG response to sad emotion by decomposition was significant, *F*_(2,6)_ = 67.69, *p* < 0.0001 and *F*_(2,6)_ = 29.01, *p* < 0.0001, respectively. For the right and left hemisphere AMG response to happy emotion, no significant difference was found by subdivision LB/CM/SP, *F*_(2,6)_ = 0.1568, *p* = 0.8550 and *F*_(2,6)_ = 0.7504, *p* = 0.4737, respectively. However, the right and left hemisphere AMG response to happy emotion by decomposition was significant *F*_(2,6)_ = 79.67, *p* < 0.0001 and *F*_(2,6)_ = 140.3, *p* < 0.0001, respectively.

For AMG-ROI sad right and sad left hemisphere response, the average t-value activation difference by decomposition was 1.22 ± 0.05 and 0.30 ± 0.20 which translated into a percent change response from original excerpt of 84.45% ± 5.59% and 67.10% ± 24.48%, respectively. For AMG-ROI happy right and left hemisphere response, the average t-value activation by decomposition was 1.54 ± 0.12 and 1.14 ± 0.04 which translated into a percent change response from the original excerpt of 232.05 ± 13.06% and 262.71% ± 3.86%, respectively. The percent change response from original excerpt to the 64nb decomposition was highly significant (*p* < 0.001).

An ANOVA found the AMG modulation by stimuli significantly different *F*_(3,15)_ = 22.12, *p* < 0.001. Follow-up testing to investigate the decrease in modulation observed due to the response to uncertain emotional stimuli at 64nb revealed highly significant differences from the response to the original excerpts for right hemisphere sad ≠ 64nb *t*_(3)_ = 4.5776, *p* < 0.001, left hemisphere sad ≠ 64nb *t*_(3)_ = 10.7411, *p* < 0.001, right hemisphere happy ≠ 64nb, *t*_(3)_ = 6.6266, *p* < 0.01, left hemisphere happy ≠ 64nb *t*_(3)_ = 5.4662, *p* < 0.001.

The correlation over the decomposition was asymptotic at first and then a monotonic reversal to 64nb with low r^2^ values reflecting the change in response from the original excerpt as a linear increase, with happy-Right Hemisphere was r^2^=0.31, happy-Left Hemisphere was r^2^= 0.86, sad-Right Hemisphere was r^2^= 0.29, and sad-Left Hemisphere was r^2^=0.25. The change of response along the functional modulation was greatest for left and right hemisphere for happy or sad stimuli, from the original to 64nb stimuli.

Table S15 | Average amygdala right and left hemisphere activation for sad stimuli

| Subnuclei | Right  LB ± SD | Right  CM ± SD | Right  SP ± SD | Left  LB ± SD | Left  CM ± SD | Left  SP ± SD |
| --- | --- | --- | --- | --- | --- | --- |
| sadOrg | 1.5283±0.92 | 1.2844±0.92 | 1.5043±0.83 | 0.2736±0.94 | 0.6850±0.72 | 0.3757±0.83 |
| sad-8nb | 2.6369±0.91 | 2.5909±0.90 | 2.7083±0.85 | 0.4956±0.85 | 0.5500±0.80 | 0.5816±0.86 |
| sad-32nb | 2.0522±0.80 | 2.1197±0.81 | 2.1075±0.80 | 0.7257±1.09 | 1.2095±1.047 | 1.1776±1.12 |
| sad-64nb | 0.3447±1.02 | 0.1490±0.91 | 0.1101±0.93 | -0.4787±0.74 | -0.6919±0.69 | -0.5604±0.78 |
| Average | 1.6405±0.91 | 1.5360±0.89 | 1.6076±0.65 | 0.2541±0.91 | 0.4382±0.81 | 0.3936±0.90 |
| Hemisphere | <**Right** | <**Right** | <**Right** |  |  |  |
| ANOVA Row | *F*=20.50, *p<*0.0001 | *F*=34.42, *p<*0.0001 | *F*=37.18, *p<*0.0001 | *F*=20.50, *p<*0.0001 | *F*=34.42, *p<*0.0001 | *F*=37.18, *p<*0.0001 |
| ANOVA Col | *F*=73.49, *p<*0.0001 | *F*=52.22, *p<*0.0001 | *F*=70.45, *p<*0.0001 | *F*=73.49, *p<*0.0001 | *F*=52.22, *p<*0.0001 | *F*=70.45, *p<*0.0001 |
| ANOVA Interaction | *F*=2.892, *p=0.0382* | *F*=4.474, *p<*0.0051 | *F*=4.846, *p<*0.0032 | *F*=2.892, *p=0.0382* | *F*=4.474, *p<*0.0051 | *F*=4.846, *p<*0.0032 |

#### Sad differences for left versus right hemisphere– Right hemisphere greater activation for sad

Sad right LB was significantly different than left LB *F*_1,3_ = 73.49, *p* < 0.0001, with stimuli significantly different by decomposition *F*_1,3_ = 20.50, *p* < 0.0001. Sad right CM was significantly different than left CM *F*_1,3_ = 52.22, *p* < 0.0001, with stimuli significantly different by decomposition *F*_1,3_ = 34.42, *p* < 0.001. Sad right SP was significantly different than left SP *F*_1,3_ = 70.45, *p* < 0.0001, with stimuli significantly different by decomposition *F*_1,3_ = 37.18, *p* < 0.0001. For sad stimuli greater right hemisphere activation was consistently observed. Nevertheless, this comparison was not accounting for the differences between hemisphere, so follow-up statistics are performed for hemispheric lateralization.

Table S16 | Average amygdala right and left hemisphere activation for happy stimuli

| Subnuclei | Right  LB ± SD | Right  CM ± SD | Right  SP ± SD | Left  LB ± SD | Left  CM ± SD | Left  SP ± SD |
| --- | --- | --- | --- | --- | --- | --- |
| HapOrg | 0.5735±0.98 | 0.8740±0.91 | 0.5411±0.94 | 0.2121±1.01 | 0.6549±0.89 | 0.4376±0.95 |
| Hap-8nb | 0.1643±0.84 | 0.1605±0.78 | 0.1190±0.79 | 1.1387±0.94 | 1.5360±0.84 | 1.3450±0.80 |
| Hap-32nb | 1.5371±0.73 | 1.5594±0.73 | 1.6958±0.75 | -0.1439±0.88 | -0.1510±0.84 | -0.1662±0.87 |
| Hap-64nb | -1.0388±0.99 | -1.0456±0.88 | -1.0800±0.92 | -2.2106±0.99 | -2.2733±0.81 | -2.2385±0.82 |
| Average | 0.3090±0.89 | 0.3871±0.83 | 0.3190±0.85 | -0.2509±0.96 | -0.0584±0.85 | -0.1305±0.86 |
| Hemisphere | <**Right** | <**Right** | <**Right** |  |  |  |
| ANOVA Row | *F*=46.18, *p<*0.0001 | *F*=67.70, *p<*0.0001 | *F*=59.84, *p<*0.0001 | *F*=46.18, *p<*0.0001 | *F*=67.70, *p<*0.0001 | *F*=59.84, *p<*0.0001 |
| ANOVA Col | *F*=11.74, *p=*0.0008 | *F*=9.065, *p=*0.0032 | *F*=8.784, *p=*0.0037 | *F*=11.74, *p=*0.0008 | *F*=9.065, *p=*0.0032 | *F*=8.784, *p=*0.0037 |
| ANOVA Interaction | *F*=12.56, *p<*0.0001 | *F*=21.24, *p<0.0001* | *F*=19.94, *p<*0.0001 | *F*=12.56, *p<*0.0001 | *F*=21.24, *p<0.0001* | *F*=19.94, *p<*0.0001 |

#### Happy differences for left versus right hemisphere– Right hemisphere greater activation for Happy

Happy right LB was significantly different than left LB *F*_1,3_ = 11.74, *p* = 0.0008, with stimuli significantly different by decomposition *F*_1,3_ = 46.18, *p* < 0.0001. Happy right CM was significantly different than left CM *F*_1,3_ = 9.07, *p* < 0.0032, with stimuli significantly different by decomposition *F*_1,3_ = 67.70, *p* < 0.001. Happy right SP was significantly different than left SP *F*_1,3_ = 8.78, *p* < 0.0037, with stimuli significantly different by decomposition *F*_1,3_ = 59.84, *p* < 0.0001. For happy stimuli greater right hemisphere activation was consistently observed. Nevertheless, this comparison was not accounting for the differences between hemisphere, so follow-up statistics are performed for hemispheric lateralization.

### Deactivation of right hemispheric AMG lateralization of emotional response by emotional uncertainty.

#### Heschl’s gyrus happy and sad decompositions by hemispheric lateralized ROI activation

The activation of HG for happy decompositions were significantly different from one another *F*_(2,6)_ = 5.14, *p* < 0.01, but decompositions by ROI modulation were not significantly different by emotional uncertainty *F*_(2,6)_ = 1.68, *p* = 0.17. Meaning all HG ROI exhibited the same pattern of functional response, but the HG functional response by uncertain emotional identification was different. Since we were interested in hemispheric responses by ROI as a function of emotional identification, were performed follow-up tests despite the non-significant findings in the ANOVA. A follow-up t-test for happy found HG-Te1.0 significantly different from HG-Te1.1 *t*_(3)_ = 8.16, *p* < 0.01 (HG-Te1.0 was right leaning and HG-Te1.1 was left leaning) and HG-Te1.1 significantly different from HG-Te1.2 *t*_(3)_ = 4.87, *p* < 0.05 (HG-Te1.1 was left leaning and HG-Te1.2 was right leaning), with HG-Te1.0 not significantly different from HG-Te1.2 *t*_(3)_ = 0.54, *p* = 0.63 (both right leaning). Differences for activation HG-TE1.0-Hap (*t*_3_ = 1.39, *p* = 0.26), HG-TE1.1-Hap (*t*_3_ = 1.14, *p* = 0.34), HG-TE1.2-Hap (*t*_3_ = 1.21, *p* = 0.31). Average change by decomposition in modulation of t-value in activation HG-TE1.0-Hap 268.82% ± 17.53%, HG-TE1.1-Hap 55.90% ± 13.21%, HG-TE1.2-Hap 431.94% ± 90.64%.

The activation of HG-ROI for sad decompositions were significantly different from one another *F*_(2,6)_ = 5.45, *p* < 0.005, but decompositions by ROI modulation were not significantly different by emotional uncertainty *F*_(2,6)_ = 2.21, *p* = 0.09. Since we were interested in hemispheric responses by ROI as a function of emotional identification, were performed follow-up tests despite the non-significant findings in the ANOVA. A follow-up t-test for sad found HG-Te1.0 significantly different from HG-Te1.1 *t*_(3)_ = 7.87, *p* < 0.01 (HG-Te1.0 was right leaning and HG-Te1.1 was left leaning), and HG-Te1.1 significantly different from HG-Te1.2 *t*_(3)_ = 3.98, *p* < 0.05 (HG-Te1.1 was left leaning and HG-Te1.2 was right leaning), with HG-Te1.0 not significantly different from HG-Te1.2 *t*_(3)_ = 0.89, *p* = 0.44 (both right leaning). Differences for activation HG-TE1.0-sad (*t*_3_ = 0.27, *p* = 0.80), HG-TE1.1-sad (*t*_3_ = 1.97, *p* = 0.14), HG-TE1.2-sad (*t*_3_ = 0.79, *p* = 0.49). Average change by decomposition in modulation of t-value in activation HG-TE1.0-sad 87.78% ±2.78%, HG-TE1.1-sad 55.27% ± 5.49%, HG-TE1.2-sad 101.22% ± 17.42%.

In summary for activation, happy and sad emotional functioning in HG was right hemispheric lateralized for Te1.1 and Te1.2 and left hemispheric lateralized for Te1.0 subdivisions.

Since we were interested in hemispheric responses by ROI as a function of emotional identification, were performed follow-up tests despite the non-significant findings in the ANOVA.

#### Heschl’s gyrus happy and sad decompositions by hemispheric lateralized ROI deactivation

The deactivation of HG-ROI for happy decomposition were not significantly different from one another by ROI *F*_(2,6)_ = 0.36, *p* = 0.69, but the decompositions by emotional uncertainty were significantly different other bands *F*_(2,6)_ = 6.69, *p* < 0.0001. Average change by decomposition in modulation of t-value in deactivation HG-TE1.0-Hap 274.29% ± 171.61%, HG-TE1.1-Hap 248.72% ± 28.40%, HG-TE1.2-Hap 780.56% ± 156.41%. The deactivation of HG-ROI for sad decompositions were significantly different from one another *F*_(2,6)_ = 4.80, *p* < 0.01, but the decompositions by emotional uncertainty were not significantly different *F*_(2,6)_ = 1.42, *p* = 0.24. Nevertheless, a follow-up t-test for sad failed to find significant differences between ROI; HG-Te10/HG-Te1.1 *t*_(3)_ = 0.74, *p* = 0.51, HG-Te1.1/HG-Te1.2 *t*_(3)_ = 0.13, *p* = 0.91, and HG-Te1.0/HG-Te1.2 *t*_(3)_ = 0.93, *p* = 0.44. The difference in deactivation was near significant for sad emotion HG-TE1.1 (being left leaning; *t*_3_ = 2.95, *p* = 0.06), but not for sad HG-TE1.0 (mixed left and right by decomposition; *t*_3_ = 1.96, *p* = 0.14), and sad HG-TE1.2-sad (mixed by decomposition; *t*_3_ = 1.90, *p* = 0.15). Average change by decomposition in modulation of t-value in deactivation HG-TE1.0-sad 171.17% ±28.96, HG-TE1.1-sad 92.67 ± 62.02%, HG-TE1.2-sad 104.255% ± 462.93%.

#### Amygdala happy and sad decompositions by hemispheric lateralized ROI in activation

The activation of AMG-ROI for happy was not significantly different from one another *F*_(2,6)_ = 0.01, *p* = 0.91, but decompositions by ROI modulation were significantly different from other decompositions *F*_(2,6)_ = 27.18, *p* < 0.0001. Amygdala subdivisions were not significantly different from one another for happy response: AMG-CM/AMG-LB *t*_(3)_ = 0.15, *p* = 0.89, AMG-LB/AMG-SP *t*_(3)_ = 0.07, *p* = 0.95, and AMG-CM/AMG-SP *t*_(3)_ = 0.14, *p* = 0.90 (prominent right leaning). Differences in activation for happy; AMG-CM-Hap (*t*_3_ = 0.07, *p* = 0.95), AMG-LB-Hap (*t*_3_ = 0.15, *p* = 0.89), AMG-SP-Hap (*t*_3_ = 0.53, *p* = 0.63) were not significantly different (right leaning). Average change by decomposition in modulation of t-value in activation for happy were AMG-CM 1230.00% ± 21.25%, AMG-LB 4633.30% ± 26.67, AMG-SP 631.25% ± 34.20%. The activation for AMG-ROI for happy responses were right leaning, but average was left leaning for original and 8nb, and right leaning for decompositions 32nb and 64nb.

The activation of AMG-ROI for sad was not significantly different from one another *F*_(2,6)_ = 0.97, *p* = 0.38, but decompositions by ROI modulation were significantly different from other decompositions *F*_(2,6)_ = 11.58, *p* < 0.0001. A follow-up t-test for sad found AMG-CM significantly different from AMG-SP, *t*_(3)_ = 3.82, *p* < 0.05 (but here both were right leaning) with AMG-CM/AMG-LB *t*_(3)_ = 1.634, *p* = 0.20, and AMG-LB/AMG-SP *t*_(3)_ = 2.30, *p* = 0.11 non-significant (all right leaning). Differences in activation for sad; AMG-CM (*t*_3_ = 0.99, *p* = 0.40), AMG-LB (*t*_3_ = 0.90, *p* = 0.44), AMG-SP (*t*_3_ = 0.94, *p* = 0.42), were not significantly different (right leaning). Average change by decomposition in modulation of t-value in activation for sad were AMG-CM 158.33 ± 34%.08, AMG-LB 117.42% ± 31.27, AMG-SP 211.83% ± 33.63%. The activation for AMG-ROI for sad responses were right leaning, with no change in activation by decomposition.

#### Amygdala happy and sad decompositions by hemispheric lateralized ROI in deactivation

The deactivation of AMG-ROI for happy was significantly different from one another *F*_(2,6)_ = 3.78, *p* < 0.01, with decompositions by ROI modulation significantly different from other decompositions *F*_(2,6)_ = 37.25, *p* < 0.0001. A follow-up post-hoc t-test for the amygdala subdivisions was not significantly different: AMG-CM/AMG-LB *t*_(3)_ = 1.148, *p* = 0.33, AMG-LB/AMG-SP *t*_(3)_ = 1.142, *p* = 0.34, and AMG-CM/AMG-SP *t*_(3)_ = 0.91, *p* = 0.43 (mixed response). Differences in deactivation for happy; AMG-CM (*t*_3_ = 0.81, *p* = 0.48), AMG-LB (*t*_3_ = 1.32, *p* = 0.28), AMG-SP (*t*_3_ = 1.12, *p* = 0.34). Average change by decomposition in modulation of t-value in deactivation for happy was AMG-CM 1566.67% ± 27.16, AMG-LB 94.16% ± 33.75%, AMG-SP 675.00% ± 37.36%. The deactivation followed a mixed response with left for original followed by right for 8nb, succeeded by left responses for 32nb and 64nb.

The deactivation of AMG-ROI for sad was not significantly different from one another *F*_(2,6)_ = 0.37, *p* = 0.69, but decompositions by ROI modulation were significantly different from other decompositions *F*_(2,6)_ = 3.51, *p* < 0.01. A follow-up post-hoc t-test for the amygdala subdivisions was not significantly different: AMG-CM/AMG-LB *t*_(3)_ = 0.86, *p* = 0.45, AMG-LB/AMG-SP *t*_(3)_ = 0.43, *p* = 0.70, and AMG-CM/AMG-SP *t*_(3)_ = 1.15, *p* = 0.33 (left leaning). Differences in deactivation for sad; AMG-CM (*t*_3_ = 2.86, *p* = 0.06) – near significant, AMG-LB (*t*_3_ = 2.10, *p* = 0.13), AMG-SP (*t*_3_ = 2.87, *p* = 0.06) – near significant (left leaning). Average change by decomposition in modulation of t-value in deactivation sad; AMG-CM 816.67% ± 62.5%, AMG-LB 13.13% ± 15.48%, AMG-SP 80% ± 944.44%.

Table S17 | Hemispheric lateralization of functional emotional modulation

|  |  | Hap-org | Hap-8nb | Hap-32nb | Hap-64nb | Sad-org | Sad-8nb | Sad-32nb | Sad-64nb |
| --- | --- | --- | --- | --- | --- | --- | --- | --- | --- |
| Activation |  |  |  |  |  |  |  |  |  |
|  | AMG-CM | 0.01±0.91 | -1.01±0.81 | 0.94±0.82 | 0.22±0.35 | 0.48±0.89 | 1.61±1.20 | 0.49±0.94 | 0.46±0.60 |
|  | AMG-LB | -0.02±0.90 | -0.76±0.83 | 0.72±0.91 | 0.16±0.34 | 0.44±0.97 | 1.20±1.27 | 0.41±0.95 | 0.41±0.66 |
|  | AMG-SP | -0.16±0.77 | -0.83±0.80 | 0.88±0.95 | 0.23±0.34 | 0.31±0.98 | 1.21±1.28 | 0.24±0.96 | 0.34±0.59 |
| Avg-AMG |  | -0.06±0.86 | -0.86±0.81 | 0.85±0.90 | 0.20±0.34 | 0.41±0.95 | 1.34±1.25 | 0.38±0.95 | 0.40±0.62 |
|  | HG-TE1.0 | 0.31±1.94 | 1.42±2.0 | 0.45±2.50 | 0.48±2.04 | 0.60±2.28 | 0.47±2.19 | 0.21±2.24 | 1.40±2.29 |
|  | HG-TE1.1 | -0.65±2.22 | -0.14±2.78 | -0.50±2.61 | -0.72±2.76 | -1.17±3.22 | -0.56±2.75 | -0.90±2.73 | 0.09±2.77 |
|  | HG-TE1.2 | 0.24±1.07 | 1.4±2.94 | 0.07±2.38 | 0.69±1.90 | 0.82±1.99 | 0.16±2.53 | -0.15±2.14 | 1.37±2.25 |
| Avg-HG |  | -0.03±1.74 | 0.90±2.58 | 0.01±2.50 | 0.15±2.23 | 0.08±2.50 | 0.02±2.50 | -0.28±2.37 | 0.95±2.44 |
| Deactivation |  |  |  |  |  |  |  |  |  |
|  | AMG-CM | 0.04±0.54 | -0.43±0.47 | 0.56±0.47 | 0.98±0.10 | 0.02±0.32 | 0.37±0.48 | 0.38±0.34 | 0.51±0.64 |
|  | AMG-LB | 0.97±0.80 | -0.38±0.53 | 0.58±0.55 | 1.01±1.07 | 0.33±0.56 | 0.36±0.53 | 0.41±0.43 | 0.46±0.56 |
|  | AMG-SP | -0.08±0.58 | -0.39±0.50 | 0.57±0.54 | 0.92±1.07 | 0.15±0.03 | 0.39±0.52 | 0.36±0.39 | 0.45±0.62 |
| Avg-AMG |  | 0.31±0.64 | -0.40±0.50 | 0.57±0.52 | 0.97±0.75 | 0.16±0.30 | 0.37±0.51 | 0.38±0.39 | 0.47±0.61 |
|  | HG-TE1.0 | -0.35±0.27 | 0.82±0.66 | -0.47±0.27 | -0.05±0.88 | 0.37±0.61 | -0.02±0.77 | -0.75±0.52 | 0.03±0.64 |
|  | HG-TE1.1 | 0.26±0.54 | -0.66±0.58 | 0.36±0.71 | 0.36±1.00 | -0.50±0.43 | 0.55±0.72 | 0.34±0.54 | 0.47±0.87 |
|  | HG-TE1.2 | -0.12±0.26 | 0.76±0.67 | -0.55±0.27 | 0.07±0.68 | 0.47±0.09 | -0.27±0.79 | 0.31±0.36 | 0.16±0.48 |
| Avg-Hg |  | -0.07±0.36 | 0.31±0.64 | -0.22±0.42 | 0.13±0.85 | 0.11±0.38 | 0.09±0.76 | -0.03±0.47 | 0.22±0.67 |

Table. Hemispheric lateralization of functional emotional modulation. The table demonstrates the laterality of emotion for the ROI delineations under investigation. The following abbreviations; AMG- amygdala, CM- centromedial, LB- laterobasal, SP- superficial, HG –Heschl’s gyrus, TE1.0 Middle HG, TE1.1 Posteromedial HG, and TE1.2 Anterolateral HG. The metrics for activation and deactivation hemisphere laterality calculations are discussed in the methods section. Activation hemispheric laterality calculations reflect [R]-[L], were R and L are right and left hemispheres, respectively. The resultant difference for activation calculations was a positive t-value reflecting a right hemisphere sided laterality and a negative t-value deactivation reflecting left hemisphere laterality. Deactivation hemispheric laterality calculations reflect [-R]-[-L], with numbers in the negative range reflecting the opposite observation; the resultant difference for deactivation calculations was a negative number reflecting a right hemisphere sided laterality and a positive number a left hemisphere laterality.

Table S18 | Approximate hemispheric lateralization of functional emotional modulation

|  |  | Hap-org | Hap-8nb | Hap-32nb | Hap-64nb | Sad-org | Sad-8nb | Sad-32nb | Sad-64nb |
| --- | --- | --- | --- | --- | --- | --- | --- | --- | --- |
| Activation |  |  |  |  |  |  |  |  |  |
|  | AMG-CM | Right | Left | Right | Right | Right | Right | Right | Right |
|  | AMG-LB | Left | Left | Right | Right | Right | Right | Right | Right |
|  | AMG-SP | Left | Left | Right | Right | Right | Right | Right | Right |
| Avg-AMG |  | **Left** | Left | Right | Right | Right | Right | **Right** | **Right** |
|  | HG-TE1.0 | Right | Right | Right | Right | Right | Right | Right | Right |
|  | HG-TE1.1 | Left | Left | Left | Left | Left | Left | Left | Right |
|  | HG-TE1.2 | Right | Right | Right | Right | Right | Right | Left | Right |
| Avg-HG |  | Left | Right | Right | Right | Right | Right | Left | Right |
| Deactivation |  |  |  |  |  |  |  |  |  |
|  | AMG-CM | Left | Right | Left | Left | Left | Left | Left | Left |
|  | AMG-LB | Left | Right | Left | Left | Left | Left | Left | Left |
|  | AMG-SP | Right | Right | Left | Left | Left | Left | Left | Left |
| Avg-AMG |  | **Left** | Right | Left | Left | Left | Left | **Left** | **Left** |
|  | HG-TE1.0 | Right | Left | Right | Right | Left | Right | Right | Left |
|  | HG-TE1.1 | Left | Right | Left | Left | Right | Left | Left | Left |
|  | HG-TE1.2 | Right | Left | Right | Left | Left | Right | Left | Left |
| Avg-Hg |  | Right | Left | Right | Left | Left | Left | Right | Left |

**Table.** Activation hemispheric laterality calculations reflect [R]-[L], were R and L are right and left hemispheres, respectively. Positive and negative t-values are right and left hemispheric leaning, respectively. Deactivation hemispheric laterality calculations reflect [-R]-[-L]. Negative and positive t values are right and left hemispheric leaning, respectively.

Fig. S8 | Zoom of ROI subdivisions


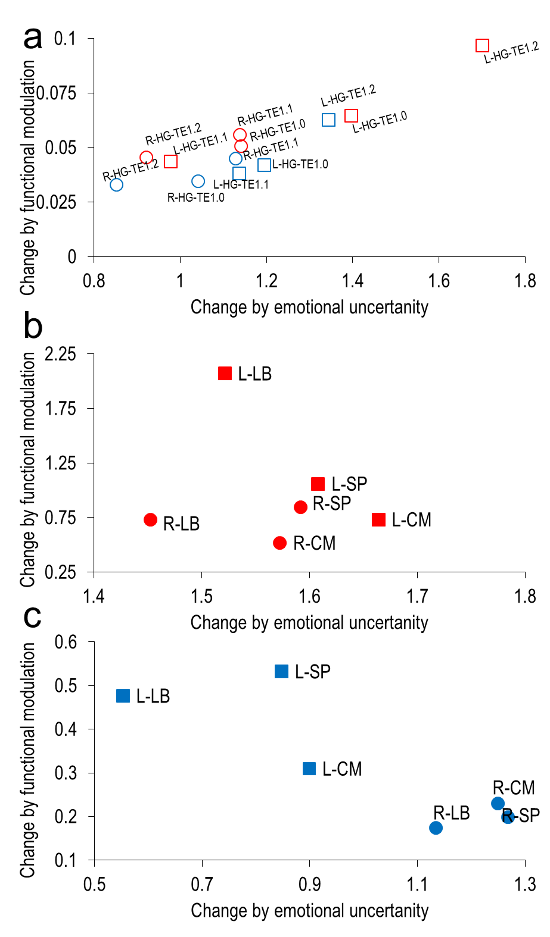


**Figure.** Zoom of HG and AMG from figure in manuscript. Figures are weighted change: absolute functional modulation of ROI as a factor of absolute percent change by emotional uncertainty. The average activation map t value derived from the ROI volume (y-axis) was correlated with percent identification by decomposition (y-axis) with representation for each ROI grouped (left hemisphere-square; right hemisphere-circle; blue-sad stimuli red-happy; AMG-filled; HG-unfilled). (A) The HG-ROI were unnamed in Figure 5 of the manuscript due to clustering and considerable overlap. (B) A zoom of AMG-ROI for happy from Figure 5. (C) A zoom of AMG-ROI for sad from Figure 5.

### Contralateral hemispheric HG activation balances lateralization to uncertain emotion; deactivation of left AMG hemispheric functioning follows uncertain stimuli

Hemispheric lateralization of HG and AMG was investigated due to the observed differences in modulation by hemisphere as a response to certain and uncertain emotion. The left hemisphere HG activation to sad emotion by decomposition was significant *F*_(2,6)_ = 6.439, *p* = 0.0004, while the right hemisphere HG was not significant *F*_(2,6)_ = 2.014, *p* = 0.1137, whereas the right and left hemisphere HG activation to happy emotion by decomposition was significant *F*_(2,6)_ = 3.743, *p* = 0.0121 and *F*_(2,6)_ = 4.438, *p* = 0.0049, respectively. Follow-up testing for sad and happy found HG-Te1.0 and HG-Te1.2 were right leaning while HG-Te1.1 was left leaning for both emotions (*p* < 0.01; Fig. S7 | Difference mapping of hemispheric lateralization resolvability of uncertain emotion; Table S17 and S18). Activation patterns by hemispheric lateralization of HG for happy (*F*_2,6_ = 5.14, *p* < 0.01) and sad (*F*_2,6_ = 5.45, *p* < 0.005) emotion were significantly different with increasing uncertainty (Fig. S7 | Difference mapping of hemispheric lateralization resolvability of uncertain emotion A, left hand column), whereas deactivation patterns were significant for sad only *F*_2,6_ = 4.80, *p* < 0.01 (Fig. S7 | Difference mapping of hemispheric lateralization resolvability of uncertain emotion A, right hand column). Considerable change in t-value modulation by decomposition for HG subregions was found for both activation and deactivation patterns. Although little variation in t-value modulation by emotion certainty occurs for HG (i.e. difference in activation along the row in Fig. S7 | Difference mapping of hemispheric lateralization resolvability of uncertain emotion; the magnitude of AMG was greater), the change in emotional identification appeared to be left hemisphere leaning (Table S18 | Approximate hemispheric lateralization of functional emotional modulation).

The right and left hemisphere AMG activation for sad, *F*_(2,6)_ = 67.69, *p* < 0.0001 and *F*_(2,6)_ = 29.01, *p* < 0.0001, respectively, and happy *F*_(2,6)_ = 79.67, *p* < 0.0001 and *F*_(2,6)_ = 140.3, *p* < 0.0001, respectively, emotion by decomposition were considerably significant. Follow-up testing for AMG subdivisions for activation in response to sad were entirely right learning with happy eliciting CM right leaning, LB and SP left leaning for certain emotion, with all responses right leaning for uncertain emotion and emotion identified at chance (*p* < 0.05; D; Table S15 | Average amygdala right and left hemisphere activation for sad stimuli, Table S16 | Average amygdala right and left hemisphere activation for happy stimuli, and Table S18 | Approximate hemispheric lateralization of functional emotional modulation). Activation pattern was significant for AMG subdivisions by magnitude, but not by hemispheric lateralization, with a right hemispheric activation for certain emotion, but little activation for emotion identified as chance. Deactivation patterns for sad and happy emotion by decomposition were significantly different *F*_(2,6)_ = 3.51, *p* < 0.01 and *F*_(2,6)_ = 37.25, *p* < 0.0001, respectively (Fig. S7 | Difference mapping of hemispheric lateralization resolvability of uncertain emotion B; here note the difference between the last row for happy and sad, comparing deactivation with activation; in particular the pattern for sad was matched in magnitude, but appears changed in subdivision). The deactivation pattern for AMG subdivisions for happy were significantly different *F*_(2,6)_ = 3.78, *p* < 0.01, but not for sad *F*_(2,6)_ = 0.37, *p* = 0.69, with the magnitude of change being the contributor to the predominate left hemispheric lateralization for certain and uncertain emotion (Fig. S6 | Functional resolvability of uncertain emotional sound). Left hemispheric deactivations were noted for happy and sad uncertain emotion, but compared with certain emotion did not significantly change sidedness by hemisphere (Fig. S7 | Difference mapping of hemispheric lateralization resolvability of uncertain emotion). Follow-up testing for sad and happy found no differences in ROI by subdivision with a prominent left leaning hemispheric lateralization for CM and LB for certain emotion with SP right leaning, with all subdivisions left leaning deactivated during uncertain emotion. In Fig. S7 | Difference mapping of hemispheric lateralization resolvability of uncertain emotion, the change in lateralization as a factor of uncertain emotion was noted with differences by ROI, emotion (happy and Sad) and hemisphere. A marked pattern of left hemisphere AMG deactivation for uncertain emotional stimuli was mirrored to activation of right hemisphere AMG emotional resolvability where happy responses elicited the greatest AMG deactivation pattern coupled to emotional certainty. Sad emotion, although displaying significant deactivation was matched by activation more consistently; therefore lacked definitive sidedness. When plotted as a function of absolute weighted percent change, the above differences between ROI subdivisions become marked (Fig. S8 | Zoom of ROI subdivisions).

## Amygdala sexual dimorphism in emotional resolvability by hemispheric lateralization

The present experiment found significant modulation in ROI as a function of emotional certainty by hemispheric lateralization. In this aspect of the experiment, we asked if sexual dimorphism exists in response to resolving emotion could contribute to part of the hemispheric lateralization. Hemispheric lateralization differences between the sexes could account for the differing propensity to emotional content as postulated by Geschwind and Galabruda (Geschwind, Galaburda, 1985). We know very little about why the sexes differ in their perception of emotion (Witteman et al., 2012; Gosselin et al., 2007; Gosselin et al., 2005; Frühholz S, et al. 2005; Kumar et al., 2012; Wager et al., 2003); therefore, assessing uncertain emotion on a range of levels will assist in determining if female and males interpret emotions differently. We hypothesized that if sexual differences in perception of emotion result functionally (Witteman et al., 2012; Gosselin et al., 2007; Gosselin et al., 2005; Frühholz S, et al. 2005; Kumar et al., 2012; Wager et al., 2003; Geschwind, Galaburda, 1985) they will show unique patterns based on their psychophysical profiles of emotion resolvability. For example, if a difference in response to emotion exists between females and males they should respond differently to varying emotion: certain, uncertain and chance identifiable emotion.

Average sexual dimorphism, female different than male amygdala response to sad and happy were significantly different as a function of emotional resolvability, df=3,15, F=196.6, p<0.0001 and df=3,15, F=210.7, p<0.0001, respectively. Meaning females and males differed in their entire amygdala responses to resolving uncertain emotion. Average sexual dimorphism, female different than male amygdala by subdivision response to happy and sad emotion was significantly different as a function of emotional resolvability, df=5,15, F=5.217, p<0.0001 and df=5,15, F=14.69, p<0.0001, respectively. Meaning amygdala subdivisions responded significantly different to resolving emotion between females and males. The interaction of emotional resolvability as a function of subdivision response to sad and happy was significantly different, interaction F=43.13, p<0.0001 and interaction F=74.48, p<0.0001, respectively. Meaning a significant difference exists between the sexes in how each amygdala subdivision responds to uncertain emotion.

### Happy absolute [Female] – [Male] sexual dimorphism

The absolute difference between female and males for happy resulted in several significant findings (Table S19 | [Female] – [Male] Averages Happy and Table S20 | [Female] – [Male] predominance sided average for Happy). An ANOVA of row (i.e. subdivision) by column (i.e. decomposition) for female different than male for **left** hemisphere response to happy found row df=2,6, F=0.033, p=0.9672, column df=3,6, F=14.73, p<0.0001, and interaction of row by column F=0.7971, p=0.5738. An ANOVA of row (i.e. subdivision) by column (i.e. decomposition) for female different than male for **right** hemisphere response to happy found row df=2,6, F=0.3746, p=0.6883, column df=3,6, F=10.80, p<0.0001, interaction of row by column F=0.1081, p=0.9954. The absolute [Female] – [Male] average for responses to happy found a significant difference for decomposition for both the left and right hemisphere. No difference was found for the response by subdivision. The absolute difference between female and male for hemispheric lateralization (left different than right) revealed a significant difference for Original (t=5.159,p=0.0356), and 32nb (t=12.51,p=0.0063), but not for 8nb nor chance 64nb. The absolute difference [Female] – [Male] average response to happy for emotional resolvability over the entire series of decompositions for left and right amygdala subdivisions was significant for all ROI, except right hemispheric SP response.

### Sad absolute [Female] – [Male] sexual dimorphism

The absolute difference between female and males for sad resulted in several significant findings (Table S22 | [Female] – [Male] Averages Sad and Table S23 | [Female] – [Male] predominance sided average for Sad). An ANOVA of row (i.e. subdivision) by column (i.e. decomposition) for female different than male for **left** hemisphere response to sad found row df=2,6, F=0.3400, p=0.7124, column df=3,6, F=3.772, p=0.0122, and interaction of row by column F=0.2372, p=0.9636. An ANOVA of row (i.e. subdivision) by column (i.e. decomposition) for female different than male for **right** hemisphere response to sad found row df=2,6, F=0.3856, p=0.6808, column df=3,6, F=22.33, p<0.0001, interaction of row by column F=0.1101, p=0.9951. The absolute [Female] – [Male] average for responses to sad found a significant difference for decomposition for both the left and right hemisphere. No difference was found for the response by subdivision. The absolute difference between female and male for hemispheric lateralization (left different than right) revealed a significant difference for Original (t=17.21, p=0.0034), and 8nb (t=11.69, p=0.0072), but not for 32nb nor chance 64nb. The absolute difference [Female] – [Male] average response to sad for emotional resolvability over the entire series of decompositions for right amygdala subdivisions was significant for all ROI, while left amygdala subdivisions where non-significant upon follow-up statistical testing. No significant differences for left side amygdala subdivisions was found for greater female over male responses.

Table S19 | [Female] – [Male] Averages Happy

| Side | Area | Hap-org | Hap-8nb | Hap-32nb | Hap-64nb | Decomposition | Sig |
| --- | --- | --- | --- | --- | --- | --- | --- |
| Left-Hap |  |  |  |  |  |  |  |
|  | AMG-LB | 0.2480±0.9483 (0.0338) | -0.8096±1.0970 (0.0392) | 0.2316±0.7669 (0.0274) | -0.2686±1.1662 (0.0416) | df=3,44, F=2.975, p=0.0418 | Yes |
|  | AMG-CM | 0.1449±0.9389 (0.0408) | -1.3814±1.1738 (0.0510) | 0.4468±0.7348 (0.0319) | 0.3630±1.0734 (0.0466) | df=3,44, F=8.969, p<0.0001 | Yes |
|  | AMG-SP | 0.2596±0.8954 (0.0343) | -0.9880±1.1366 (0.0436) | 0.3359±0.7466 (0.0286) | -0.0145±1.1629 (0.0446) | df=3,44, F=4.457, p<0.0081 | Yes |
| Stat L-H | Avg | 0.2175±0.9275 | -1.0600±1.1360 | 0.3381±0.7490 | 0.0266±1.1342 |  | NA |
| Stat L-H | Avg | t=0.8123, p=0.4338 | t=3.2323, p=0.0080* | t=1.5637, p=0.1462 | t=0.0812, p=0.9367 |  |  |
| Stat L-H | Diff | No | Yes | No | No |  |  |
| Right-Hap |  |  |  |  |  |  |  |
|  | AMG-LB | -0.4609±0.9473 (0.0397) | -0.7208±1.0306 (0.0432) | 0.5288±0.9947 (0.0417) | -0.0172±1.0367 (0.0435) | df=3,44, F=3.577, P=0.0212 | Yes |
|  | AMG-CM | -0.3087±0.9366 (0.0398) | -0.7036±1.0415 (0.0442) | 0.7128±0.9533 (0.0405) | 0.0131±0.9442 (0.0401) | df=3, 44, F=4.585, P=0.0070 | Yes |
|  | AMG-SP | -0.1204±0.8645 (0.0337) | -0.5163±0.9683 (0.0377) | 0.5606±0.9881 (0.0385) | 0.0884±0.8936 (0.0348) | df=3,44, F=2.789, p=0.0516 | No |
| Stat R-H | Avg | -0.3848±0.9419 | -0.7122±1.0361 | 0.6208±0.974 | -0.0021±0.9905 |  |  |
| Stat R-H | Avg | t=1.4152, p=0.1847 | t=2.3812, p=0.0364* | t=2.2079, p=0.0494 | t=0.0073, p=0.9943 |  |  |
| Stat R-H | Diff | No | Yes | No | No |  |  |
| Left≠Right | AMG | t=5.159,p=0.0356 | t=2.392,p=0.1392 | t=12.51,p=0.0063 | t=0.0081,p=0.9943 |  |  |
| Left≠Right | AMG | Yes | No | Yes | No |  |  |

**Table.** Difference table for [Female] – [Male], where positive numbers indicate female greater and negative numbers indicate male greater. * denotes statistical significance p<0.05.

Table S20 | [Female] – [Male] predominance sided average for Happy

|  |  | Hap-org | Hap-8nb | Hap-32nb | Hap-64nb |  |
| --- | --- | --- | --- | --- | --- | --- |
| Left-Hap |  |  |  |  |  |  |
|  | AMG-LB | Female | Male | Female | Male |  |
|  | AMG-CM | Female | Male | Female | Female |  |
|  | AMG-SP | Female | Male | Female | Male |  |
| Right-Hap |  |  |  |  |  |  |
|  | AMG-LB | Male | Male | Female | Male |  |
|  | AMG-CM | Male | Male | Female | Female |  |
|  | AMG-SP | Male | Male | Female | Female |  |

Table S21 | Average Female and Male amygdala happy responses

| Area |  | HappyOrg | Happy-8nb | Happy-32nb | Happy-64nb | Decomposition | Sig |
| --- | --- | --- | --- | --- | --- | --- | --- |
| Male |  |  |  |  |  |  |  |
|  | Left |  |  |  |  |  |  |
|  | LB | -0.0655±0.8709 | 1.4295±0.7806 | -0.0340±0.5384 | -1.0414±0.6224 | df=3,44, F=24.35, p<0.0001 | Yes |
|  | CM | 0.2028±0.7885 | 1.6033±0.8405 | -0.1287±0.4812 | -1.1560±0.5483 | df=3,44, F=33.51, p<0.0001 | Yes |
|  | SP | 0.1023±0.8615 | 1.4828±0.7354 | -0.0975±0.4930 | -1.0982±0.5572 | df=3,44, F=29.51, p<0.0001 | Yes |
|  | Avg | 0.0799±0.8403 | 1.5052±0.7855 | -0.0867±0.5042 | -1.0985±0.5760 | t=0.1866, p=0.8639 | No |
|  | Stat | t=0.3294 , p=0.7481 | t=6.6380, p<0.0001* | t=0.5957, p=0.5634 | t=6.6065, p<0.0001* |  |  |
|  |  | No | Yes | No | Yes |  |  |
|  | Right |  |  |  |  |  |  |
|  | LB | 0.8603±0.8213 | 0.7627±0.6904 | 0.5152±0.7453 | -0.4340±0.7686 | df=3,44, F=7.309, p<0.0004 | Yes |
|  | CM | 0.9175±0.7681 | 0.7296±0.6467 | 0.4569±0.6751 | -0.4633±0.7777 | df=3,44, F=8.697, p=0.0001 | Yes |
|  | SP | 0.6266±0.6873 | 0.6796±0.6462 | 0.5634±0.7020 | -0.5521±0.7083 | df=3,44, F=8.854, p=0.0001 | Yes |
|  | Avg | 0.8015±0.7589 | 0.7240±0.6611 | 0.5118±0.7075 | -0.4831±0.7515 | t=1.3086, p=0.2819 | No |
|  | Stat | t=4.6344 , p=0.0007* | t=3.7937, p=0.0030* | t=2.5059, p=0.0292* | t=2.2269, p=0.0478* |  |  |
|  |  | Yes | Yes | Yes | Yes |  |  |
| Female |  |  |  |  |  |  |  |
|  | Left |  |  |  |  |  |  |
|  | LB | 0.2331±0.6620 | 0.0744±0.6690 | 0.2363±0.5777 | -1.0168±0.7718 | df=3,44, F=9.640, p<0.0001 | Yes |
|  | CM | 0.4087±0.5681 | 0.1454±0.6773 | 0.3986±0.4915 | -0.8778±0.7280 | df=3,44, F=11.50, p<0.0001 | Yes |
|  | SP | 0.3903±0.5673 | 0.1074±0.6677 | 0.3094±0.5645 | -0.9512±0.7828 | df=3,44, F=10.92, p<0.0001 | Yes |
|  | Avg | 0.3440±0.5991 | 0.1091±0.6713 | 0.3148±0.5446 | -0.9486±0.7609 | t=0.1478, p=0.8919 | No |
|  | Stat | t=1.9891 , p=0.0721 | t=0.5630, p=0.5847 | t=2.0024, p=0.0705 | t=4.3186, p=0.0012* |  |  |
|  |  | No | No | No | Yes |  |  |
|  | Right |  |  |  |  |  |  |
|  | LB | 0.1484±0.7237 | -0.1937±0.6452 | 0.9275±0.7676 | -0.3351±0.8625 | df=3,44, F=6.738, p=0.0008 | Yes |
|  | CM | 0.4304±0.7299 | -0.1286±0.7199 | 1.1049±0.7184 | -0.3640±0.8166 | df=3,44, F=9.191, p<0.0001 | Yes |
|  | SP | 0.3054±0.7568 | -0.0666±0.6546 | 0.9643±0.7697 | -0.2873±0.7862 | df=3,44, F=6.514, p=0.0010 | Yes |
|  | Avg | 0.2947±0.7368 | -0.1296±0.6732 | 0.9989±0.7519 | -0.3288±0.7609 | t=0.7109, p=0.5284 | No |
|  | Stat | t=1.3855 , p=0.1933 | t=0.6669, p=0.5186 | t=4.6021, p=0.0008* | t=1.4969, p=0.1625 |  |  |
|  |  | No | No | Yes | No |  |  |

Table S22 | [Female] – [Male] Averages Sad

| Side | Area | Sad-org | Sad-8nb | Sad-32nb | Sad-64nb | Decomposition | Sig |
| --- | --- | --- | --- | --- | --- | --- | --- |
| Left-Sad |  |  |  |  |  |  |  |
|  | AMG-LB | 0.5244±0.7969 (0.0284) | 0.4323±0.8787 (0.0314) | 0.9621±0.9014 (0.0322) | -0.0471±0.9637 (0.0344) | df=3,44, F=2.612, P=0.0632 | No |
|  | AMG-CM | 0.5397±0.9380 (0.0407) | 0.4530±0.9515 (0.0413) | 1.004±1.0609 (0.0461) | 0.4722±0.9342 (0.0406) | df=3,44, F=0.8610, p=0.4684 | No |
|  | AMG-SP | 0.6766±0.9916 (0.0380) | 0.5443±0.9428 (0.0361) | 0.9232±0.9273 (0.0355) | 0.2310±1.1207 (0.0429) | df=3,44, F=0.1.001, p=0.4015 | No |
| Stat L-S | Avg | 0.5802±0.9088 | 0.4765±0.9243 | 0.9631±0.9632 | 0.2187±1.0062 |  | NA |
| Stat L-S | Avg | t=2.2116, p=0.0491 | t=1.7858, p=0.1017 | t=3.4637, p=0.0053* | t=0.7529, p=0.4673 |  |  |
| Stat L-S | Diff | No | No | Yes | No |  |  |
| Right-Sad |  |  |  |  |  |  |  |
|  | AMG-LB | -0.5296±1.3730 (0.0576) | 1.8944±1.7292 (0.0725) | 1.0127±1.0682 (0.0448) | 0.4757±1.0453 (0.0438) | df=3,44, F=6.945, p=0.0006 | Yes |
|  | AMG-CM | -0.4001±1.3538 (0.0575) | 2.3916±1.8026 (0.0765) | 1.1219±0.9508 (0.0404) | 0.6279±1.0568 (0.0449) | df=, F=9.086, p<0.0001 | Yes |
|  | AMG-SP | -0.1869±1.2828 (0.0500) | 2.1351±1.7333 (0.0675) | 0.9996±0.9960 (0.0388) | 0.6425±1.1082 (0.0432) | df=3,44, F=6.483, p=0.0010 | Yes |
| Stat R-S | Avg | -0.3722±1.3365 | 2.1403±1.7549 | 1.0447±1.005 | 0.5820±1.071 |  | NA |
| Stat R-S | Avg | t=0.9647, p=0.3554 | t=4.2249, p=0.0014* | t=3.6010, p=0.0042* | t=1.8825, p=0.0865 |  |  |
| Stat R-S | Diff | No | Yes | Yes | No |  |  |
| Left≠Right | AMG | t=17.21, p=0.0034 | t=11.69, p=0.0072 | t=4.164, p=0.0531 | t=3.343, p=0.0790 |  |  |
| Left≠Right | AMG | Yes | Yes | No | No |  |  |

**Table.** Difference table for [Female] – [Male], where positive numbers indicate female greater and negative numbers indicate male greater.

Table S23 | [Female] – [Male] predominance sided average for Sad

| Side | Area | Sad-org | Sad-8nb | Sad-32nb | Sad-64nb |  |
| --- | --- | --- | --- | --- | --- | --- |
| Left-Sad |  |  |  |  |  |  |
|  | AMG-LB | Female | Female | Female | Male |  |
|  | AMG-CM | Female | Female | Female | Female |  |
|  | AMG-SP | Female | Female | Female | Female |  |
| Right-Sad |  |  |  |  |  |  |
|  | AMG-LB | Male | Female | Female | Female |  |
|  | AMG-CM | Male | Female | Female | Female |  |
|  | AMG-SP | Male | Female | Female | Female |  |

Table S24 | Average Female and Male amygdala sad responses

|  | Area | SadOrg | Sad-8nb | Sad-32nb | Sad-64nb | Decomposition | Sig |
| --- | --- | --- | --- | --- | --- | --- | --- |
| Male |  |  |  |  |  |  |  |
|  | Left |  |  |  |  |  |  |
|  | LB | -0.0717±0.6778 | 0.2977±0.6889 | -0.3427±0.6388 | -0.5983±0.6455 | df=3,44, F=4.015, p=0.0131 | Yes |
|  | CM | 0.0326±0.8029 | 0.2857±0.6736 | -0.2013±0.7302 | -0.7074±0.5869 | df=3,44, F=4.344, p=0.0091 | Yes |
|  | SP | -0.1701±0.8466 | 0.2716±0.7172 | -0.2054±0.6637 | -0.7221±0.6975 | df=3,44, F=3.675, p=0.0190 | Yes |
|  | Avg | -0.0697±0.7758 | 0.2850±0.6832 | -0.2498±0.6776 | -0.6759±0.6433 | t=0.6828, p=0.5437 | No |
|  | Stat | t=0.3112, p=0.7614 | t=1.4451, p=0.1763 | t=1.2771, p=0.2279 | t=3.6396, p=0.0039* |  |  |
|  |  | No | No | No | Yes |  |  |
|  | Right |  |  |  |  |  |  |
|  | LB | 1.7170±1.1212 | 0.6405±0.8603 | 0.2400±0.8086 | -0.1868±0.9641 | df=3,44, F=8.928, p<0.0001 | Yes |
|  | CM | 1.4726±1.1613 | 0.5043±0.8603 | 0.2323±0.8296 | -0.4577±0.9277 | df=3,44, F=8.442, p=0.0002 | Yes |
|  | SP | 1.3502±1.1653 | 0.4560±0.8645 | 0.1998±0.8013 | -0.5251±0.9202 | df=3,44, F=8.006, p=0.0002 | Yes |
|  | Avg | 1.5133±1.1493 | 0.5336±0.8617 | 0.2240±0.8132 | -0.3899±0.9373 | t=1.141, p=0.3216 | No |
|  | Stat | t=4.4512, p=0.0008* | t=2.1451, p=0.0551 | t=0.9542, p=0.3605 | t=1.4410, p=0.1774 |  |  |
|  |  | Yes | No | No | No |  |  |
| Female |  |  |  |  |  |  |  |
|  | Left |  |  |  |  |  |  |
|  | LB | 0.5388±0.6249 | 0.6894±0.6061 | 0.8435±0.7366 | -0.4639±0.6000 | df=3,44, F=10.08, p<0.0001 | Yes |
|  | CM | 0.6964±0.4811 | 0.8797±0.5995 | 0.9960±0.7697 | -0.2318±0.6043 | df=3,44, F=9.665, p<0.0001 | Yes |
|  | SP | 0.6456±0.543 | 0.8668±0.6375 | 0.9036±0.7621 | -0.3446±0.6957 | df=3,44, F=9.338, p<0.0001 | Yes |
|  | Avg | 0.6269±0.5497 | 0.8120±0.6144 | 0.9144±0.7561 | -0.3468±0.6333 | t=1.7357, p=0.1810 | No |
|  | Stat | t=3.9506, p=0.0023 | t=4.5782, p=0.0008* | t=4.1894, p=0.0015* | t=1.8970, p=0.0844 |  |  |
|  |  | Yes | Yes | Yes | No |  |  |
|  | Right |  |  |  |  |  |  |
|  | LB | 0.7028±0.4350 | 2.4422±1.4827 | 1.2308±0.8216 | 0.3590±0.74432 | df=3,44, F=11.05, p<0.0001 | Yes |
|  | CM | 0.7887±0.3690 | 2.8690±1.4441 | 1.3424±0.6528 | 0.2724±0.7411 | df=3,44, F=18.92, p<0.0001 | Yes |
|  | SP | 0.7328±0.4140 | 2.5145±1.5125 | 1.1678±0.7611 | 0.3027±0.7334 | df=3,44, F=12.31, p<0.0001 | Yes |
|  | Avg | 0.7414±0.4060 | 2.6086±1.4798 | 1.2470±0.7452 | 0.3114±0.7396 | t=2.4610, p=0.0908 | No |
|  | Stat | t=6.3258, p<0.0001* | t=6.1065, p<0.0001* | t=5.7967, p<0.0001* | t=1.4585, p=0.1727 |  |  |
|  |  | Yes | Yes | Yes | No |  |  |

### Absolute [Female] – [Male] sexual dimorphism for activation and deactivation profiles

The absolute difference between female and males for activation and deactivation resulted in several significant findings (Table S25 | [Female] average happy and sad by activation and deactivation (bilateral presentation), Table S26 | [Male] average happy and sad by activation and deactivation (bilateral presentation), and Table S27 | [Female] – [Male] average happy and sad by activation and deactivation (bilateral presentation) with sided predominance). An ANOVA of row (i.e. subdivision) by column (i.e. decomposition) for female different than male for **activation** response found row df=3,88, F=0.1636, p=0.6868, column df=3,88, F=4.471, p=0.0057, and interaction of row by column F=0.7801, p=0.5082. An ANOVA of row (i.e. subdivision) by column (i.e. decomposition) for female different than male for **deactivation** response found row df=3,88, F=0.6352, p=0.4276, column df=3,88, F=0.9031, p=0.4430, interaction of row by column F=0.1162, p=0.9504. A follow-up found the absolute [Female] – [Male] average for activation decomposition was significant for sad (df=3,44, F=3.403, p=0.0257), but not for happy. For absolute [Female] difference for bilateral presentations, no significant activation or deactivation was found as a function of bilateral presentation. For Absolute [Male] difference for bilateral presentations, the response to happy by decomposition was significant for activation (df=3,44, F=6.359, p=0.0011) and deactivation (df=3,44, F=3.131, p=0.0350). Here the sexual dimorphism, was an absolute male difference for activation and deactivation as a response to happy, with female different than male for by activation as a function of decomposition for sad only.

Table S25 | [Female] average happy and sad by activation and deactivation (bilateral presentation)

|  |  | Org | 8nb | 32nb | 64nb | Decomposition | Sig |
| --- | --- | --- | --- | --- | --- | --- | --- |
| Activation |  |  |  |  |  |  |  |
|  | Happy | -0.0332±0.5989 (0.0182) | -0.1525±0.4748 (0.0148) | 0.2186±0.8266 (0.0226) | 0.2107±0.5159 (0.0248) | df=3,44, F=1.061, p=0.3753 | No |
|  | Sad | -0.1436±0.6028 (0.0162) | 0.8669±1.6361 (0.0432) | -0.1833±1.0254 (0.0269) | 0.2788±0.5840 (0.0196) | df=3,44, F=2.584, p=0.0652 | No |
| Deactivation |  |  |  |  |  |  |  |
|  | Happy | 0.0317±0.4862 (0.0180) | 0.0237±0.6325 (0.0210) | 0.2094±0.3935 (0.0204) | 0.4129±0.7422 (0.0202) | df=3,44, F=1.205, p=0.3191 | No |
|  | Sad | 0.1130±0.3628 (0.0243) | 0.1865±0.2174 (0.0185) | 0.0405±0.4336 (0.0302) | 0.3169±0.5195 (0.0162 | df=3,44, F=1.049, p=0.3805 | No |

Table S26 | [Male] average happy and sad by activation and deactivation (bilateral presentation)

|  |  | Org | 8nb | 32nb | 64nb | Decomposition | Sig |
| --- | --- | --- | --- | --- | --- | --- | --- |
| Activation |  |  |  |  |  |  |  |
|  | Happy | 0.2225±0.8622 (0.0295) | -0.7740±0.8927 (0.0288) | 0.2338±0.5796 (0.0205) | 0.2961±0.3369 (0.0226) | df=3,44, F=6.359, p=0.0011 | Yes |
|  | Sad | 0.8092±1.0231 (0.0364) | 0.0839±0.6820 (0.0245) | 0.2325±0.6004 (0.0240) | 0.3269±0.5688 (0.0308) | df=3,44, F=2.152, p=0.1073 | No |
| Deactivation |  |  |  |  |  |  |  |
|  | Happy | 0.0843±0.5289 (0.0398) | -0.1879±0.2295 (0.0209) | 0.1593±0.4311 (0.0179) | 0.4559±0.7456 (0.0245) | df=3,44, F=3.131, p=0.0350 | Yes |
|  | Sad | 0.4769±0.5511 (0.0243) | 0.1230±0.5013 (0.0248) | 0.2312±0.4586 (0.0173) | 0.2333±0.7242 (0.0244) | df=3,44, F=0.8337, p=0.4826 | No |

Table S27 | [Female] – [Male] average happy and sad by activation and deactivation (bilateral presentation) with sided predominance

|  |  | Org | 8nb | 32nb | 64nb | Decomposition |
| --- | --- | --- | --- | --- | --- | --- |
| Activation |  |  |  |  |  |  |
|  | Happy | -0.1870±0.8985 (0.0258) | 0.4717±1.0275 (0.0292) | 0.0768±0.8810 (0.0237) | 0.0451±0.5458 (0.0228) | df=3,44, F=1.221, p=0.2488 |
|  | Sad | -0.6016±1.0433 (0.0280) | 0.8170±1.611 (0.0425) | -0.2835±1.1199 (0.0294) | 0.1395±0.6039 (0.0193) | df=3,44, F=3.403, p=0.0257* (Yes) |
| Deactivation |  |  |  |  |  |  |
|  | Happy | -0.3266±0.5731 (0.0221) | -0.0459±0.4713 (0.0204) | -0.1919±0.4972 (0.0175) | 0.0903±0.7998 (0.0220) | df=3,44, F=1.086, p=0.2718 |
|  | Sad | -0.1177±0.6958 (0.0226) | 0.0140±0.7306 (0.0218) | -0.0174±0.4910 (0.0172) | 0.0950±1.0464 (0.0278) | df=3,44, F=0.1577, p=0.1162 |
| Activation |  |  |  |  |  |  |
|  | Happy | Male | Female | Female | Female |  |
|  | Sad | Male | Female | Male | Female |  |
| Deactivation |  |  |  |  |  |  |
|  | Happy | Male | Male | Male | Female |  |
|  | Sad | Male | Female | Male | Female |  |
| Activation |  |  |  |  |  |  |
|  | Happy | NA | NA | NA | NA |  |
|  | Sad | t=1.9975, p=0.0711 | t=1.7568, p=0.1067 | t=0.8769, p=0.3993 | t=0.8002, p=0.4405 |  |
| Deactivation |  |  |  |  |  |  |
|  | Happy | NA | NA | NA | NA |  |
|  | Sad | NA | NA | NA | NA |  |

**Table.** The decomposition by emotional resolvability for Sad was significant, but not for happy, concerning activation series, while no series was significant for deactivation. Follow-up failed to find any differences.

Fig. S9 | Absolute difference [Female] – [Male] by amygdala subdivisions (bilateral presentation)


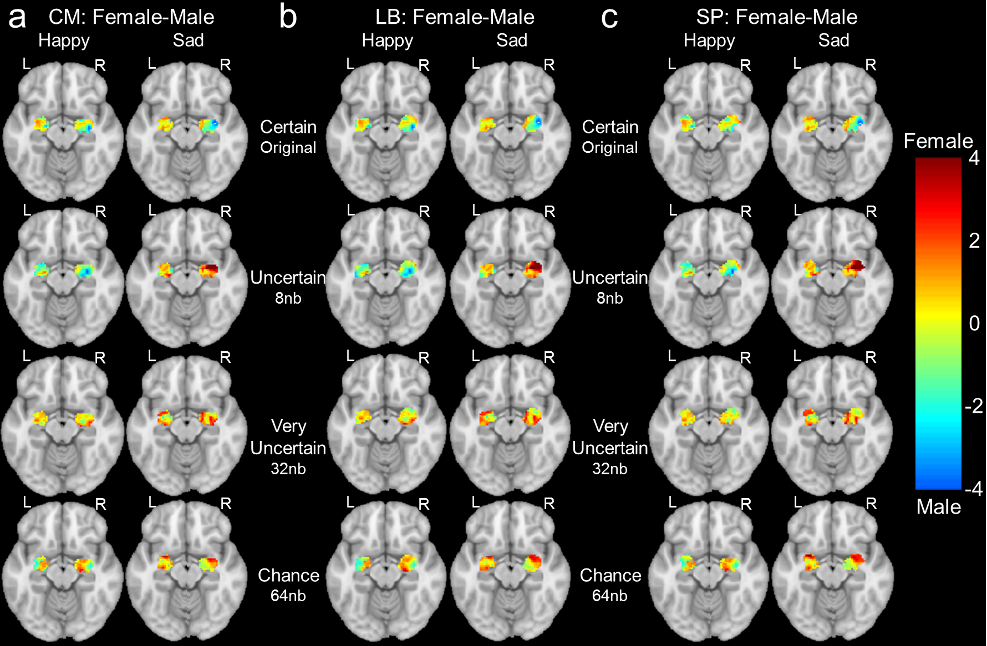


**Figure.** Sexual differences between [Female] – [Male] amygdala subdivision activation or deactivation to emotional resolvability. Each column (a), (b) and (c) contains Happy (left side column) and Sad (right side column) for both left and right hemispheric presentations of the brain.

Fig. S10 | Activation and deactivation profiles for entire amygdala in female and male separately (bilateral presentation)


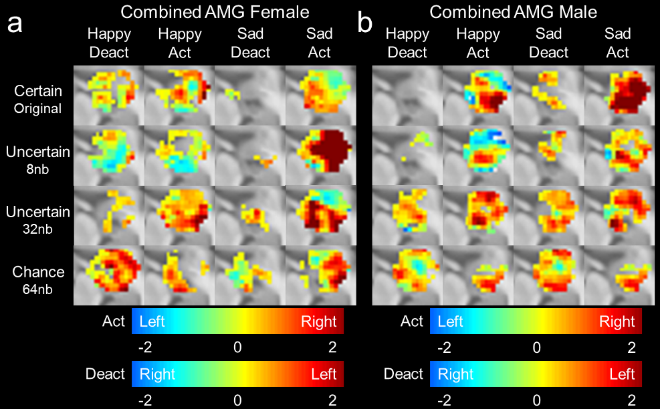


**Figure.** Activation and deactivation profiles for entire amygdala in female (a) and male (b) for bilateral presentations for emotional resolvability. By column, happy deactivation, happy activation, sad deactivation, and sad activation, by row certain stimuli in the un-altered original format, followed by uncertain 8nb, and 32nb, and chance identifiable 64nb. Colorbars are for activation and deactivation separately, where activation negative corresponds to a left-sided laterality and positive to a right-sided laterality, and where deactivation negative corresponds to a right-sided laterality and positive to a left-sided laterality.

Fig. S11 | [Female] – [Male] average response happy and sad by amygdala activation and deactivation (bilateral presentation)


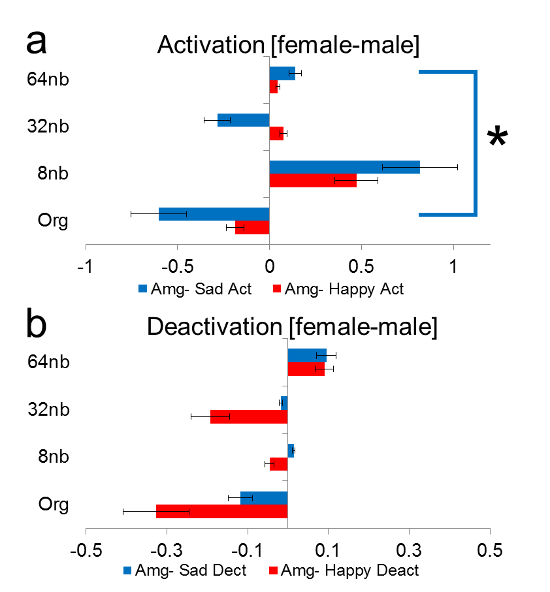


**Figure.** The respective [Female] – [Male] average amygdala t-values for happy (red) and sad (blue) by activation (a) and deactivation (b) for bilateral presentation. Top barplot activation, bottom deactivation, with uncertain emotion on the y axis (Org, 8nb, 32nb, 64nb).

# Silent analysis negative control experiments of baseline and sparse sampling

The present study determined the functional response to certain and uncertain emotional signals in sound utilizing a sparse sampling fMRI paradigm of uncertain stimuli blocks interspersed with silence. We expected two outcomes: 1) covarying FIS or ENV in emotion stimuli would reveal which cue aides in resolvability and 2) the functional region responsible for emotion resolvability would vary differently with certain to uncertain emotional stimuli. Throughout the sparse sampling experiment, silent stimuli (Fig. S2 | Silent stimuli used for sparse sampling) were collected as negative control. In the general linear model (GLM), silent blocks were used as the comparative category control. The silent block analysis experiment was conducted as a negative control (no stimuli effect during silence), to determine the robustness of the present findings concerning three aspects: 1) activation/deactivation of ROI in question, 2) hemispheric lateralization, and 3) modulation to emotional resolvability. Silent periods were aggregations of 3 or 4 silent stimuli per block with 34 silent periods per run, two runs for each individual (n=15), processed in the same manner as the experimental design (Fig. 1 in manuscript). We first assessed whether any discernable activation/deactivation was present during our silent periods by aggregating all silent periods from all blocks (Fig. S1 | Experimental design, sparse sampling paradigm parameters utilized to optimize, run format, analysis of explanatory variables). Then we determined whether the GLM model we used for assessing our uncertain emotional stimuli would elicit results when the model was used for silence.

For HG considerable deactivation was apparent for all subdivisions, with no activation noteworthy. For AMG, activation and deactivation circumscribing zero were both apparent, which could be due to the time-delayed response pattern for the amygdala interacting with other regions (as seen in electrophysiology with rats; Nakao et al., 2004) from previous stimuli blocks. Nevertheless, for HG significant deactivation was apparent and for AMG no significant difference in activation/deactivation during silence (*p* < 0.05). To determine baseline properties of the amygdala and HG to serve as a negative control experiment, voxels were coded based on a model of stimuli which did not exist (i.e. silent modeling; Fig. S1 | Experimental design, sparse sampling paradigm parameters utilized to optimize, run format, analysis of explanatory variables) to determine residual contributions to emotional resolvability. Volume averages were grouped in a pattern resembling the identical model used within the emotional resolvability experiment.

## Heschl’s gyrus silent analysis during complete silence and interleaved sparse sampling

Two negative control experiments were conducted to determine HG baseline properties. No significant HG activation in complete silence (a and b) nor in silent interleaved volumes (c) acquired during the experiment was observed (Fig. S12 | Heschl’s gyrus complete silence and interleaved sparse sampling). Average t-values for complete silence (d) and interleaved silence during sparse sampling (e) demonstrated significant deactivation (Fig. S12 | Heschl’s gyrus complete silence and interleaved sparse sampling). Baseline activation of HG during complete silence was not modulated over the experimental run (Table S28 | Heschl’s gyrus negative control experiments: Complete silence) nor was modulation found during the interleaved blocks of silence collected during the experimental run (Table S29 | Heschl’s gyrus negative control experiments: Interleaved sparse sampling average by sequence in time), as indicated by follow-up analysis. The sole significant t-value (difference by block even by odd, four t=3.3964, p=0.0043) can be explained by the slight, but non-significant hemispheric lateralization of HG during the interleaved silence (Table S30 | Heschl’s gyrus negative control experiments: Interleaved sparse sampling by block of stimuli (Block 1 sequences) and Table S31 | Heschl’s gyrus negative control experiments: Interleaved sparse sampling by block of stimuli (Block 2 sequences)). As illustrated by the previous tables, these values were non-significant when grouped as in the experimental model or when grouped by a time-series block model (Fig. 6 in manuscript). In summary, no difference for complete silence or interleaved silence for HG was found, and was within normal baseline ranges of activation and deactivation (Fig. 6 in manuscript).

Fig. S12 | Heschl’s gyrus complete silence and interleaved sparse sampling


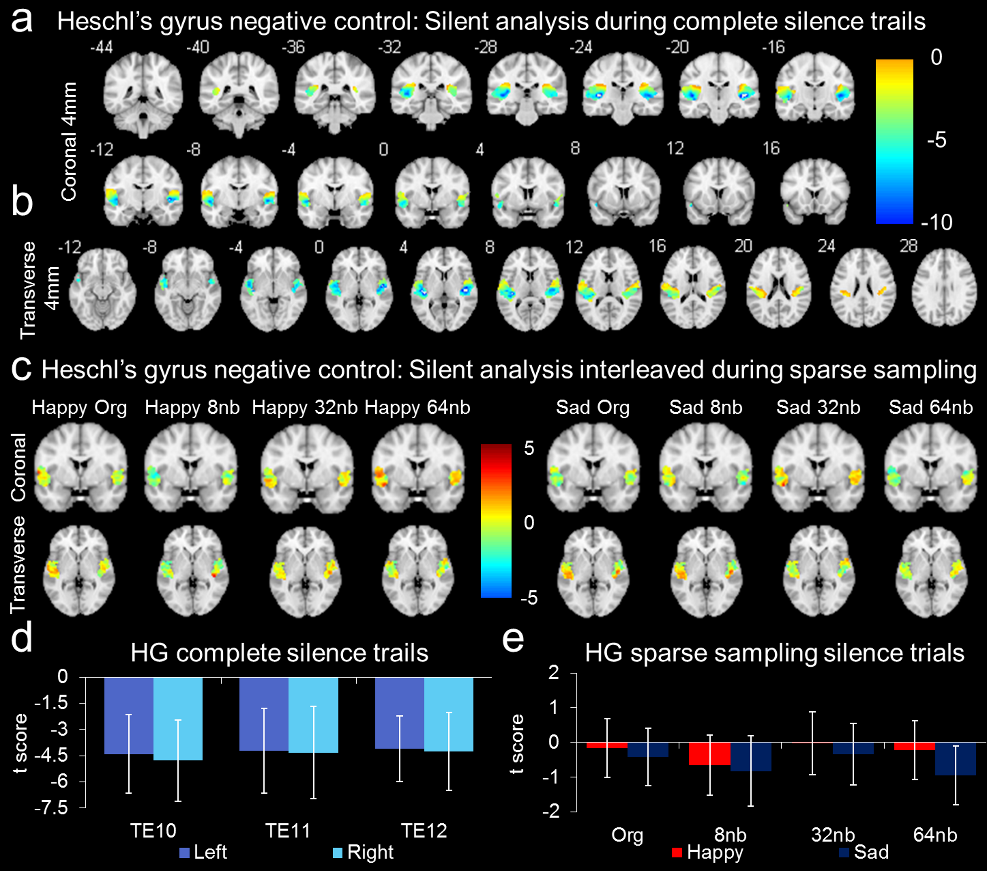


**Figure.** Heschl’s gyrus complete silence and interleaved sparse sampling of silence. Heschl’s gyrus negative control during complete silence trials, a visualization of coronal (a) and transverse (b) sections from the complete averaged blocks of silence. Heschl’s gyrus negative control during interleaved silence sparse sampling trials (c) left for blocks occurring adjacent to happy original or its decompositions and right for blocks occurring adjacent to sad original or its decompositions. The average t-value scores from the extracted silent volumes from complete silence trials (d) and interleaved during sparse sampling trials (e). Complete silence did not modulate HG (Table S28 | Heschl’s gyrus negative control experiments: Complete silence) nor did interleaved silence result in HG modulation (Table S29 | Heschl’s gyrus negative control experiments: Interleaved sparse sampling average by sequence in time). Baseline was changed between complete silence and interleaved silence.

Table S28 | Heschl’s gyrus negative control experiments: Complete silence

|  |  | Avg ± SD (SEM) | Lateralization | Yes/No |
| --- | --- | --- | --- | --- |
| Left |  |  |  |  |
|  | Te1.0 | -4.3980±2.2580 (0.0551) | t=0.6427, p=0.5308 | No |
|  | Te1.1 | -4.2239±2.4300 (0.0652) | t=0.1410, p=0.8899 | No |
|  | Te1.2 | -4.0974±1.8825 (0.0533) | t=0.3008, p=0.7680 | No |
| Avg-Left |  | -4.2397± 2.1901 | t=0.3531, p=0.7293 | No |
| Right |  |  |  |  |
|  | Te1.0 | -4.7841±2.3267 (0.0652) | t=0.6427, p=0.5308 | No |
|  | Te1.1 | -4.3197±2.6311 (0.0785) | t=0.1410, p=0.8899 | No |
|  | Te1.2 | -4.2710±2.2352 (0.0845) | t=0.3008, p=0.7680 | No |
| Avg-Right |  | -4.4583±2.3977 | t=0.3531, p=0.7293 | No |

No difference in HG left/right lateralization df=4, t=1.1792, p=0.3037. For all statistics within the table, df=1,15.

Table S29 | Heschl’s gyrus negative control experiments: Interleaved sparse sampling average by sequence in time

| Sequence | Block (even)  Avg ± SD (SEM) | Block (odd)  Avg ± SD (SEM) | Difference  (t-test) | Yes/No |
| --- | --- | --- | --- | --- |
| One | -0.1659±0.8486 (0.0123) | -0.4131±0.8303 (0.0121) | t=1.1531, p=0.2682 | No |
| Two | -0.6509±0.8728 (0.0127) | -0.8216±1.0098 (0.0147) | t=0.6547, p=0.5233 | No |
| Three | -0.0246±0.9089 (0.0132) | -0.3410±0.8825 (0.0128) | t=1.3886, p=0.1867 | No |
| Four | -0.2116±0.8468 (0.0123) | -0.9514±0.8436 (0.0122) | t=3.3964, p=0.0043 | Yes* |
| Avg | -0.2633±0.8693 | -0.6318±0.8916 | t=1.6418, p=0.1229 | No |

We would assume washout effect from the paradigm would be close to 0-activation carrying over to next block, any residual would be baseline hemispheric lateralization. You can test this by conducting an emotion by interleaved t-test for emotion difference. * Statistically significant, but negative deactivation, may be related to baseline drift. Follow-up: t=2.3971, p=0.0234, df=28. Because both values are within SD of range of values, most likely rsfMRI modulatory response of HRF, otherwise would be a difference for each category. Further, no lateralization was found at the subnuclei level.

Table S30 | Heschl’s gyrus negative control experiments: Interleaved sparse sampling by block of stimuli (Block 1 sequences)

| Sequence | Block (Even)  Left Hemi | Block (Even)  Right Hemi | Block (odd)  Left Hemi | Block (Odd)  Right Hemi |
| --- | --- | --- | --- | --- |
| One | -0.1822±0.8077 (0.0154) | -0.1430±0.9025 (0.0203) | -0.2740±0.8709 (0.0166) | -0.6079±0.7266 (0.0163) |
| Two | -0.9353±0.7771 (0.0148) | -0.2519±0.8431 (0.0190) | -0.5178±0.9437 (0.0179) | -1.2474±0.9435 (0.0212) |
| Three | -0.3889±0.8094 (0.0154) | 0.4840±0.7880 (0.0177) | -0.3746±0.8518 (0.0162) | -0.2950±0.9209 (0.0205) |
| Four | -0.1870±0.9024 (0.0172) | -0.2453±0.7633 (0.0170) | -1.2839±0.6817 (0.0130) | -0.4891±0.8292 (0.0186) |
| Avg | -0.4233±0.8242 | -0.0391±0.8242 | -0.6126±0.8370 | -0.6599±0.8551 |
| Stat | t=2.3875, p=0.0970 | t=0.2217, p=0.8388 | t=2.6715, p=0.0756 | t=3.202, p=0.0493 |
| L≠R | t=1.2766, p=0.2122 | t=1.2766, p=0.2122 | t=0.1531, p=0.8794 | t=0.1531, p=0.8794 |
| E-L≠O-L | t=0.6241, p=0.5376 | NA | t=0.6241, p=0.5376 | NA |
| E-R≠O-R | NA | t=2.0245, p=0.0526 | NA | t=2.0245, p=0.0526* |

E – even, O - odd, L - left, R – right, * = p <0.05, when rounded is non-significant.

Table S31 | Heschl’s gyrus negative control experiments: Interleaved sparse sampling by block of stimuli (Block 2 sequences)

| Sequence | Block (Even)  Left Hemi | Block (Even)  Right Hemi | Block (odd)  Left Hemi | Block (Odd)  Right Hemi |
| --- | --- | --- | --- | --- |
| One | -0.0762±0.6257 (0.0119) | -0.7797±0.7671 (0.0170) | 0.5213±0.4759 (0.0090) | 0.4323±0.5879 (0.0130) |
| Two | -0.3266±0.6002 (0.0114) | 0.1061±0.6706 (0.0150) | 0.2722±0.7712 (0.0146) | -0.4912±0.7378 (0.0165) |
| Three | -0.6359±0.8843 (0.0168) | 0.3794±0.7554 (0.0168) | 0.6496±0.8013 (0.0152) | 0.4873±0.7693 (0.0170) |
| Four | 0.4743±0.7742 (0.0147) | 0.9239±0.6878 (0.0152) | -0.7798±0.6088 (0.0116) | 0.3352±0.8081 (0.0181) |
| Avg | 0.1411±0.7211 | 0.1574±0.7202 | 0.1658±0.6643 | 0.1909±0.7258 |
| Stat | t=0.6007, p=0.5904 | t=0.4427, p=0.6880 | t=0.5105, p=0.6449 | t=0.8317, p=0.4666 |
| L≠R | t=0.0619, p=0.9510 | t=0.0619, p=0.9510 | t=0.0988, p=0.9220 | t=0.0988, p=0.9220 |
| E-L≠O-L | t=0.0976, p=0.9230 | NA | t=0.0976, p=0.9230 | NA |
| E-R≠O-R | NA | t=0.1269, p=0.8999 | NA | t=0.1269, p=0.8999 |

E – even, O - odd, L - left, R – right.

## Amygdala gyrus silent analysis during complete silence and interleaved sparse sampling

Two negative control experiments were conducted to determine amygdala baseline properties. No significant amygdala activation in complete silence (a and b) nor in silent interleaved volumes (c) acquired during the experiment was observed (Fig. S13 | Amygdala complete silence and interleaved sparse sampling). Average t-values for complete silence (d) and interleaved silence during sparse sampling (e) demonstrated significant deactivation (Fig. S13 | Amygdala complete silence and interleaved sparse sampling). Baseline activation of amygdala during complete silence was not modulated over the experimental run (Table S32 | Amygdala negative control experiments: Complete silence) nor was modulation found during the interleaved blocks of silence collected during the experimental run (Table S33 | Amygdala negative control experiments: Interleaved sparse sampling average by sequence in time). However, significant hemispheric lateralization was observed in complete silence (Fig. S13 | Amygdala complete silence and interleaved sparse sampling, d; lateralization in Table S32 | Amygdala negative control experiments: Complete silence). When we analyzed the hemispheric lateralization interleaved during sparse sampling, it varied and was not as consistent as during complete silence (i.e. Block 1 (Even) Right Hemi t=3.4484, p=0.0018). Follow-up analysis found even and odd block differences for hemispheric lateralization, but the trend was not consistent (Table S34 | Amygdala negative control experiments: Interleaved sparse sampling by block of stimuli (Block 1 sequences) and Table S35 | Amygdala negative control experiments: Interleaved sparse sampling by block of stimuli (Block 2 sequences)). The carry-over effect to the experimental design was that baseline hemispheric lateralization was slightly significant as was in the complete silence analysis, with partial effects found during silent modeling of the interleaved sparse sampling blocks. The follow-up analysis failed to find activation deactivation patterns which contributed to the hemispheric lateralization (Fig. 6 in manuscript), that is no difference in activation or deactivation contributed to leaning of the amygdala of one hemisphere or another. In summary, no difference for complete silence or interleaved silence for amygdala was found as a function of baseline modulation as a function of sparse sampling; however, interestingly, hemispheric lateralization was apparent and significantly contributed to baseline properties in the amygdala.

Fig. S13 | Amygdala complete silence and interleaved sparse sampling


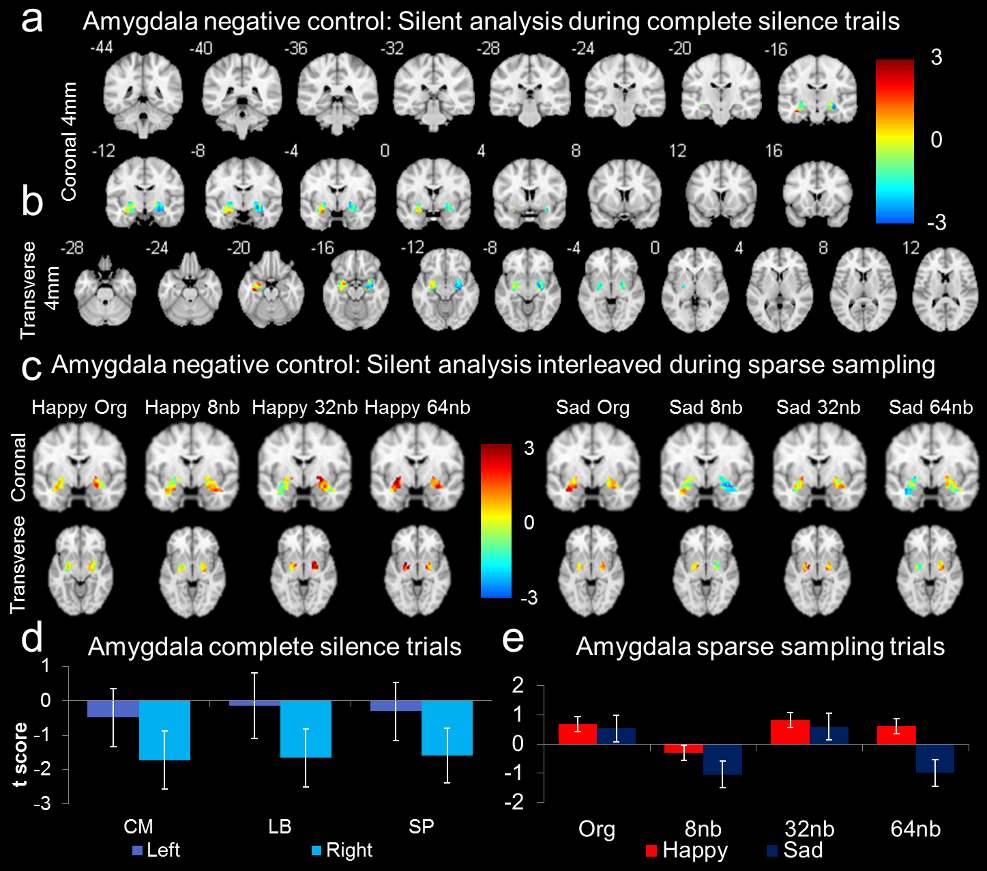


**Figure.** Amygdala complete silence and interleaved sparse sampling of silence. Amygdala negative control during complete silence trials, a visualization of coronal (a) and transverse (b) sections from the complete averaged blocks of silence. Amygdala negative control during interleaved silence sparse sampling trials (c) left for blocks occurring adjacent to happy original or its decompositions and right for blocks occurring adjacent to sad original or its decompositions. The average t-value scores from the extracted silence volumes from complete silence trials (d) and interleaved during sparse sampling trials (e). Complete silence did not modulate HG (Table S32 | Amygdala negative control experiments: Complete silence) nor did interleaved silence result in HG modulation (Table S33 | Amygdala negative control experiments: Interleaved sparse sampling average by sequence in time). However, two interesting observations: 1) hemispheric lateralization persisted during complete silence, and 2) hemispheric lateralization persisted during interleaved sparse sampling of silence, but was adjusted in a similar manner as was HG baseline modulation during interleaved sparse sampling.

Table S32 | Amygdala negative control experiments: Complete silence

|  |  | Avg ± SD (SEM) | Lateralization | Yes/No |
| --- | --- | --- | --- | --- |
| Left |  |  |  |  |
|  | CM | -0.4899±0.8496(0.0394) | t=5.6830, p<0.0001 | Yes |
|  | LB | -0.1432±0.8483(0.0395) | t=6.1346, p<0.0001 | Yes |
|  | SP | -0.3075±0.7974(0.0370) | t=5.8456, p<0.0001 | Yes |
| Avg-Left |  | -0.3135±(0.8318) | t=5.8985, p<0.0001 | Yes |
| Right |  |  |  |  |
|  | CM | -1.7279±0.8437(0.0403) | t=5.6830, p<0.0001 | Yes |
|  | LB | -1.6622±0.9590 (0.0483) | t=6.1346, p<0.0001 | Yes |
|  | SP | -1.5874±0.8480 (0.0412) | t=5.8456, p<0.0001 | Yes |
| Avg-Right |  | -1.6592±(0.8836) | t=5.8985, p<0.0001 | Yes |

**Table.** Significant difference in amygdala left/right lateralization df=4, t=15.3746, p<0.0001. For all statistics within the table, df=1,15. Consistent right greater left hemisphere deactivation in complete silence negative control. Here baseline hemispheric lateralization was prominent.

Table S33 | Amygdala negative control experiments: Interleaved sparse sampling average by sequence in time

| Sequence | Block (even)  Avg ± SD (SEM) | Block (odd)  Avg ± SD (SEM) | Difference  (t-test) | Yes/No |
| --- | --- | --- | --- | --- |
| One | 0.6937±0.7786 (0.0188) | 0.5378±0.7811 (0.0188) | t=0.7730, p=0.4524 | No |
| Two | -0.2983±0.7612 (0.0184) | -1.0433±1.0506 (0.0253) | t=2.7464, p=0.0158 | Yes |
| Three | 0.8266±1.1117 (0.0269) | 0.5936±0.9394 (0.0265) | t=0.9606, p=0.3530 | No |
| Four | 0.6159±1.0121 (0.0286) | -0.9787±0.7796 (0.0202) | t=7.9218, p<0.0001 | Yes |
| Avg | 0.4595±0.9159 | -0.2227±0.8877 | t=2.9764, p=0.0100 | Yes |

**Table.** We would assume washout effect from the paradigm would be close to 0-activation carrying over to next block, any residual would be baseline hemispheric lateralization. You can test this by conducting an emotion by interleaved t-test for emotion difference.

Table S34 | Amygdala negative control experiments: Interleaved sparse sampling by block of stimuli (Block 1 sequences)

| Sequence | Block (Even)  Left Hemi | Block (Even)  Right Hemi | Block (odd)  Left Hemi | Block (Odd)  Right Hemi |
| --- | --- | --- | --- | --- |
| One | 0.3586±0.6071 (0.0203) | 1.0590±0.7809 (0.0272) | 0.6580±0.8276 (0.0276) | 0.4066±0.7043 (0.0246) |
| Two | -0.5354±0.7177 (0.0240) | -0.040±0.7226 (0.0252) | -0.3495±0.8277 (0.0276) | -1.8003±0.6799 (0.0237) |
| Three | 0.3729±1.0189 (0.0341) | 1.3181±0.9931 (0.0346) | 0.3577±0.9020 (0.035) | 0.8586±0.9102 (0.0374) |
| Four | 0.6671±0.9658 (0.0375) | 0.5584±1.0597 (0.0436) | -1.1653±0.7031 (0.0251) | -0.7712±0.8079 (0.0304) |
| Avg | 0.2158±0.8274 | 0.7239±0.8891 | -0.1248±0.8151 | -0.3266±0.7756 |
| Stat | t=0.8291, p=0.4679 | t=2.4171, p=0.0944 | t=0.3073, p=0.7787 | t=0.5448, p=0.6238 |
| L≠R | t=1.6203, p=0.1164 | t=1.6203, p=0.1164 | t=0.6946, p=0.4930 | t=0.6946, p=0.4930 |
| E-L≠O-L | t=1.1358, p=0.2657 | NA | t=1.1358, p=0.2657 | NA |
| E-R≠O-R | NA | t=3.4484, p=0.0018* | NA | t=3.4484, p=0.0018* |

**Table.** We would assume washout effect from the paradigm would be close to 0-activation carrying over to next block, any residual would be baseline hemispheric lateralization. You can test this by conducting an emotion by interleaved t-test for emotion difference. E – even, O - odd, L - left, R – right, * = p <0.05.

Table S35 | Amygdala negative control experiments: Interleaved sparse sampling by block of stimuli (Block 2 sequences)

| Sequence | Block (Even)  Left Hemi | Block (Even)  Right Hemi | Block (odd)  Left Hemi | Block (Odd)  Right Hemi |
| --- | --- | --- | --- | --- |
| One | -0.2103±0.6345 (0.0247) | 0.2346±0.6312 (0.027) | 0.5076±0.8123 (0.0317) | 0.7275±0.4761 (0.0204) |
| Two | 0.0405±0.7317 (0.0242) | 0.4979±0.6146 (0.0219) | -0.0604±0.8051 (0.0263) | -1.0602±0.9304 (0.0327) |
| Three | -0.3342±0.9091 (0.0301) | 0.8091±0.8866 (0.0318) | 0.1178±0.9336 (0.0364) | 0.3536±0.7090 (0.0303) |
| Four | 1.3888±0.9271 (0.0361) | 1.0265±0.8556 (0.0366) | -0.9831±0.8254 (0.0274) | -0.0551±0.7156 (0.0257) |
| Avg | 0.2212±0.8006 | 0.6420±0.7470 | -0.1045±0.8441 | -0.0086±0.7078 |
| Stat | t=0.5573, p=0.6162 | t=3.6939, p=0.0344* | t=0.3308, p=0.7625 | t=0.0222, p=0.9837 |
| L≠R | t=1.4884, p=0.1478 | t=1.4884, p=0.1478 | t=0.3372, p=0.7385 | t=0.3372, p=0.7385 |
| E-L≠O-L | t=1.0843, p=0.2875 | NA | t=1.0843, p=0.2875 | NA |
| E-R≠O-R | NA | t=2.4486, p=0.0209* | NA | t=2.4486, p=0.0209* |

We would assume washout effect from the paradigm would be close to 0-activation carrying over to next block, any residual would be baseline hemispheric lateralization. You can test this by conducting an emotion by interleaved t-test for emotion difference. E – even, O - odd, L - left, R – right, * = p <0.05.

# The effect size of emotion

## Effect sizes for emotion and power analysis

We report effect sizes in line the American Psychological Association (APA) guidelines on statistical reporting (Wilkinson and APA Task Force on Statistical Inference, 1999). As recommended (Friston, 2012), we searched the literature of functional meta-analysis concerning emotion to discern a pre-experiment effect size of emotion (Witteman et al., 2012; Wager et al., 2003; Phan et al., 2002; Vigneau et al., 2006; Schirmer et al., 2012) to bolster our statistics against spurious and ‘trivial ’correlations (Eklund, Nichols, Knutsson, 2016; Friston, et al., 2009; Fisher and Student, 2012; Genovese, et al., 2002); however, averages were not readily discernable for effect size statistics. Therefore, we derived effect size statistics from the reviews by Koelsch (2005; 2010; 2014) for emotion in music; but, only for functional studies with psychophysics (Brattico et al., 2011; Kawakami et al., 2013; Khalfa et al., 2005; Khalfa et al., 2008; Mitterschiffthaler et al., 2007; Park et al., 2015).

The effect size *d* was defined as the difference between the group means, $X$1 - $X$2, divided by standard deviation from the mean (σ) of either group (Cohen, 1988; Cohen, 1992). For the present study we would take the mean between certain and uncertain emotion by hemispheric lateralization, expressed as t-value for the ROI under investigation. The standard deviation, σ was a function of $\left( X-X \right)^{2}$ where the $X$ minus $X$ was a subtraction of the mean from every value in your dataset squared, and *N* the number of data values in your dataset.

Equation S4 | Cohen’s *d* calculation for effect size

$$d=\frac{X1-X2}{\sigma}$$

Equation S5 | The σ for effect size determination

$$\sigma=\sqrt{\frac{\sum\left( X-X \right)^{2}}{N}}$$

To incorporate the effect size (ES) calculation of emotion from the meta-analyses (Witteman et al., 2012; Wager et al., 2003; Phan et al., 2002; Vigneau et al., 2006; Schirmer et al., 2012) we considered the following hypothesis based on null hypothesis statistical testing; where H_0_ was true, where ES = 0. That ES was no different than zero and acceptance of H_a_ where ES ≠ 0 for small ES of ≈0.2, medium ES of ≈0.5, and large effect of ≈0.8 (Cohen, 1992; Strube, 1985). Effect size statistics were calculated for: 1) amygdala functioning for hemispheric lateralization where left hemisphere effect was different than and not equal to a right hemisphere effect by sample size and 2) amygdala functioning for emotion (e.g. happy/sad) versus null effect (i.e, neutral) to determine stimuli salience. All power analysis utilized effect sizes taken from the literature.

Fig. S14 | Power analysis based on effect size from literature


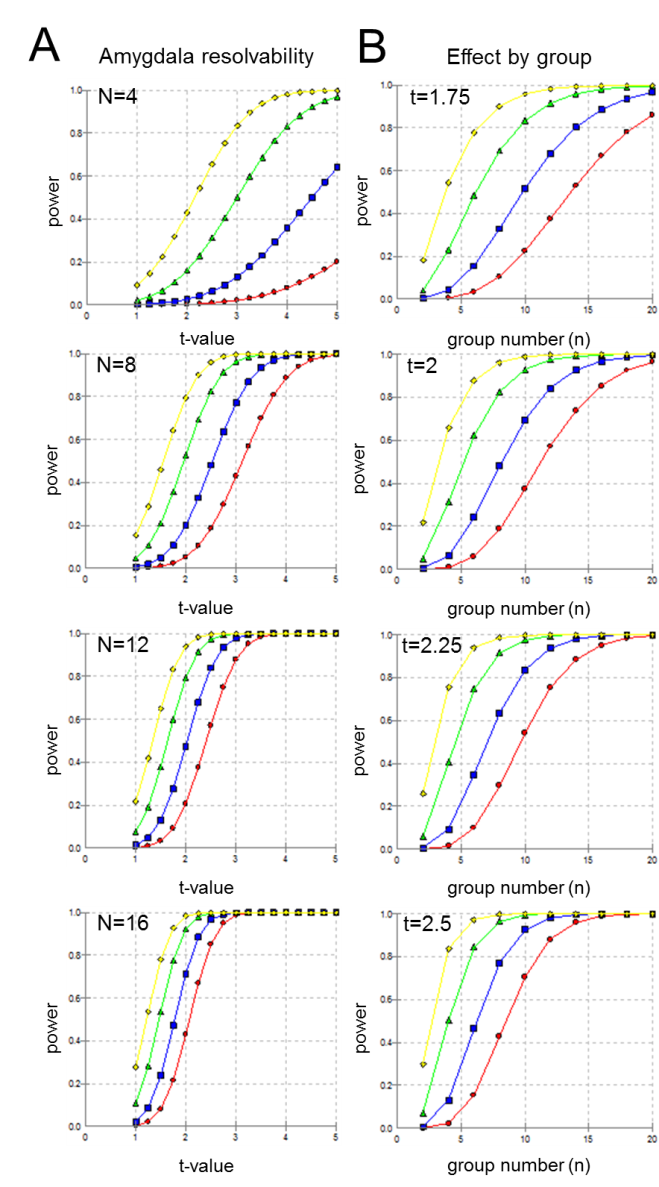


Power analysis calculations from Koelsch (2014, Nat Rev Neurosci. 15;170–180). Effect size statistics were calculated utilizing Koelsch, 2014 for amygdala hemispheric lateralization and amygdala functioning to emotional stimuli. Alpha was calculated for α = 0.05 (yellow), 0.01 (green), 0.001 (blue), 0.0001 (red) within a group. (**A**) Amygdala resolvability during emotion processing compared to null for hemispheric lateralization. For a group size n=10 at alpha α = 0.01 there was low probability of a Type 1 error (i.e. rejecting a false null hypothesis) based on previous literature. Our functional MRI study was n=16 for two runs where H_a_ was different from H_0_ based on significant activation or deactivation. Power is represented on the y-axis and t-value difference by category on the x-axis for n = 4, 8, 12, and 16. (**B**) Amygdala modulation to emotion observed for emotions such as fear, joy, happy, and sad stimuli different than neutral as reported in previous literature. We have the mean t-value difference in voxel space (>100 voxels) for the category of emotion factored by number needed. A group size n=10 provides a low probability of committing a Type I error.

Table S36 | Studies for determining effect size statistics of emotion

| # | Study | Original Values |
| --- | --- | --- |
| 1 | Koelsch et al. 2006,Hum Brain Mapp. 27:239–250. fMRI N=11. | Unpleasant > pleasant stimuli. AMG-L t=4.7 & AMG-R t=3.3, L-HC t=5.7, R-HC t=3.8. Pleasant > unpleasant HG-L t=4.9 & HG-R t=4.6; R-IFG t=5.1, L-IFG t=3.5 |
| 2 | Mitterschiffthaler et al., 2007, Hum Brain Mapp 28:1150-62. fMRI - N=16 | Happy-STG-L Ζ =4.96, STG-R Ζ =4.54, Sad STG-L Ζ =4.07, STG-R Ζ =3.99. Happy rating 79.82±13.51 and sad rating 34.55 ±17.23 with 100 and 0 indicating happy and sad, respectively. L-PHCG z=3.31, R-PHCG/AMG z=3.25. |
| 3 | Brattico et al., 2011, Front Psychol. 2011 2:308. fMRI - N=15 | sad music with lyrics <sad music without lyrics (t=2.6), happy music without lyrics > happy music with lyrics (t=2.7). Happy versus sad music STG-L Brodmann 22 Ζ=3.9 and Brodmann 42 Ζ =3.55.  Sad Music with lyrics vs. sad music without lyrics, L-amygdala Ζ =4.26, R-PHCG/AMG z=3.75; STG-R Ζ =4.87, STG-L Ζ =4.54. R-IFGz=4.22, L-IFG z=3.63. Happy music without Lyrics vs. happy with lyrics R-IFG z=3.98, L-SFG z=3.94. Effect of lyrics on sad music > happy STG-R Ζ=3.88, STG-L Ζ =3.98. L-IFG z=3.98, R-STG z=3.88, R-IFG z=3.64. [unfamiliar–familiar: χ2 (3) = 11.5, p < 0.01, sad–happy: χ2 (3) = 199.8, p < 0.0001, feeling sad–feeling happy: χ2 (3) = 115.3, p < 0.0001, disliked–liked: χ2 (3) = 27.4, p < 0.0001, unpleasant–pleasant: χ2 (3) = 33.02, p < 0.0001, ugly–beautiful: χ2 (3) = 38.1, p < 0.0001]. |
| 4 | Koelsch et al., 2008, Neuroreport. 19(18):1815-9. fMRI - N=12 non-musicians and N=12 musicians. | AMG-L p<0.05 and AMG-R p<0.002 to irregular chords, pleasantness/unpleasantness chords. Psycho: regular chord ratings 7.0±0.77 musicians & non-musicians 7.4±1.4. Valence for irregular chords 2.8±0.69 non-musicians and 3.6±1.34 musicians. ANOVA factor chord (regular/irregular) and group (musician/non) indicated a main effect of chord F_1,22_=136.9, P<0.0001, reflecting regular chords were perceived as more pleasant than irregular chords, small effect of group F_1,22_=136.9, P<0.059, reflecting that musicians tended to rate both regular and irregular endings as more pleasant than non-musicians. No values reported to discern effect sizes |
| 5 | Khalfa et al., 2005, Neuroreport. 16(18):1981-4. fMRI - N=13 | Happiness or sadness, on a 5-point scale of Western classical music in major in minor. Fast major excerpts were rated as the happiest excerpts (mean=4.2±0.5) followed by fast minor (3.4±0.4), slow major (mean=2.7±0.3) and slow minor excerpts (mean=1.9±0.3). Contrasting minor with major excerpts revealed activations in the left medial (BA 10) and superior (BA 9) frontal gyri and in both the right and left posterior cingulum gyri (BA 31). R & L BA31 Ζ =5.5, L-BA10 Ζ=5.1, L-BA9 Ζ =5.2. Interaction contrast for mode and tempo conditions also showed general significant activations in the left medial frontal gyrus (BA 9), in the right middle frontal gyrus (BA 6) and in the right anterior cingulate gyrus (BA 24). L-BA9 Ζ=5.3, R-BA6 Ζ =5.0, R-BA24 Ζ =5.2. N=13 |
| 6 | Suzuki et al., 2008, Cogn Affect Behav Neurosci. 8(2):126-31. PET – N=13 | 96 chord sequences—48 major-key consonant and dissonant chords and 48 minor-key consonant and dissonant chords. bilateral middle frontal gyrus Z=5.5 and bilateral superior temporal gyrus Ζ=3.84. Beautiful chords dorsomedial midbrain Ζ =3.97, ugly dissonance right inferior partietal lobule Ζ =3.56, left insula Ζ =3.3 and left frontal subgyral Ζ =3.30. Minor key vs. major key L-PHC z=3.45. B–majC vs. B–minC Left-Middle temporal gyrus Ζ =3.46. B–majC vs. U–majD Left-Middle temporal gyrus Ζ =3.77. B–minC vs. U–mind, L-Dorsomedial midbrain Z=3.92, R-IFG Z=3.54, L-ACG Z=3.35. Where, beautiful, major-key consonant (B–majC) chords; beautiful, minor-key consonant (B–minC) chords; ugly, major-key dissonant (U–majD) chords; and ugly, minor-key dissonant (U–minD) chords |
| 7 | Salimpoor et al., 2011, Nat Neurosci. 14(2):257-62. PET & fMRI – N=8 | Greater pleasure was experienced during the pleasurable music condition over the neutral music condition (t=25.0, P < 0.001), during peak pleasure experience compared with neutral, there was increased BOLD response in the R-NAcc (t = 2.8). Increased BOLD response was also found during the anticipation largely confined to the right caudate ( z = 14; t = 3.2). |
| 8 | Blood et al. 1999. Nat Neurosci. 2(4):382-7. PET, N=10. | Degree of dissonance was varied establishing pleasant and unpleasant stimuli. Positive correlations denote increasing dissonance; negative correlations denote increasing consonance. Dissonant minus noise R-STG t=3.55, L-STG t=3.69, R-PHCG t=2.86; Consonant minus noise R-STG t=3.7, L-STG t=3.42, R-PHCG t=-3.48. |
| 9 | Chapin et al., 2010. PLoS One. 5(12):e13812. fMRI N=21 | Chopin altered excerpts. Expressive music performance, skilled pianist with natural variations in timing and sound intensity, and a mechanical performance to control for compositional aspects of the stimulus such as average values of tempo and sound intensity. Expressive V. Mechanical: R-PHC F=18.94, R-IFG f=9.63, L-SFG f=8.3. Experienced V inexperienced L-PHC f=-8.16. Tempo: L-SFG T=2.61, R-ST t=2.99. Emotional arousal: experienced V inexperienced. L-IP t=3.15, R-IP t=3.00, R-SFG t=3.39 |
| 10 | Koelsch & Skouras 2014, Hum Brain Mapp. 35(7):3485-98. fMRI, N=20 | Joy, fear and neutral. Joy > Fear: L-AMG-SP z=3.38, R-AMG-LB z=3.46, L-HPC Z=3.53, L-AMG-LB z=3.7, R-HPC z=3.53. P: Joy 4.9±0.2, fear 3.5±0.3. Joy ratings, higher for joy than neutral t=4.92, and higher for neutral than fear t=2.55. Fear ratings, higher for fear than neutral t=3.71 and higher for neutral than joy t=5.08. |
| 11 | Eldar et al., 2007 Cereb Cortex. 17(12):2828-40. fMRI, n=14 | Visual and auditory stimuli. Music clips were of positive (joyful), negative (scary), or neutral (simple and monotonic) emotional tones. 1) film clips were of emotionally neutral character; 2) coupled with emotional music, interpreted as both negative and positive; 3) film clips contained no dialogue; and 4) film clips portrayed no widely familiar actors or scenes. Negative combinations R-AMG-AL t=4.81, R-IFG BA47 t=4.08, R-ITGBA37 t=4.29, Positive combination: R-IFG BA47 t=4.40, R-STG BA22 t=4.68, R-ITG BA21 t=4.37, L-ITG t=4.16. Neutral: R-IFG BA45 t=4.43. |
| 12 | Caria et al., 2011, Cereb Cortex. 21(12):2838-49. fMRI, N=22 | Classical musical pieces from 18th, 19th, and 20th century Western, herein named ‘‘standard” and preferred happy and sad musical pieces selected by the participants, herein named ‘‘favorite. Happy, standard and favorite>control stimuli: R-IFG t=5.27, L-STG t=5.26, R-STG t=4.20, L-IFG t=4.05, R-IFG t=3.98. Happy, favorite>standard: R-SFG t=7.11, R-HG t=6.05, L-STG t=5.38 Sad, Standard and favorite>control stimuli: L-insula t=4.97, R-insula t=4.95, L-IFG t=4.70. Autism spectrum disorder versus controls. |
| 13 | Koelsch et al., 2013, Neuroimage. 2013 Nov 1;81:49-60. | joy>fear: L-HG z=3.76, R-HG t=3.72, L-AMG-SP t=3.36, R-AMG-SP t=3.09. Positive z-values (outermost right column) indicate stronger functional connectivity during joy compared to fear, whereas negative z-values indicate stronger functional connectivity during fear compared to joy. Left auditory cortex - L-HG z=3.17, R-HG t=3.16. Right auditory cortex – L-PT z=3.22, R-PT z=3.13. Left SP-AMG – R-PT Z=3.75. Right SP-AMG – MD thalamus z=3.22. |

N-number of individuals in the study. L-left, R-right, P-psychophysically, STG-superior temporal gyrus, ACG-anterior cingulate gyrus, NAcc- nucleus accumbens. IFG – Inferior frontal gyrus, PHCG-Parahippocampal gyrus, HC-Hippocampus. SFG-superior frontal gyrus, IP-inferior parietal. AL-anterolateral, PT-planum temporale.

# References

Beckmann CF, Jenkinson M, Smith SM. (2003). General multilevel linear modeling for group analysis in FMRI. Neuroimage. 20:1052-63.

Binder JR, Liebenthal E, Possing ET, Medler DA, Ward BD. (2004). Neural correlates of sensory and decision processes in auditory object identification. Nat Neurosci. 7(3):295-301.

Blood AJ, Zatorre RJ, Bermudez P, Evans AC. (1999). Emotional responses to pleasant and unpleasant music correlate with activity in paralimbic brain regions. Nat Neurosci. 2(4):382-7.

Brainard DH. (1997). The Psychophysics Toolbox. Spatial Vision. 10:443-446. <http://psychtoolbox.org/>

Brattico E, et al. (2011). A functional MRI study of happy and sad emotions in music with and without lyrics. Front Psychol 2:308.

Brimijoin O. (2012). Oscillator. File Exchange, MATLAB Central. <http://www.mathworks.com/matlabcentral/fileexchange/37376-oscillator-and-signal-generator>

Caria A, Venuti P, de Falco S. (2011). Functional and dysfunctional brain circuits underlying emotional processing of music in autism spectrum disorders. Cereb Cortex. 21(12):2838-49.

Chapin H, Jantzen K, Kelso JA, Steinberg F, Large E. (2010). Dynamic emotional and neural responses to music depend on performance expression and listener experience. PLoS One. 5(12):e13812.

Cohen J. (1988). Statistical Power Analysis for the Behavioral Sciences (2nd Ed). Lawrence Earlbaum Associates, Hillsdale, NJ.

Cohen J. (1992). A power primer. Psychol Bull 112:155-9.

Da Costa S, van der Zwaag W, Miller LM, Clarke S, Saenz M. (2013) Tuning in to sound: frequency-selective attentional filter in human primary auditory cortex. J Neurosci. 33:1858-63.

Dalla Bella S, Peretz I, Rousseau L, Gosselin N, Ayotte J, Lavoie A. (2001a) Development of the happy-sad distinction in music appreciation. Does tempo emerge earlier than mode? Ann N Y Acad Sci. 930:436-8.

Dalla Bella S, Peretz I, Rousseau L, Gosselin N. (2001b) A developmental study of the affective value of tempo and mode in music. Cognition. 80(3):B1-10.

Edmister WB, Talavage TM, Ledden PJ, Weisskoff RM. (1999). Improved auditory cortex imaging using clustered volume acquisitions. Hum Brain Mapp. 7(2):89-97.

Eklund A, Nichols TE, Knutsson H. (2016). Cluster failure: Why fMRI inferences for spatial extent have inflated false-positive rates. Proc. Natl. Acad. Sci. U.S.A. 113:7900-7905.

Eldar E, Ganor O, Admon R, Bleich A, Hendler T. (2007). Feeling the real world: limbic response to music depends on related content. Cereb Cortex. 17(12):2828-40.

Fisher SZ, Student ST. (2012). A triple dissociation of neural systems supporting ID, EGO, and SUPEREGO. Psyence. 335:1669.

Friston K. (2012). Ten ironic rules for non-statistical reviewers. Neuroimage 61:1300-1310.

Friston KJ, Holmes AP, Poline JB, Grasby PJ, Williams SC, Frackowiak RS, Turner R (1995) Analysis of fMRI time-series revisited. Neuroimage 2:45–53.

Friston, K., Holmes, A., Poline, J., Price, C., and Frith, C. (1996). Detecting activations in PET and fMRI: levels of inference and power. Neuroimage, 4(3 Pt 1), 223–235.

Fritz T, Jentschke S, Gosselin N, Sammler D, Peretz I, Turner R, Friederici AD, Koelsch S. (2009) Universal recognition of three basic emotions in music. Curr Biol. 19:573-6.

Frühholz S, et al. (2005) Asymmetrical effects of unilateral right or left amygdala damage on auditory cortical processing of vocal emotions. Proc Natl Acad Sci U S A. 112:1583-1588.

Genovese, C., Lazar, N., and Nichols, T. (2002). Thresholding of statistical maps in functional neuroimaging using the false discovery rate. Neuroimage, 15(4), 870–878.

Geschwind N, Galaburda AM. (1985) Cerebral lateralization. Biological mechanisms, associations, and pathology: I. A hypothesis and a program for research. Arch Neurol. 42(5):428-59. (The second and third article in the series providing ancillary information).

Gosselin N, et al. (2005). Impaired recognition of scary music following unilateral temporal lobe excision. Brain 128:628-40.

Gosselin N, Peretz I, Johnsen E, Adolphs R. (2007). Amygdala damage impairs emotion recognition from music. Neuropsychologia 45, 236–244.

Hall DA, Haggard MP, Akeroyd MA, Palmer AR, Summerfield AQ, Elliott MR, Gurney EM, Bowtell RW. (1999). "Sparse" temporal sampling in auditory fMRI. Hum Brain Mapp. 7(3):213-23.

Jansen A, et al. Menke R, Sommer J, Förster AF, Bruchmann S, Hempleman J, Weber B, Knecht S. (2006). The assessment of hemispheric lateralization in functional MRI--robustness and reproducibility. Neuroimage. 33(1):204-17.

Kawakami A, Furukawa K, Katahira K, Okanoya K. (2013). Sad music induces pleasant emotion. Front Psychol. 4:311.

Khalfa S, Roy M, Rainville P, Dalla Bella S, Peretz I. (2008). Role of tempo entrainment in psychophysiological differentiation of happy and sad music? Int J Psychophysiol. 68(1):17-26.

Khalfa S, Schon D, Anton JL, Liégeois-Chauvel C. (2005). Brain regions involved in the recognition of happiness and sadness in music. Neuroreport 16:1981–1984.

Khalfa S, Schon D, Anton JL, Liégeois-Chauvel C. (2005). Brain regions involved in the recognition of happiness and sadness in music. Brain regions involved in the recognition of happiness and sadness in music. Neuroreport. 16(18):1981-4.

Khalfa S, Schon D, Anton JL, Liégeois-Chauvel C. (2005). Brain regions involved in the recognition of happiness and sadness in music. Neuroreport. 16(18):1981-4.

Koelsch S, Fritz T, Cramon DY, Müller K, Friederici AD. (2006). Investigating emotion with music: an fMRI study. Hum. Brain Mapp. 27:239–250.

Koelsch S, Fritz T, Schlaug G. (2008). Amygdala activity can be modulated by unexpected chord functions during music listening. Neuroreport. 19(18):1815-9.

Koelsch S, Skouras S, Fritz T, Herrera P, Bonhage C, Küssner MB, Jacobs AM. (2013). The roles of superficial amygdala and auditory cortex in music-evoked fear and joy. Neuroimage. 81:49-60.

Koelsch S, Skouras S. (2014). Functional centrality of amygdala, striatum and hypothalamus in a "small-world" network underlying joy: an fMRI study with music. Hum Brain Mapp. 35(7):3485-98.

Koelsch S. (2005). Investigating emotion with music: Neuroscientific approaches. ‎Ann NY Acad Sci. 1060: 1–7.

Koelsch S. (2010). Towards a neural basis of music-evoked emotions. Trends Cogn Sci. 14(3):131-7.

Koelsch S. (2014). Brain correlates of music-evoked emotions. Nat Rev Neurosci 15:170-80.

Kumar S, von Kriegstein K, Friston K, Griffiths TD (2012) Features versus feelings: Dissociable representations of the acoustic features and valence of aversive sounds. J Neurosci 32:14184–14192.

Leaver AM, Rauschecker JP. (2010) Cortical representation of natural complex sounds: effects of acoustic features and auditory object category. J Neurosci 30:7604-12.

Mazziotta JC, et al. (2001) A probabilistic atlas and reference system for the human brain: International Consortium for Brain Mapping (ICBM). Phil Trans R Soc B 356:1293-1322.

Micheyl C, Kaernbach C, Demany L. (2008). An evaluation of psychophysical models of auditory change perception. Psychol Rev. 115(4):1069-83.

Mitterschiffthaler MT, Fu CH, Dalton JA, Andrew CM, Williams SC. (2007) A functional MRI study of happy and sad affective states induced by classical music. Hum Brain Mapp 28:1150-62.

Moon IJ, Won JH, Park MH, Ives DT, Nie K, Heinz MG, Lorenzi C, Rubinstein JT. (2014) Optimal combination of neural temporal envelope and fine structure cues to explain speech identification in background noise. J Neurosci. 34:12145-54.

Mutschler I, Wieckhorst B, Speck O, Schulze-Bonhage A, Hennig J, Seifritz E, Ball T. (2010). Times scales of auditory habituation in the amygdala and cerebral cortex. Cereb Cortex. 20(11):2531-9.

Norman-Haignere S, Kanwisher N, McDermott JH. (2013) Cortical pitch regions in humans respond primarily to resolved harmonics and are located in specific tonotopic regions of anterior auditory cortex. J Neurosci 33:19451-69.

Oldfield, RC. (1971). The assessment and analysis of handedness: the Edinburgh inventory. Neuropsychologia. 9(1):97-113.

Overath T, McDermott JH, Zarate JM, Poeppel D. (2015) The cortical analysis of speech-specific temporal structure revealed by responses to sound quilts. Nat Neurosci. 18:903-11.

Peretz I, Gagnon L, Bouchard B. (1998a) Music and emotion: perceptual determinants, immediacy, and isolation after brain damage. Cognition. 68(2):111-41.

Peretz I, Gaudreau D, Bonnel AM. (1998b) Exposure effects on music preference and recognition. Mem Cognit. 26(5):884-902.

Peretz I, Gosselin N, Nan Y, Caron-Caplette E, Trehub SE, Béland R. (2013) A novel tool for evaluating children's musical abilities across age and culture. Front Syst Neurosci. 7:30 (Montreal Battery of Evaluation of Musical Abilities, MBEMA)

Perrachione TK, Ghosh SS. (2013). Optimized design and analysis of sparse-sampling FMRI experiments. Front Neurosci. 18;7:55.

Pfeifer R. (1988). Artificial intelligence models of emotion. Cognitive Perspectives on Emotion and Motivation, eds Vernon Hamilton, Gordon H. Bower, Nico H. Frijda (Springer Netherlands) pp 287-320.

Phan KL, Wager T, Taylor SF, Liberzon I. (2002) Functional neuroanatomy of emotion: A meta-analysis of emotion activation studies in PET and fMRI. Neuroimage 16:331-48.

Rouder JN, Morey RD. (2009). The nature of psychological thresholds. Psychol Rev. 116(3):655-60.

Salimpoor VN, Benovoy M, Larcher K, Dagher A, Zatorre RJ. (2011). Anatomically distinct dopamine release during anticipation and experience of peak emotion to music. Nat Neurosci. 14(2):257-62.

Schirmer A, Fox PM, Grandjean D. (2012). On the spatial organization of sound processing in the human temporal lobe: A meta-analysis. Neuroimage 63:137-147.

Smith ZM, Delgutte B, Oxenham AJ. (2002) Chimaeric sounds reveal dichotomies in auditory perception. Nature 416:87-90.

Strube MJ. (1985). Power analysis for combining significance levels. Psychological Bulletin 98:595-599.

Suzuki M, Okamura N, Kawachi Y, Tashiro M, Arao H, Hoshishiba T, Gyoba J, Yanai K. (2008). Discrete cortical regions associated with the musical beauty of major and minor chords. Cogn Affect Behav Neurosci. 8(2):126-31.

Talavage TM, Edmister WB, Ledden PJ, Weisskoff RM. (1999). Quantitative assessment of auditory cortex responses induced by imager acoustic noise. Hum Brain Mapp. 7(2):79-88.

Tomasi D, Volkow ND. (2012) Laterality patterns of brain functional connectivity: Gender effects. Cereb Cortex. 22:1455-1454.

Treisman A. (1996). The binding problem. Curr Opin Neurobiol. 6:171-178.

Treisman A. (1999). Solutions to the binding problem: progress through controversy and convergence. Neuron. 24:105-110.

Vigneau M., et al. (2006). Meta-analyzing left hemisphere language areas: Phonology, semantics, and sentence processing. Neuroimage 30:1414-1432.

Wager TD, Phan KL, Liberzon I, Taylor SF. (2003) Valence, gender, and lateralization of functional brain anatomy in emotion: a meta-analysis of findings from neuroimaging. Neuroimage 19:513-31.

Wilkinson L, APA Task Force on Statistical Inference. (1999). Statistical methods in psychology journals: guidelines and explanations. Am. Psychol. 54 (8), 594–604.

Witteman J, Van Heuven VJ, Schiller NO. (2012) Hearing feelings: A quantitative meta-analysis on the neuroimaging literature of emotional prosody perception. Neuropsychologia 50:2752-63.

Wojcicki K. (2001) Tone Generator. File Exchange, MATLAB Central. <http://www.mathworks.com/matlabcentral/fileexchange/34058-pure-tone-generator>

Woolrich MW, Behrens TE, Beckmann CF, Jenkinson M, Smith SM. (2004) Multilevel linear modelling for FMRI group analysis using Bayesian inference. Neuroimage. 21:1732-47.

Zar JH. (1999) Biostatistical analysis. Fourth Edition. Prentice Hall, New Jersey.
